# New naphthalene-linked pyrazoline–thiazole hybrids as prominent antilung and antibreast cancer inhibitors

**DOI:** 10.55730/1300-0527.3704

**Published:** 2024-11-18

**Authors:** Halilibrahim ÇİFTÇİ, Masami OTSUKA, Mikako FUJITA, Belgin SEVER

**Affiliations:** 1 Department of Bioengineering Sciences, İzmir Katip Çelebi University, İzmir, Turkiye; 2 Medicinal and Biological Chemistry Science Farm Joint Research Laboratory, Faculty of Life Sciences, Kumamoto University, Kumamoto, Japan; 3 Department of Drug Discovery, Science Farm Ltd., Kumamoto, Japan; 4 Department of Molecular Biology and Genetics, Burdur Mehmet Akif Ersoy University, Burdur, Turkiye; 5 Department of Pharmaceutical Chemistry, Faculty of Pharmacy, Anadolu University, Eskişehir, Turkiye

**Keywords:** Naphthalene, pyrazoline, thiazole, epidermal growth factor receptor, nonsmall cell lung cancer, breast cancer

## Abstract

The epidermal growth factor receptor (EGFR) and human epidermal growth factor receptor 2 (HER2), pioneer members of the receptor tyrosine kinase subfamily, are frequently mutated and/or overexpressed in several types of human cancers, including nonsmall cell lung cancer (NSCLC) and breast cancer, which are leading causes of cancer-related deaths worldwide. EGFR and HER2-focused anti-NSCLC and antibreast cancer studies encouraged us to search for new potential agents. For this purpose, in the current work, naphthalene-linked pyrazoline–thiazole hybrids (**BTT-1**–**10** and **BTP-1**–**10**) were synthesized and examined for their antiproliferative effects on A549 NSCLC and MCF-7 breast cancer cell lines. According to the results, the MTT assay showed that **BTT-5** induced strong toxicity in A549 cells with an IC_50_ value of 9.51 ± 3.35 μM compared to lapatinib (IC_50_ = 16.44 ± 3.92 μM). **BTT-5** also presented a high selectivity profile between the Jurkat cell line and PBMCs (healthy) (SI = 65.65). Furthermore, **BTT-5** augmented apoptosis significantly in A549 cells (18.40%). **BTT-5** displayed significant EGFR inhibition (58.32%) and no significant HER2 inhibition at 10 μM concentration, showing its selective kinase inhibitory effects. The molecular docking assessment indicated that **BTT-5** showed high affinity with a different binding profile than lapatinib in the ATP binding cleft of EGFR. Consequently, **BTT-5** can serve as a lead for future anti-NSCLC studies.

## 1. Introduction

Lung cancer, which is generally divided into two main clinical types, small cell lung cancer (SCLC) and nonsmall cell lung cancer (NSCLC), is one of the most common and deadly cancers in the world. NSCLC is commonly diagnosed at advanced stages, when surgical resection is inadequate in the majority of patients. Chemotherapy and radiotherapy are other therapeutic strategies for NSCLC but their effectiveness is relatively insufficient. Moreover, chemotherapy reduces the patients’ quality of life and causes unexpected deaths and short survival rates. Therefore, new therapeutic options such as the targeted therapy using advanced cellular and molecular methods have been developed. This therapy targets the specific oncogenes and signaling pathways that play a critical role in gene expression, apoptosis, and the cell cycle. In recent years, the development of specific tyrosine kinase (TK) inhibitors and the examination of these agents at molecular levels have led to fundamental changes in the treatment of NSCLC [1–4].

The epidermal growth factor receptor (EGFR/ERbB1) is a key driver of NSCLC carcinogenesis. The EGFR, together with the other members human epidermal growth factor receptor 2 (HER2/ERbB2/Neu), HER3/ERbB3, and HER4/ERbB4, is a member of the ErbB family of receptor tyrosine kinases (RTKs) [5–7]. Each of them consists of an extracellular ligand-binding domain, a single hydrophobic transmembrane region, and an intracellular TK domain. Upon binding of the ligand to the extracellular domain, homo- or heterodimerization of EGRF leads to autophosphorylation of the intracellular domain through TK activity and subsequent stimulation of a downstream cascade that can lead to proliferation, metastasis, suppression of apoptosis, and angiogenesis [8]. Aberrant activation of the EGFR, arising from autocrine ligand–receptor stimulation, point mutations, deletions, and gene amplification, is associated with a poor prognosis in a large number of human malignant tumors. Therefore, inhibition of the EGFR could generate substantial therapeutic benefits in anticancer treatment [9,10]. For this purpose, small-molecule EGFR inhibitors, which are capable of occluding the adenosine triphosphate (ATP) binding site of the intracellular TK domain, have been developed [11,12]. EGFR TK inhibitors approved for the treatment of advanced NSCLC include afatinib, dacomitinib, erlotinib, gefitinib, almonertinib, brigatinib, icotinib, olmutinib, and osimertinib [13,14]. Lapatinib, on the other hand, is a reversible and potent dual EGFR and HER2 inhibitor that is approved in combination with capecitabine for the treatment of breast cancer, one of the most common and deadly cancers in women [15,16]. Breast cancer is prone to metastasize to the lungs and bones easily; 60% of the deaths from breast cancer metastasis stem from lung metastasis and the survival rate is less than 25 months [17]. HER2 is predominantly overexpressed in breast cancer. However, EGFR/HER2 heterodimers are also important targets for antilung cancer therapy. The Lung Cancer Mutation Consortium (LCMC) has declared that 3% of patients with lung cancer harbor HER2 mutations [18].

**Figure 1:**
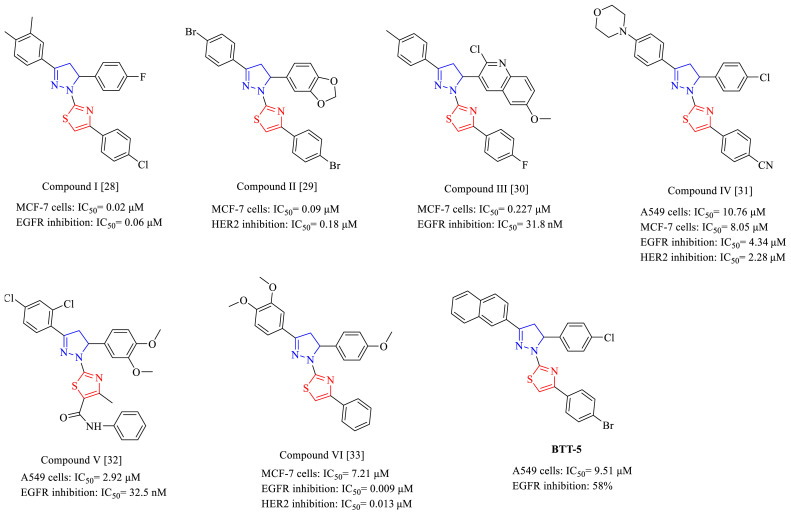
The potential anticancer agents and EGFR and/or HER2 inhibitors that have a pyrazoline–thiazole core.

Pyrazoline, also known as dihydropyrazole, is a heterocyclic ring that contains an endocyclic π-bond and two adjacent nitrogen atoms. Depending on the position of this endocyclic bond, there are three isomers of pyrazolines, of which 2-pyrazoline (4,5-dihydro-1*H*-pyrazole) is the most common and stable. In particular, 3,5-diaryl-4,5-dihydro-1*H*-pyrazole derivatives have been extensively screened for a wide range of biological activities, including anticancer activity [19–22]. In addition, thiazole, a versatile scaffold in medicinal chemistry, has an electron-donating sulfur atom and an electron-withdrawing nitrogen atom (–C=N). Thiazole has been pioneering in the design and synthesis of numerous biologically active compounds endowed with potent anticancer properties targeting specific pathways [23–27]. Pyrazoline–thiazole hybridization has also been implicated to have potential anticancer activity, inhibiting EGFR and/or HER2 [28–33]. Compound I [28] (Figure 1) and compound II [29] (Figure 1) revealed antibreast cancer effects related to EGFR (IC_50_= 0.06 μM) and HER2 inhibition (IC_50_= 0.18 μM), respectively. Furthermore, compound III [30] (Figure 1) exhibited antibreast cancer activity associated with EGFR inhibition with an IC_50_ value of 31.80 nM. We also identified that compound IV [31] (Figure 1) inhibited EGFR (IC_50_ = 4.34 μM) and HER2 (IC_50_ = 2.28 μM) and showed significant anti-NSCLC and antibreast cancer effects. Compound V [32] (Figure 1) was detected to show anti-NSCLC effects and EGFR inhibition (IC_50_ = 32.5 nM), whereas compound VI [33] (Figure 1) inhibited both EGFR (IC_50_ = 0.009 μM) and HER2 (IC_50_ = 0.013 μM), and demonstrated potential antibreast cancer activity.

In addition, naphthalene-linked compounds have been reported to elicit targeted anticancer activity attributable to its feasible aromatic conjugated system due to the presence of a fused pair of benzene rings [34–38]. Naphthalene-linked pyrazoline–thiazole hybridization made a diverse contribution, showing antidepressant and anticonvulsant [39], antimicrobial [40,41], antiproliferative [41], analgesic, and antiinflammatory activities [42].

The main goals of the current work were to combine naphthalene with the pyrazoline–thiazole framework and determine the potential anticancer effects of this combination against breast cancer and NSCLC.

In the light of the above findings, herein, we conducted an array of synthesis, in vitro, and in silico analyses to determine the anti-NSCLC and antibreast cancer activity of new naphthalene-linked pyrazoline-thiazole hybrids (**BTT-1**–**10** and **BTP-1**–**10**) as well as **BTT-3**, **BTT-4**, and **BTT-7**, which were previously identified and their potential analgesic and antiinflammatory effects were investigated [42].

Initially, we synthesized new hybrids via the application of Hantzsch condensation on naphthalene-based dihydropyrazole carbothioamides (**B1** and **B2**), which were obtained via the reaction of chalcones (**A1** and **A2**) and thiosemicarbazide under basic conditions. Then we carried out the MTT assay to screen the antiproliferative effects of new hybrids against A549 human NSCLC and MCF-7 human breast cancer cell lines. On the basis of the results, the tested compounds that had the best cytotoxicity were selected for further biological testing, including evaluation of their cytotoxic effects on healthy cells (peripheral blood mononuclear cells (PBMCs)), their apoptotic effects, and their EGFR and HER2 inhibitory activity compared to lapatinib. To compare the in vitro and in silico results, molecular docking studies were utilized for **BTT-1**–**10** and **BTP-1**–**10** in the ATP binding site of the EGFR.

## 2. Materials and methods

### 2.1. Chemistry

All reagents were commercially purchased and used without purification unless otherwise stated. Thin layer chromatography (TLC) was performed using silica gel 60 F254 aluminum sheets (Merck, Darmstadt, Germany). ^1^H NMR and ^13^C NMR spectra were detected by a Bruker NMR spectrometer (Bruker, Billerica, MA, USA), whereas mass spectra (electron ionization) and high-resolution mass spectra (HRMS) were detected by JEOL JMS-700 Station/JMS-BU-20-GCmate (JEOL, Akishima, Tokyo).

#### 2.1.1. General procedure for the synthesis of the compounds

##### 2.1.1.1. 3-(4-Chlorophenyl)-1-(naphthalen-2-yl)prop-2-en-1-one (**A1**) and 3-(4-chlorophenyl)-1-(2-methoxynaphthalen-1-yl)prop-2-en-1-one (**A2**)

4-Chlorobenzaldehyde (1 mmol) was added to a mixture of appropriate ketone (1 mmol) and NaOH (1.1 mmol) in ethanol. Then the final mixture was stirred for 24 h at room temperature (rt). After checking with TLC, the mixture was poured into crushed ice. The precipitate was filtered, washed with water, and dried. The final product was crystallized from ethanol [43–46] (Spectral Data: Supplementary Information. Figures S1–S6).

##### 2.1.1.2. 5-(4-Chlorophenyl)-3-(naphthalen-2-yl)-4,5-dihydro-1*H*-pyrazole-1-carbothioamide (**B1**) and 5-(4-chlorophenyl)-3-(2-methoxynaphthalen-1-yl)-4,5-dihydro-1*H*-pyrazole-1-carbothioamide (**B2**)

Chalcones (**A1** and **A2**) (3 mmol), thiosemicarbazide (4.5 mmol), and sodium hydroxide (3 mmol) were mixed in ethanol and refluxed for 8–12 h. The solution was poured into crushed ice and washed with water after cooling. The precipitate was filtered, washed with water, and dried. The final product was crystallized from ethanol [29,47] (Spectral Data: Supplementary Information. Figures S7–S12).

##### 2.1.1.3. 1-(4-(4-(Substitutedphenyl)thiazole-2-yl)-3-(naphthalen-2-yl/2-methoxynaphthalen-1-yl)-5-(4-chlorophenyl)-2-pyrazoline (**BTT-1**–**10** and **BTP-1**–**10**)

Compounds **B1** and **B2** (0.8 mmol) and 2-bromo-1-arylethanone (0.8 mmol) were heated in ethanol under reflux for 2–6 h. The precipitation was filtered, washed with ethanol, and dried. The final product was crystallized from ethanol. **BTT-3**, **BTT-4**, and **BTT-7** are recorded in the literature [42] (Spectral Data: Supplementary Information. Figures S13–S72).

### 2.2. Cytotoxicity

#### 2.2.1. Cell culture, drug treatment, and MTT assay

Dulbecco’s modified Eagle’s medium (DMEM) (Wako Pure Chemical Industries, Osaka, Japan) was used to culture A549 cells, while RPMI 1640 (Wako Pure Chemical Industries, Osaka, Japan) was used to culture MCF-7, Jurkat cells, and PBMCs (Precision Bioservices, Frederic, MD, USA). Both media included 10% fetal bovine serum (FBS) (Sigma Aldrich, MO, USA) and 89 μg/mL streptomycin (Meiji Seika Pharma, Tokyo, Japan). The cells were incubated at 37 °C in a humidified 5% CO_2_ atmosphere and plated on 24- and 96-well tissue plates (Iwaki brand Asahi Glass Co., Chiba, Japan) at a density of 2 × 10^4^ (for A549 and MCF-7 cells), 4 × 10^4^ (for Jurkat cells), and 1 × 10^6^ (for PBMCs) cells/mL and then incubated for 72 h (the optimum cell number was determined in relation to our previous studies [31,48–50]). Stock solutions of **BTT-1**–**10**, **BTP-1**–**10**, and lapatinib were prepared in DMSO (Wako Pure Chemical Industries) at concentrations ranging from 0.1 to 10 mM. These solutions were further diluted with fresh culture medium and the final DMSO concentration was set at 1%. The effects of **BTT-1**–**10**, **BTP-1**–**10**, and lapatinib on cell viability were evaluated using the MTT (Dojindo Technologies, Kumamoto, Japan) (Dojindo Molecular Technologies, Kumamoto, Japan) assay as previously mentioned in the literature [31,48–50] and all experiments were performed in triplicate.

### 2.3. Detection of cell death

A549 cells were incubated with **BTT-5**, **BTT-8**, and lapatinib at their IC_50_ concentration for 24 h before the application of a cell death detection kit (PromoKine, Heidelberg, Germany) according to the manufacturer’s protocol. A549 cell lines were exposed twice to 1X binding buffer, a staining solution containing 50 μL of 1X binding buffer, 4 μL of solutions of FITC-Annexin V, ethidium homodimer III, and Hoechst 33342 and incubated for 30 min at rt in the dark. The cells were then analyzed using a Biorevo Fluorescence BZ-9000 fluorescence microscope (Keyence, Osaka, Japan) as described previously [51,52].

### 2.4. EGFR and HER2 inhibitory activities

The EGFR TK enzyme system from Promega (Promega V3831) was performed at 10 μM and 1 μM concentrations and the HER2 TK enzyme system from Promega (Promega V9381) was performed at 10 μM concentration as previously described [31,48–50]. Briefly, EGFR and HER2 TK and its substrates were diluted with solutions of 95 μL of 2.5X kinase buffer and 15 μL of 100 μM ATP, respectively. The kinase reaction was carried out with various concentrations (0.1–100 μM) of 4 μL of the analog solution, 8 μL of working stocks of kinase and ATP/substrate in the 384-well plate, whereas following 1.5 h of incubation at rt, the kinase activity was quantified using an ADP-Glo Kinase Assay (Promega Corporation) and the kinase inhibitory effects of **BTT-5**, **BTT-8**, and lapatinib at 1 mM and 10 mM concentrations were evaluated by a luminescence plate reader, Infinite M1000 (Tecan, Groding, Austria).

### 2.5. Molecular docking studies

The crystal structure of EGFR (PDB ID: 1M17) [53], obtained from the RCSB Protein Data Bank, was prepared for docking by the PrepWizard module of the software Maestro. Prime automatically added the missing chains and PropKa measured the protonation state at physiological pH. The LigPrep module was applied to **BTT-1**–**10**, **BTP-1**–**10**, and lapatinib for preparation with energy minimization at physiological pH. Maestro’s grid generation was used to determine the docking grid, which was then applied to docking experiments using Glide/XP docking protocols [54–56].

## 3. Results and discussion

Three subsequent synthetic steps were followed until new naphthalene-linked pyrazoline-thiazole hybrids (**BTT-1**–**10** and **BTP-1**–**10**) were obtained. Initially, chalcones (**A1** and **A2**) were afforded via Claisen–Schmidt condensation of ketones (1-(2-methoxynaphthalen-1-yl)ethan-1-one and 1-(naphthalen-2-yl)ethan-1-one) and 4-chlorobenzaldehyde. Afterwards, 4,5-dihydropyrazole-carbothioamides (**B1** and **B2**) were synthesized via the reaction of **A1** and **A2** with thiosemicarbazide. Finally, **BTT-1**–**10** and **BTP-1**–**10** were obtained at the end of the reaction of **B1** and **B2** with 2-bromo-1-arylethanones. Among **BTT-1**–**10** and **BTP-1**–**10**, **BTT-3**, **BTT-4**, and **BTT-7** are recorded in the literature [42] (Scheme).

**Figure 2:**
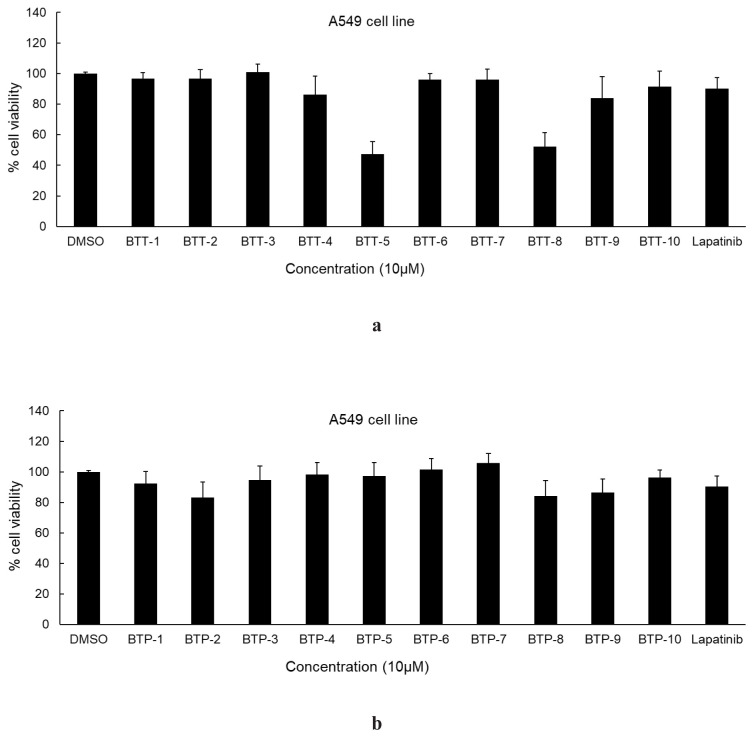
The cytotoxic effects of **BTT-1**–**10** (a) and **BTP-1**–**10** (b) on A549 cells compared to lapatinib at 10 μM concentration.

**Figure 3:**
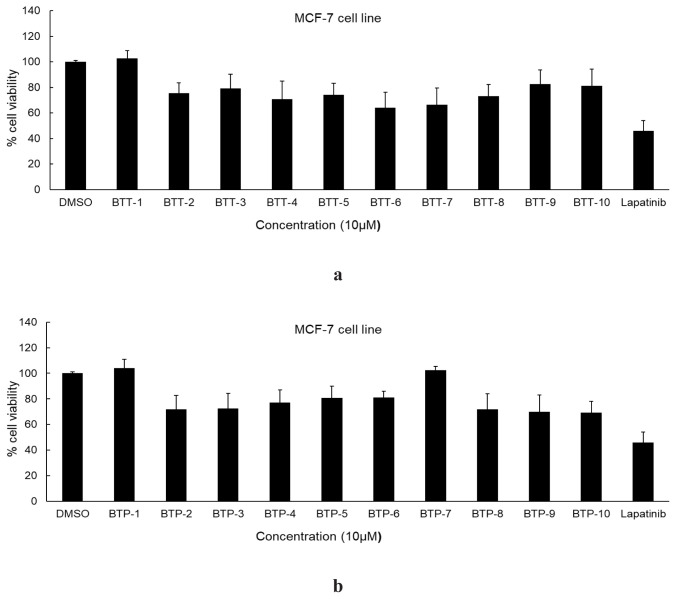
The cytotoxic effects of **BTT-1**–**10** (a) and **BTP-1**–**10** (b) on MCF-7 cells compared to lapatinib at 10 μM concentration.

The cytotoxic effects of **BTT-1**–**10** and **BTP-1**–**10** on A549 and MCF-7 cell lines were determined using the MTT assay. The anticancer activity of **BTT-1**–**10** was higher against lung cancer cells (Figure 2a) compared to that of **BTP-1**–**10** (Figure 2b). **BTT-1**–**10** also exhibited more significant anticancer effects (Figure 3a) on MCF-7 cancer cells compared to **BTT-1**–**10** (Figure 3b). **BTT-5** was identified as the most effective anticancer agent against A549 cells, with an IC_50_ value of 9.51 ± 3.35 μM compared to lapatinib (IC_50_ = 16.44 ± 3.92 μM). Lapatinib, a potential EGFR and HER2 inhibitor, was used as a standard agent. Following **BTT-5**, **BTT-8** revealed significant antilung cancer activity, with an IC_50_ value of 15.81 ± 4.71 μM. The IC_50_ values of **BTT-5** and **BTT-8** against MCF-7 cells were 54.42 ± 8.84 μM and 90.30 ± 10.25 μM, respectively, compared to lapatinib (IC_50_ = 8.55 ± 2.06 μM), indicating their moderate to low antibreast cancer effects (Table). Due to their significant anticancer potential, **BTT-5** and **BTT-8** were further evaluated to assess tumor selectivity between Jurkat cells and PBMCs. These compounds were significantly selective in PBMCs as depicted in the Table. In particular, the selectivity of the anticancer effects of **BTT-5** was higher, with a selectivity index (SI) value of 65.65. These outcomes highlighted that the 4-bromophenyl and 4-trifluoromethylphenyl substitutions on the thiazole core increased the antilung cancer effects of pyrazoline–thiazole hybrids as observed in **BTT-5** and **BTT-8**, respectively.

**Figure 4:**
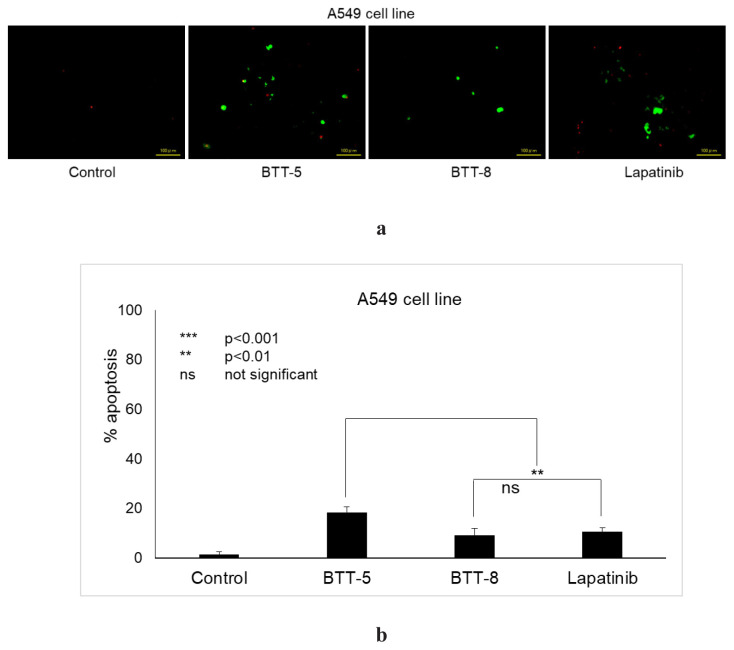
(a) The alterations in A549 cells after treatment with the control, **BTT-5**, **BTT-8**, and lapatinib for 24 h (b) defined with the analysis of 100 randomly selected stained cells in each experiment (***p < 0.001; ** p < 0.01; ns: not significant).

Since **BTT-5** and **BTT-8** were the most selective and effective anticancer agents, further mechanistic studies were performed for these compounds. To assess the apoptotic activity of **BTT-5** and **BTT-8** in the A549 cell line compared to lapatinib, the Annexin V staining method was used. A549 cells treated with **BTT-5**, **BTT-8**, and lapatinib were incubated, stained, and detected using a florescence microscope (Figure 4a). **BTT-5** showed significant apoptotic activity in A549 cells with 18.40% when compared with lapatinib (10.50%). On the other hand, the apoptotic level of **BTT-8** (9.20%) in A549 cells was weaker than that of **BTT-5** and lapatinib (Figure 4b).

**Figure 5:**
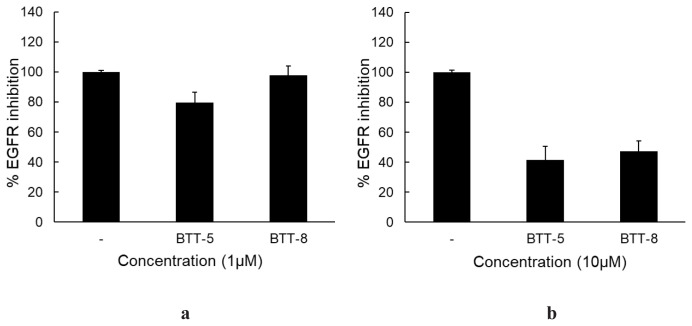
EGFR TK inhibitory effects of **BTT-5** and **BTT-8** at (a) 1 μM and (b) 10 μM concentrations.

**Figure 6:**
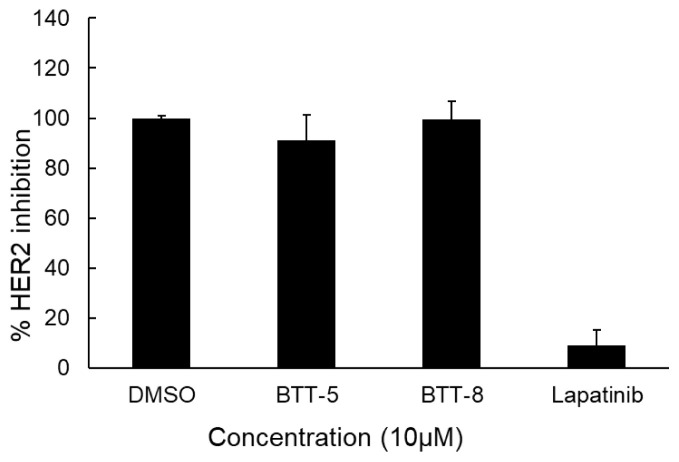
HER2 TK inhibitory effects of **BTT-5** and **BTT-8** at 10 μM concentration.

As **BTT-5** and **BTT-8** revealed the most promising antilung cancer effects among **BTT-1**–**10** and **BTP-1**–**10**, their EGFR and HER2 TK inhibitory effects were examined based on the importance of EGFR and HER2 in NSCLC pathogenesis. The results showed that both **BTT-5** and **BTT-8** displayed EGFR TK inhibition at 10 μM concentration with 58.32% and 52.69% (Figure 5a), respectively, though they showed no significant inhibition at 1 μM concentration (Figure 5b). On the other hand, **BTT-5** and **BTT-8** revealed no significant HER2 inhibition at 10 μM concentration, indicating the selectivity in their kinase inhibitory profiles (Figure 6).

In the literature, there is a distinct structural profile tethered to the pyrazoline–thiazole core in pyrazoline–thiazole carrying EGFR and/or HER2 inhibitors. In general, the halogen substitution increased the anticancer activity related to kinase inhibition as shown in compounds I–IV [28–32] (Figure 1). The 4-bromophenyl substitution at the pyrazoline–thiazole core contributed positively to the potential anticancer effects of compound II [29] and **BTT-5**.

**Figure 7:**
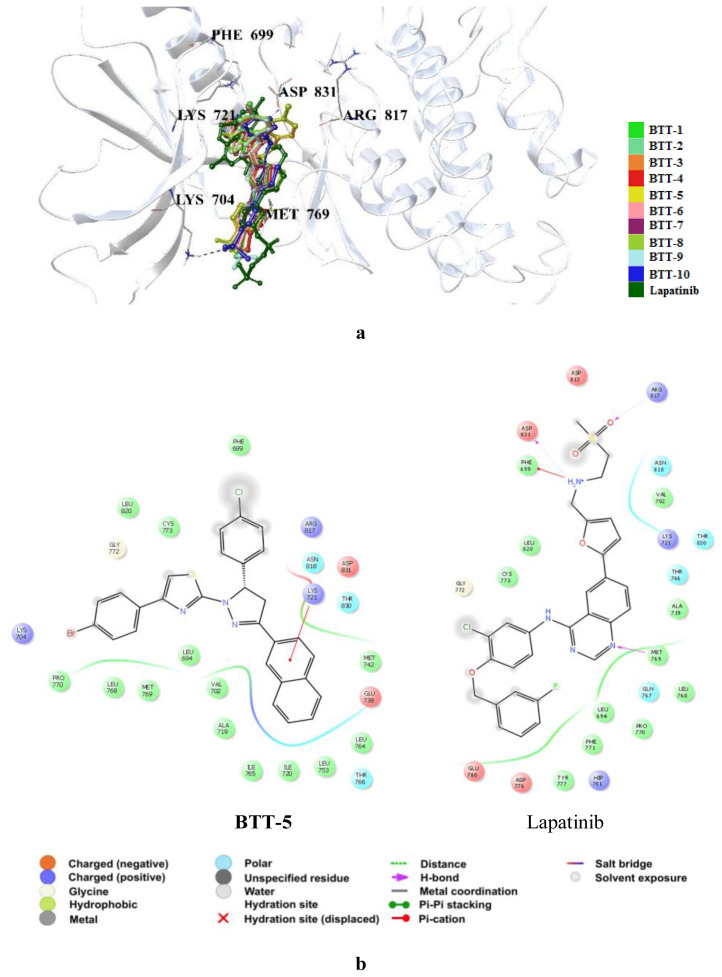
(a) Docking poses of **BTT-1**–**10** and lapatinib and (b) docking interactions of **BTT-5** and lapatinib in the ATP binding cleft of EGFR TK (PDB code: 1M17).

In order to understand the interactions of **BTT-1**–**10** and **BTP-1**–**10** in the ATP binding pocket of EGFR TK, molecular docking studies were assessed compared to lapatinib (PDB ID: 1M17) [53]. According to the results, **BTT-1**–**10** occupied the binding site of EGFR TK, forming distinct interactions from lapatinib (Figure 7a), whereas **BTP-1**–**10** revealed no interactions. **BTT-1**–**10** generally formed π-cation interactions with Lys721 through 2-naphthyl moiety (Figures 7a and 7b). The 4-bromophenyl substitution made no significant contribution to the EGFR TK binding profile of **BTT-5** (Figure 7b). Unlike lapatinib, **BTT-5** missed the substantial hydrogen bonding with Met769 in the ATP binding site of EGFR TK, which could unveil lower in vitro EGFR TK inhibitory potential of **BTT-5** compared to lapatinib.

## 4. Conclusion

The prognosis of patients with NSCLC and breast cancer still remains poor since these cancers are prone to confer resistance and subsequently recurrence. In an attempt to find effective, selective, and targeted anticancer agents, we synthesized new naphthalene-linked pyrazoline–thiazole hybrids and screened their anticancer activity against A549 and MCF-7 cell lines. Among these hybrids, **BTT-5** displayed prominent anticancer activity towards A549 cells and further induced apoptosis in A549 cells at high levels when compared with lapatinib. This compound displayed significant EGFR inhibitory activity and presented high affinity in the ATP binding site of the EGFR. All outcomes of in vitro and in silico analyses indicated that **BTT-5** could be a potential anti-NSCLC agent for future experiments.

## Supplementary Information



## Figures and Tables

**Scheme:**
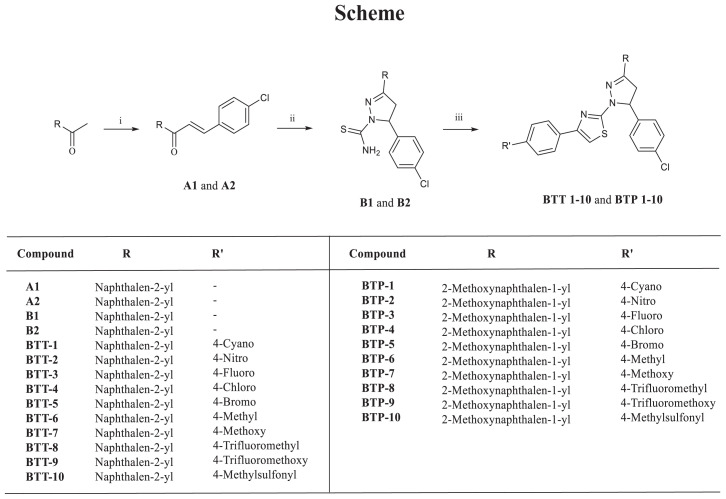
(i) 4-Chlorobenzaldehyde, NaOH, ethanol, rt, 24 h; (ii) Thiosemicarbazide, NaOH, ethanol, reflux, 8–12 h; (iii) 2-Bromo-1-arylethanone, ethanol, reflux, 2–6 h.

**Table:** The IC_50_ values of **BTT-5** and **BTT-8** against A549, MCF-7, Jurkat cells, and PBMCs compared to lapatinib.

Compound	IC_50_ values (μM)				SI*

	A549 cells	MCF-7 cells	Jurkat cells	PBMCs	
**BTT-5**	9.51±3.35	54.42±8.84	3.13±0.91	205.47±20.61	65.65
**BTT-8**	15.81±4.71	90.30±10.25	3.35±1.17	86.56±12.48	25.84

**Lapatinib**	16.44±3.92	8.55±2.06	1.42±0.36	8.80±2.23	6.20

* SI = IC_50_ for PBMC/IC_50_ for Jurkat cell line.
