# Supplementary material for: New naphthalene-linked pyrazoline–thiazole hybrids as prominent antilung and antibreast cancer inhibitors
Source: Turk J Chem. 2024 Nov 18;48(6):856–66. doi: 10.55730/1300-0527.3704 (PMC11706297; doi:10.55730/1300-0527.3704)
Supplement: Supplementary file 1 [file Supplementary_Information.pdf]

Supplementary Information

New naphthalene-linked pyrazoline-thiazole hybrids as prominent antilung and antibreast cancer inhibitors

Halilibrahim ÇİFTÇİ [1,2,3,4], Masami OTSUKA[2,3], Mikako FUJITA[2], Belgin SEVER[2,5,*]

[1] Department of Bioengineering Sciences, İzmir Katip Çelebi University, İzmir, Turkiye

[2] Medicinal and Biological Chemistry Science Farm Joint Research Laboratory, Faculty of Life Sciences, Kumamoto University, Kumamoto, Japan

[3] Department of Drug Discovery, Science Farm Ltd., Kumamoto, Japan

[4] Department of Molecular Biology and Genetics, Burdur Mehmet Akif Ersoy University, Burdur, Turkiye

[5] Department of Pharmaceutical Chemistry, Faculty of Pharmacy, Anadolu University, Eskişehir, Turkiye

ORCIDs:

Halilibrahim CIFTCI: https://orcid.org/0000-0002-9796-7669

Masami OTSUKA: https://orcid.org/0000-0002-2968-3939

Mikako FUJITA: https://orcid.org/0000-0001-6705-4052

Belgin SEVER: https://orcid.org/0000-0003-4847-9711

3-(4-Chlorophenyl)-1-(naphthalen-2-yl)prop-2-en-1-one (A1) [33]. Yield: 90%.^{1}H NMR (600 MHz, $CDCl_3$): 7.42 (2H, d, *J* = 8.04 Hz), 7.56-7.63 (4H, m), 7.67 (1H, d, *J* = 15.54 Hz), 7.82 (1H, d, *J* = 15.66 Hz), 7.90 (1H, d, *J* = 7.92 Hz), 7.95 (1H, d, *J* = 8.52 Hz), 8.00 (1H, d, *J* = 8.28 Hz), 8.10 (1H, d, *J* = 8.58 Hz), 7.53 (1H, s). ^{13}C NMR (150 MHz, $CDCl_3$): 122.57 (CH), 124.46 (CH), 126.90 (CH), 127.89 (CH), 128.50 (C), 128.69 (CH), 129.31 (2CH), 129.56 (CH), 129.68 (2CH), 130.15 (CH), 132.61 (CH), 133.51 (C), 135.43 (C), 135.62 (C), 136.47 (C), 143.28 (CH), 189.93 (C). For $C_{19}H_{13}ClO$: MS (FAB) $[M+H]^+$: m/z = 293.0.

3-(4-Chlorophenyl)-1-(2-methoxynaphthalen-1-yl)prop-2-en-1-one (A2) [34, 35]: Yield: 90%. ^{1}H NMR (600 MHz, $CDCl_3$): 3.82 (3H, s), 7.09 (1H, d, *J* = 17.22 Hz), 7.27 (1H, d, *J* = 16.32 Hz), 7.31-7.34 (3H, m), 7.37 (1H, t, *J* = 7.68 Hz), 7.41-7.46 (3H, s), 7.69 (1H, d, *J* = 7.68 Hz), 7.83 (1H, d, *J* = 8.16 Hz), 7.94 (1H, d, *J* = 9.06 Hz). ^{13}C NMR (150 MHz, $CDCl_3$): 56.79 (CH_3), 113.28 (CH), 124.06 (CH), 124.22 (CH), 127.60 (CH), 128.16 (CH), 128.89 (CH), 129.19 (2CH), 129.26 (CH), 129.65 (2CH), 131.48 (2C), 131.52 (C), 133.24 (C), 136.48 (C), 143.90 (CH), 154.32 (C), 196.98 (C). For $C_{20}H_{15}ClO_2$: MS (FAB^+) $[M+H]^+$: m/z = 322.9.

5-(4-Chlorophenyl)-3-(naphthalen-2-yl)-4,5-dihydro-1*H*-pyrazole-1-carbothioamide (B1) [37]: Yield: 86%. ^{1}H NMR (600 MHz, $CDCl_3$): 3.31 (1H, dd, J_{AB} = 17.52 Hz, J_{AX} = 3.84 Hz), 3.94 (1H, dd, J_{BA} = 17.40 Hz, J_{BX} = 11.40 Hz), 6.06 (1H, dd, J_{BX} = 11.52 Hz, J_{AX} = 3.72 Hz), 6.19 (1H, bs), 7.20 (3H, d, *J* = 8.82 Hz), 7.30 (2H, d, *J* = 8.82 Hz), 7.52-7.57 (2H, m), 7.83-7.88 (3H, m), 7.95 (1H, s), 8.00 (1H, d, *J* = 8.79 Hz). ^{13}C NMR (150 MHz, $CDCl_3$): 43.11 (CH_2), 63.36 (CH), 123.10 (CH), 127.05 (2CH), 127.07 (CH), 127.80 (CH), 127.95 (CH), 128.02 (CH), 128.08 (CH), 128.55

(CH), 128.77 (C), 129.14 (2CH), 132.96 (C), 133.48 (C), 134.51 (C), 140.40 (C), 155.89 (C), 176.83 (C). For $C_{20}H_{16}ClN_3S$: MS (FAB) $[M+H]^+$: m/z = 365.9.

5-(4-Chlorophenyl)-3-(2-methoxynaphthalen-1-yl)-4,5-dihydro-1*H*-pyrazole-1-carbothioamide (B2): Yield: 86%. ^{1}H NMR (600 MHz, $CDCl_3$): 3.24 (1H, dd, J_{AB} = 18.12 Hz, J_{AX} = 3.24 Hz), 3.92 (3H, s), 3.95 (1H, dd, J_{BA} = 20.64 Hz, J_{BX} = 9.12 Hz), 6.04 (1H, dd, J_{BX} = 11.52 Hz, J_{AX} = 3.00 Hz), 7.03 (1H, bs), 7.30 (1H, d, *J* = 9.54 Hz), 7.36 (4H, s), 7.41 (1H, t, *J* = 6.54 Hz), 7.53 (1H, t, *J* = 8.04 Hz), 7.83 (1H, d, *J* = 7.50 Hz), 7.94 (1H, d, *J* = 9.00 Hz), 7.99 (1H, d, *J* = 8.28 Hz). ^{13}C NMR (150 MHz, $CDCl_3$): 47.13 (CH_2), 56.37 (CH_3), 62.74 (CH), 112.66 (C), 123.71 (CH), 124.36 (CH), 127.49 (3CH), 127.99 (CH), 128.45 (CH), 128.98 (3CH), 132.03 (C), 132.10 (C), 133.40 (C), 140.53 (C), 156.05 (C), 156.49 (C), 177.09 (C). HRMS (FAB) calcd. for $C_{21}H_{18}ClN_3OS$ $[M+H]^+$: m/z = 396.0937, found: 396.0926.

1-(4-(4-(Cyanophenyl)thiazole-2-yl)-3-(naphthalen-2-yl)-5-(4-chlorophenyl)-2-pyrazoline (BTT-1): Yield: 92%. ^{1}H NMR (600 MHz, DMSO-*d*$_6$): 3.53 (1H, dd, J_{AB} = 17.64 Hz, J_{AX} = 6.48 Hz), 4.20 (1H, dd, J_{BA} = 17.70 Hz, J_{BX} = 11.76 Hz), 5.78 (1H, dd, J_{BX} = 12.48 Hz, J_{AX} = 6.60 Hz), 7.45 (2H, d, *J* = 9.18 Hz), 7.49 (2H, d, *J* = 9.24 Hz), 7.57-7.60 (2H, m), 7.68 (1H, s), 7.83 (2H, d, *J* = 7.20 Hz), 7.90 (2H, d, *J* = 8.52 Hz), 7.97-8.02 (3H, m), 8.06 (1H, d, *J* =8.55 Hz), 8.20 (1H, s). ^{13}C NMR (150 MHz, DMSO-*d*$_6$): 42.85 (CH_2), 63.51 (CH), 108.49 (C), 119.13 (C, C≡N), 123.08 (CH), 126.05 (2CH), 126.90 (CH), 127.18 (CH), 127.28 (CH), 127.76 (CH), 128.32 (CH), 128.37 (C), 128.43 (2CH), 128.60 (2CH), 128.64 (2CH), 132.12 (C), 132.64 (2CH), 132.77 (C), 135.67 (C), 138.46 (C), 140.57 (C), 148.74 (C), 153.45 (C), 164.46 (C). HRMS (FAB) calcd. for $C_{29}H_{19}ClN_4S$ $[M+H]^+$:m/z = 490.0999, found: 490.1019.

1-(4-(4-(Nitrophenyl)thiazole-2-yl)-3-(naphthalen-2-yl)-5-(4-chlorophenyl)-2-pyrazoline (BTT-2): Yield: 90%. ^{1}H NMR (600 MHz, DMSO-d_6): 3.55 (1H, dd, J_{AB} = 17.76 Hz, J_{AX} = 6.60 Hz), 4.20 (1H, dd, J_{BA} = 17.10 Hz, J_{BX} = 12.48 Hz), 5.79 (1H, dd, J_{BX} = 10.50 Hz, J_{AX} = 6.60 Hz), 7.46 (2H, d, J = 9.84 Hz), 7.51 (2H, d, J = 8.58 Hz), 7.58-7.61 (2H, m), 7.75 (1H, s), 7.97-8.02 (5H, m), 8.06 (1H, d, J = 8.70 Hz), 8.21 (1H, s), 8.24 (2H, d, J = 9.54 Hz). ^{13}C NMR (150 MHz, DMSO-d_6): 42.66 (CH_2), 63.68 (CH), 109.37 (CH), 122.99 (CH), 124.06 (2CH), 126.32 (2CH), 126.90 (CH), 127.18 (CH), 127.29 (C), 127.77 (CH), 128.30 (CH), 128.39 (CH), 128.47 (CH), 128.63 (2CH), 128.77 (2CH), 132.18 (C), 132.79 (C), 133.55 (C), 140.37 (C), 140.46 (C), 146.28 (C), 148.44 (C), 153.53 (C), 164.49 (C). HRMS (FAB) calcd. for $C_{28}H_{19}ClN_4O_2S$ $[M+H]^+$:m/z = 510.0926 found: 510.0917.

1-(4-(4-(Fluorophenyl)thiazole-2-yl)-3-(naphthalen-2-yl)-5-(4-chlorophenyl)-2-pyrazoline (BTT-3) [42]: Yield: 88%. ^{1}H NMR (600 MHz, DMSO-d_6): 3.51 (1H, dd, J_{AB} = 17.76 Hz, J_{AX} = 6.60 Hz), 4.19 (1H, dd, J_{BA} = 18.06 Hz, J_{BX} = 12.12 Hz), 5.79 (1H, dd, J_{BX} = 12.18 Hz, J_{AX} = 6.30 Hz), 7.20 (2H, d, J = 8.58 Hz), 7.35 (1H, s), 7.44 (2H, d, J = 8.58 Hz), 7.48 (2H, d, J = 8.22 Hz), 7.57-7.60 (2H, m), 7.75-7.77 (2H, m), 7.96-8.02 (3H, m), 8.06 (1H, d, J = 8.61 Hz). 8.20 (1H, s). ^{13}C NMR (150 MHz, DMSO-d_6): 42.95 (CH_2), 64.02 (CH), 104.54 (CH), 115.31 (CH), 115.44 (CH), 123.18 (CH), 126.87 (CH), 127.05 (CH), 127.24 (CH), 127.41 (CH), 127.48 (CH), 127.75 (C), 128.34 (CH), 128.42 (2CH), 128.58 (2CH), 128.62 (2CH), 131.01 (C), 132.05 (C), 132.79 (C), 133.48 (C), 140.75 (C), 149.42 (C), 153.04 (C), 160.79 (C), 164.29 (C). HRMS (FAB) calcd. for $C_{28}H_{19}ClFN_3S$ $[M+H]^+$:m/z = 483.0956 found: 483.0972.

1-(4-(4-(Chlorophenyl)thiazole-2-yl)-3-(naphthalen-2-yl)-5-(4-chlorophenyl)-2-pyrazoline (BTT-4) [42]: Yield: 84%. ^{1}H NMR (600 MHz, DMSO-d_6): 3.52 (1H, dd,

J_{AB} = 18.06 Hz, J_{AX} = 6.60 Hz), 4.19 (1H, dd, J_{BA} = 17.10 Hz, J_{BX} = 11.22 Hz), 5.77 (1H, dd, J_{BX} = 11.82 Hz, J_{AX} = 6.54 Hz), 7.44 (5H, t, J = 8.52 Hz), 7.48 (2H, d, J = 8.58 Hz), 7.58-7.59 (2H, m), 7.74 (2H, d, J = 8.22 Hz), 7.96-8.02 (3H, m), 8.06 (1H, d, J = 8.37 Hz). 8.20 (1H, s). ^{13}C NMR (150 MHz, DMSO-d_6): 43.30 (CH_2), 63.69 (CH), 105.92 (CH), 123.14 (CH), 126.87 (CH), 127.07 (CH), 127.19 (2CH), 127.26 (C), 128.36 (CH), 128.39 (CH), 128.41 (2CH), 128.57 (3CH), 128.66 (3CH), 131.98 (C), 132.09 (C), 132.79 (C), 133.26 (C), 133.47 (C), 140.66 (C), 149.24 (C), 153.15 (C), 164.35 (C). HRMS (FAB) calcd. for $C_{28}H_{19}Cl_2N_3S$ $[M+H]^+$:m/z = 499.0660 found: 499.0667.

1-(4-(4-(Bromophenyl)thiazole-2-yl)-3-(naphthalen-2-yl)-5-(4-chlorophenyl)-2-pyrazoline (BTT-5): Yield: 88%. ^{1}H NMR (600 MHz, DMSO-d_6): 3.52 (1H, dd, J_{AB} = 17.76 Hz, J_{AX} = 6.42 Hz), 4.19 (1H, dd, J_{BA} = 17.58 Hz, J_{BX} = 11.76 Hz), 5.77 (1H, dd, J_{BX} = 11.88 Hz, J_{AX} = 6.42 Hz), 7.44 (3H, d, J = 6.84 Hz), 7.48 (2H, d, J = 9.42 Hz), 7.56-7.59 (4H, m), 7.68 (2H, d, J = 8.76 Hz), 7.96-8.01 (3H, m), 8.06 (1H, d, J = 8.67 Hz), 8.19 (1H, s). ^{13}C NMR (150 MHz, DMSO-d_6): 42.93 (CH_2), 63.71 (CH), 105.50 (CH), 120.70 (C), 123.07 (CH), 126.89 (CH), 127.09 (CH), 127.22 (2CH), 127.46 (2CH), 127.76 (CH), 128.34 (CH), 128.38 (CH), 128.40 (CH), 128.57 (2CH), 128.66 (2CH), 131.47 (C), 132.07 (C), 132.79 (C), 133.48 (C), 133.59 (C), 140.66 (C), 149.28 (C), 153.15 (C), 164.35 (C). HRMS (FAB$^+$) calcd. for $C_{28}H_{19}BrClN_3S$ $[M+H]^+$:m/z = 543.0135 found: 543.0172.

1-(4-(4-(Methylphenyl)thiazole-2-yl)-3-(naphthalen-2-yl)-5-(4-chlorophenyl)-2-pyrazoline (BTT-6): Yield: 86%. ^{1}H NMR (600 MHz, DMSO-d_6): 2.29 (3H, s), 3.50 (1H, dd, J_{AB} = 17.52 Hz, J_{AX} = 6.84 Hz), 4.18 (1H, dd, J_{BA} = 17.10 Hz, J_{BX} = 11.52 Hz), 5.76 (1H, dd, J_{BX} = 12.84 Hz, J_{AX} = 6.42 Hz), 7.17 (2H, d, J = 8.16 Hz), 7.28 (1H, s), 7.44 (2H, d, J = 10.32 Hz), 7.48 (2H, d, J = 9.42 Hz), 7.57-7.59 (2H, m), 7.61 (2H, d, J

= 7.74 Hz), 7.96-8.01 (3H, m), 8.06 (1H, d, J = 8.67 Hz), 8.19 (1H, s). ^{13}C NMR (150 MHz, DMSO-d_6): 20.60 (CH_3), 42.57 (CH_2), 63.64 (CH), 103.62 (CH), 123.04 (CH), 125.44 (2CH), 126.85 (CH), 126.97 (CH), 127.22 (CH), 127.73 (C), 128.34 (CH), 128.40 (CH), 128.47 (CH), 128.53 (2CH), 128.67 (2CH), 129.07 (2CH), 131.78 (C), 132.05 (C), 132.82 (C), 133.45 (C), 136.84 (C), 140.82 (C), 150.58 (C), 152.86 (C), 164.13 (C). HRMS (FAB$^+$) calcd. for $C_{29}H_{22}ClN_3S$ $[M+H]^+$:m/z = 479.1207 found: 479.1223.

1-(4-(4-(Methoxyphenyl)thiazole-2-yl)-3-(naphthalen-2-yl)-5-(4-chlorophenyl)-2-pyrazoline (BTT-7) [42]: Yield: 88%. ^1H NMR (600 MHz, DMSO-d_6): 3.50 (1H, dd, J_{AB} = 17.82 Hz, J_{AX} = 6.78 Hz), 3.77 (3H, s), 4.18 (1H, dd, J_{BA} = 17.40 Hz, J_{BX} = 11.76 Hz), 5.76 (1H, dd, J_{BX} = 11.94 Hz, J_{AX} = 6.60 Hz), 6.93 (2H, d, J = 9.48 Hz), 7.19 (1H, s), 7.45 (2H, d, J = 8.58 Hz), 7.48 (2H, d, J = 8.04 Hz), 7.58-7.59 (2H, m), 7.65 (2H, d, J = 8.28 Hz), 7.96-8.01 (3H, m), 8.06 (1H, d, J = 8.79 Hz), 8.19 (1H, s). ^{13}C NMR (150 MHz, DMSO-d_6): 42.93 (CH_2), 55.03 (CH_3), 63.80 (CH), 102.11 (CH), 113.97 (2CH), 123.03 (CH), 126.81 (2CH), 126.86 (C), 126.97 (CH), 127.19 (C), 127. 31 (CH), 127.74 (CH), 128.34 (CH), 128.42 (CH), 128.48 (CH), 128.55 (2CH), 128.67 (2CH), 132.06 (C), 132.77 (C), 133.48 (C), 140.89 (C), 150.35 (C), 152.78 (C), 158.86 (C), 164.17 (C). HRMS (FAB$^+$) calcd. for $C_{29}H_{22}ClN_3OS$ $[M+H]^+$:m/z = 495.1172 found: 495.1172.

1-(4-(4-(Trifluoromethylphenyl)thiazole-2-yl)-3-(naphthalen-2-yl)-5-(4-chlorophenyl)-2-pyrazoline (BTT-8): Yield: 84%. ^1H NMR (600 MHz, DMSO-d_6): 3.54 (1H, dd, J_{AB} = 17.34 Hz, J_{AX} = 6.30 Hz), 4.20 (1H, dd, J_{BA} = 17.70 Hz, J_{BX} = 11.76 Hz), 5.79 (1H, dd, J_{BX} = 12.00 Hz, J_{AX} = 6.60 Hz), 7.46 (2H, d, J = 8.40 Hz), 7.50 (2H, d, J = 8.64 Hz), 7.59-7.62 (3H, m), 7.74 (2H, d, J = 8.28 Hz), 7.94 (2H, d, J = 8.16 Hz), 7.97-8.02 (3H, m), 8.07 (1H, d, J = 8.55 Hz), 8.21 (1H, s). ^{13}C NMR (150 MHz, DMSO-

d_6): 43.13 (CH_2), 63.61 (CH), 107.18 (CH), 123.05 (CH), 125.53 (C), 126.01 (2CH), 126.89 (CH), 127.12 (CH), 127.24 (CH), 127.76 (C), 128.37 (2CH), 128.45 (2CH), 128.48 (CH), 128.59 (2CH), 128.75 (2CH), 132.16 (C), 132.78 (2C), 133.50 (C), 138.09 (C), 140.58 (C), 148.94 (C), 153.34 (C), 164.44 (C). HRMS (FAB^+) calcd. for $C_{29}H_{19}ClF_3N_3S$ $[M+H]^+$:m/z = 533.0923 found: 533.0940.

1-(4-(4-(Trifluoromethoxyphenyl)thiazole-2-yl)-3-(naphthalen-2-yl)-5-(4-chlorophenyl)-2-pyrazoline (BTT-9): Yield: 86%.^{1}H NMR (600 MHz, DMSO-d_6): 3.53 (1H, dd, J_{AB} = 17.28 Hz, J_{AX} = 6.00 Hz), 4.20 (1H, dd, J_{BA} = 17.82 Hz, J_{BX} = 12.30 Hz), 5.77 (1H, dd, J_{BX} = 12.30 Hz, J_{AX} = 7.02 Hz), 7.37 (2H, d, J = 8.28 Hz), 7.45 (3H, d, J = 7.14 Hz), 7.49 (2H, d, J = 8.28 Hz), 7.58-7.60 (2H, m), 7.84 (2H, d, J = 9.24 Hz), 7.97-8.02 (3H, m), 8.07 (1H, d, J = 8.28 Hz), 8.20 (1H, s). ^{13}C NMR (150 MHz, DMSO-d_6): 43.12 (CH_2), 63.82 (CH), 105.70 (CH), 121.16 (2CH), 123.01 (CH), 126.88 (CH), 127.09 (CH), 127.20 (2CH), 127.74 (CH), 128.35 (CH), 128.38 (CH), 128.41 (CH), 128.57 (2CH), 128.65 (2CH), 132.10 (2C), 132.78 (2C), 133.50 (C), 133.67 (C), 140.64 (C), 147.56 (C), 149.01 (C), 153.17 (C), 164.40 (C). HRMS (FAB^+) calcd. for $C_{29}H_{19}ClF_3N_3OS$ $[M+H]^+$:m/z = 549.0903 found: 549.0889.

1-(4-(4-(Methylsulfonylphenyl)thiazole-2-yl)-3-(naphthalen-2-yl)-5-(4-chlorophenyl)-2-pyrazoline (BTT-10): Yield: 82%. ^{1}H NMR (600 MHz, DMSO-d_6): 3.22 (3H, s), 3.50 (1H, dd, J_{AB} = 17.82 Hz, J_{AX} = 6.78 Hz), 4.21 (1H, dd, J_{BA} = 17.52 Hz, J_{BX} = 11.76 Hz), 5.79 (1H, dd, J_{BX} = 12.06 Hz, J_{AX} = 7.02 Hz), 7.47 (2H, d, J = 8.76 Hz), 7.51 (2H, d, J = 7.98 Hz), 7.59-7.60 (2H, m), 7.67 (1H, s), 7.92 (2H, d, J = 8.52 Hz), 7.98 (3H, d, J = 8.28 Hz), 7.99-8.03 (2H, m), 8.07 (1H, d, J = 8.52 Hz), 8.22 (1H, s). ^{13}C NMR (150 MHz, DMSO-d_6): 43.00 (CH_3), 43.63 (CH_2), 63.72 (CH), 108.08 (CH), 123.03 (CH), 126.03 (2CH), 126.89 (CH), 127.13 (CH), 127.25 (CH), 127.42 (2CH),

127.75 (C), 128.34 (CH), 128.37 (CH), 128.43 (CH), 128.58 (2CH), 128.79 (2CH), 132.14 (C), 132.77 (C), 133.52 (C), 138.88 (C), 139.25 (C), 140.52 (C), 148.81 (C), 153.36 (C), 164.40 (C). HRMS (FAB^+) calcd. for $C_{29}H_{22}ClN_3O_2S_2$ $[M+H]^+$:m/z = 543.0842 found: 543.0842.

1-(4-(4-(Cyanophenyl)thiazole-2-yl)-3-(2-methoxynaphthalen-1-yl)-5-(4-chlorophenyl)-2-pyrazoline (BTP-1): Yield: 83%.^{1}H NMR (600 MHz, $CDCl_3$): 3.46 (1H, dd, J_{AB} = 18.00 Hz, J_{AX} = 6.72 Hz), 3.96 (3H, s), 3.99 (1H, dd, J_{BA} = 18.18 Hz, J_{BX} = 12.30 Hz), 5.67 (1H, dd, J_{BX} = 12.03 Hz, J_{AX} = 7.02 Hz), 6.97 (1H, s), 7.31 (1H, d, J = 11.04 Hz), 7.37-7.42 (3H, m), 7.51-7.56 (3H, m), 7.62 (2H, d, J = 7.92 Hz), 7.78 (2H, d, J = 7.92 Hz), 7.84 (1H, d, J = 9.60 Hz), 7.93 (1H, d, J = 11.40 Hz), 8.24 (1H, d, J = 8.52 Hz). ^{13}C NMR (150 MHz, $CDCl_3$): 47.44 (CH_2), 56.72 (CH_3), 63.82 (CH), 106.79 (CH), 110.96 (C), 112.66 (CH), 114.36 (CH), 119.46 (C, C≡N), 124.25 (CH), 124.33 (CH), 126.24 (2CH), 128.31 (3CH), 128.90 (2CH), 129.17 (CH), 131.71 (2C), 132.42 (2CH), 132.47 (C), 133.52 (C), 139.07 (C), 140.40 (C), 149.71 (C), 152.05 (C), 156.08 (C), 165.72 (C). HRMS (FAB^+) calcd. for $C_{30}H_{21}ClN_4OS$ $[M+H]^+$:m/z = 520.1125, found: 520.1104.

1-(4-(4-(Nitrophenyl)thiazole-2-yl)-3-(2-methoxynaphthalen-1-yl)-5-(4-chlorophenyl)-2-pyrazoline (BTP-2): Yield: 84%.^{1}H NMR (600 MHz, $CDCl_3$): 3.47 (1H, dd, J_{AB} = 17.46 Hz, J_{AX} = 6.48 Hz), 3.97 (3H, s), 3.99 (1H, dd, J_{BA} = 18.06 Hz, J_{BX} = 12.24 Hz), 5.68 (1H, dd, J_{BX} = 11.64 Hz, J_{AX} = 7.08 Hz), 7.03 (1H, s), 7.31 (1H, d, J = 9.06 Hz), 7.38-7.42 (3H, m), 7.52-7.56 (3H, m), 7.81-7.84 (3H, m), 7.93 (1H, d, J = 8.01 Hz), 8.20 (2H, d, J = 9.06 Hz), 8.24 (1H, d, J = 11.28 Hz). ^{13}C NMR (150 MHz, $CDCl_3$): 47.60 (CH_2), 56.56 (CH_3), 63.98 (CH), 107.56 (CH), 112.74 (C), 114.28 (CH), 123.99 (2CH), 124.19 (CH), 124.29 (CH), 126.26 (2CH), 127.73 (CH), 128.32 (3CH), 128.90

(2CH), 129.14 (C), 131.65 (CH), 132.42 (C), 133.66 (C), 140.36 (C), 140.88 (C), 146.73 (C), 149.34 (C), 152.20 (C), 156.01 (C), 165.80 (C). HRMS (FAB$^+$) calcd. for $C_{29}H_{21}N_4ClS$ $[M+H]^+$:m/z = 540.1023, found: 540.1022.

1-(4-(4-(Fluorophenyl)thiazole-2-yl)-3-(2-methoxynaphthalen-1-yl)-5-(4-chlorophenyl)-2-pyrazoline (BTP-3): Yield: 85%.^{1}H NMR (600 MHz, $CDCl_3$): 3.44 (1H, dd, J_{AB} = 17.76 Hz, J_{AX} = 6.48 Hz), 3.96 (3H, s), 3.98 (1H, dd, J_{BA} = 18.06 Hz, J_{BX} = 11.82 Hz), 5.69 (1H, bs), 6.74 (1H, s), 7.03 (2H, t, *J* = 9.00 Hz), 7.31 (1H, d, *J* = 9.00 Hz), 7.37-7.42 (3H, m), 7.52-7.55 (3H, m), 7.65-7.68 (2H, m), 7.83 (1H, d, *J* = 7.50 Hz), 7.93 (1H, d, *J* = 9.54 Hz), 8.25 (1H, d, *J* = 8.52 Hz). ^{13}C NMR (150 MHz, $CDCl_3$): 47.44 (CH_2), 56.56 (CH_3), 63.82 (CH), 102.96 (CH), 112.77 (C), 115.21 (CH), 115.42 (CH), 124.11 (CH), 127.55 (2CH, d, *J* = 10.14 Hz), 127.71 (CH), 128.28 (2CH), 128.39 (3CH), 128.80 (3CH), 129.16 (C), 131.56 (C), 132.54 (C), 133.41 (C), 140.58 (C), 156.05 (2C), 161.65 (C), 163.35 (C), 165.67 (C). HRMS (FAB$^+$) calcd. for $C_{29}H_{21}ClFN_3OS$ $[M+H]^+$:m/z = 513.1071, found: 513.1078.

1-(4-(4-(Chlorophenyl)thiazole-2-yl)-3-(2-methoxynaphthalen-1-yl)-5-(4-chlorophenyl)-2-pyrazoline (BTP-4): Yield: 88%. ^{1}H NMR (600 MHz, $CDCl_3$): 3.45 (1H, dd, J_{AB} = 18.06 Hz, J_{AX} = 6.54 Hz), 3.96 (3H, s), 3.98 (1H, dd, J_{BA} = 18.00 Hz, J_{BX} = 12.00 Hz), 5.68 (1H, bs), 6.80 (1H, s), 7.30 (3H, d, *J* = 9.06 Hz), 7.36-7.41 (3H, m), 7.52-7.56 (3H, m), 7.63 (2H, d, *J* = 7.02 Hz), 7.83 (1H, d, *J* = 7.02 Hz), 7.93 (1H, d, *J* = 10.02 Hz), 8.25 (1H, d, *J* = 8.04 Hz). ^{13}C NMR (150 MHz, $CDCl_3$): 47.42 (CH_2), 56.39 (CH_3), 63.92 (CH), 103.81 (CH), 112.76 (C), 124.13 (CH), 124.38 (CH), 127.17 (2CH), 127.76 (C), 128.27 (CH), 128.42 (3CH), 128.61 (3CH), 128.82 (3CH), 129.18 (C), 131.59 (C), 132.48 (C), 133.44 (C), 140.53 (C), 156.05 (2C), 158.21 (C), 165.65 (C). HRMS (FAB$^+$) calcd. for $C_{29}H_{21}Cl_2N_3OS$ $[M+H]^+$:m/z = 529.0782, found: 529.0782.

1-(4-(4-(Bromophenyl)thiazole-2-yl)-3-(2-methoxynaphthalen-1-yl)-5-(4-chlorophenyl)-2-pyrazoline (BTP-5): Yield: 86%. ^{1}H NMR (600 MHz, $CDCl_3$): 3.44 (1H, dd, J_{AB} = 17.70 Hz, J_{AX} = 6.66 Hz), 3.95 (3H, s), 3.97 (1H, dd, J_{BA} = 18.30 Hz, J_{BX} = 11.88 Hz), 5.67 (1H, dd, J_{BX} = 12.18 Hz, J_{AX} = 6.66 Hz), 6.81 (1H, s), 7.30 (1H, d, J = 9.18 Hz), 7.37 (2H, d, J = 8.76 Hz), 7.39-7.41 (1H, m), 7.45 (2H, d, J = 9.06 Hz), 7.51-7.57 (5H, m), 7.82 (1H, d, J = 8.52 Hz), 7.92 (1H, d, J = 9.18 Hz), 8.25 (1H, d, J = 8.76 Hz). ^{13}C NMR (150 MHz, $CDCl_3$): 47.19 (CH_2), 56.13 (CH_3), 64.15 (CH), 104.09 (CH), 112.58 (C), 121.37 (C), 124.15 (CH), 127.47 (3CH), 127.75 (2CH), 128.28 (2CH), 128.80 (3CH), 129.16 (C), 131.58 (3CH), 132.52 (C), 133.46 (C), 134.00 (C), 140.55 (C), 150.45 (C), 151.68 (C), 156.05 (C), 165.63 (C). HRMS (FAB^+) calcd. for $C_{29}H_{21}BrClN_3OS$ $[M+H]^+$:m/z = 573.0266, found: 575.0257.

1-(4-(4-(Methylphenyl)thiazole-2-yl)-3-(2-methoxynaphthalen-1-yl)-5-(4-chlorophenyl)-2-pyrazoline (BTP-6): Yield: 87%. ^{1}H NMR (600 MHz, $CDCl_3$): 2.34 (3H, s), 3.44 (1H, dd, J_{AB} = 17.82 Hz, J_{AX} = 6.66 Hz), 3.95 (3H, s), 3.98 (1H, bs), 5.69 (1H, dd, J_{BX} = 11.52 Hz, J_{AX} = 6.54 Hz), 6.75 (1H, s), 7.15 (2H, d, J = 10.02 Hz), 7.29 (1H, d, J = 10.02 Hz), 7.36 (2H, d, J = 8.04 Hz), 7.40 (1H, d, J = 7.32 Hz), 7.54 (3H, d, J = 7.98 Hz), 7.59 (2H, d, J = 8.04 Hz), 7.82 (1H, d, J = 7.02 Hz), 7.92 (1H, d, J = 8.52 Hz), 8.26 (1H, d, J = 8.52 Hz). ^{13}C NMR (150 MHz, $CDCl_3$): 21.48 (CH_3), 47.43 (CH_2), 56.41 (CH_3), 64.29 (CH), 102.77 (CH), 112.81 (C), 124.13 (CH), 124.44 (CH), 125.83 (2CH), 127.73 (CH), 128.23 (CH), 128.48 (3CH), 128.74 (2CH), 129.14 (3CH), 131.49 (C), 132.40 (C), 132.57 (C), 133.33 (C), 137.21 (C), 140.71 (C), 151.29 (C), 151.54 (C), 154.96 (C), 165.45 (C). HRMS (FAB^+) calcd. for $C_{30}H_{24}ClN_3OS$ $[M+H]^+$:m/z = 509.1328, found: 509.1329.

1-(4-(4-(Methoxyphenyl)thiazole-2-yl)-3-(2-methoxynaphthalen-1-yl)-5-(4-chlorophenyl)-2-pyrazoline (BTP-7): Yield: 85%. ^{1}H NMR (600 MHz, $CDCl_3$): 3.43 (1H, dd, J_{AB} = 18.18 Hz, J_{AX} = 6.90 Hz), 3.82 (3H, s), 3.95 (3H, s), 3.97 (1H, d, J_{BX} = 11.76 Hz), 5.69 (1H, dd, J_{BX} = 11.28 Hz, J_{AX} = 7.02 Hz), 6.67 (1H, s), 6.88 (2H, d, *J* = 9.54 Hz), 7.30 (1H, d, *J* = 9.54 Hz), 7.37 (2H, d, *J* = 8.52 Hz), 7.40 (1H, d, *J* = 7.44 Hz), 7.53-7.55 (3H, m), 7.64 (2H, d, *J* = 9.30 Hz), 7.82 (1H, d, *J* = 8.04 Hz), 7.92 (1H, d, *J* = 9.03 Hz), 8.26 (1H, d, *J* = 8.52 Hz). ^{13}C NMR (150 MHz, $CDCl_3$): 47.48 (CH_2), 55.40 (CH_3), 56.37 (CH_3), 64.06 (CH), 101.69 (CH), 112.81 (C), 113.86 (2CH), 124.13 (CH), 124.44 (CH), 127.15 (2CH), 127.69 (2CH), 128.25 (2CH), 128.44 (2CH), 128.77 (2CH), 129.19 (C), 131.59 (C), 132.59 (C), 133.41 (C), 140.82 (C), 151.37 (C), 156.01 (2C), 159.17 (C), 165.55 (C). HRMS (FAB^+) calcd. for $C_{30}H_{24}ClN_3O_2S$ $[M+H]^+$:m/z = 525.1281, found: 525.1278.

1-(4-(4-(Trifluoromethylphenyl)thiazole-2-yl)-3-(2-methoxynaphthalen-1-yl)-5-(4-chlorophenyl)-2-pyrazoline (BTP-8): Yield: 84%. ^{1}H NMR (600 MHz, $CDCl_3$): 3.45 (1H, dd, J_{AB} = 18.06 Hz, J_{AX} = 6.78 Hz), 3.96 (3H, s), 3.99 (1H, dd, J_{BA} = 17.94 Hz, J_{BX} = 11.76 Hz), 5.68 (1H, dd, J_{BX} = 11.76 Hz, J_{AX} = 6.54 Hz), 6.92 (1H, s), 7.31 (1H, d, *J* = 8.76 Hz), 7.38 (2H, d, *J* = 8.28 Hz), 7.41 (1H, t, *J* = 7.32 Hz), 7.52-7.56 (3H, m), 7.59 (2H, d, *J* = 8.04 Hz), 7.79 (2H, d, *J* = 8.04 Hz), 7.83 (1H, d, *J* = 8.04 Hz), 7.93 (1H, d, *J* = 9.06 Hz), 8.25 (1H, d, *J* = 7.98 Hz). ^{13}C NMR (150 MHz, $CDCl_3$): 47.41 (CH_2), 56.55 (CH_3), 63.99 (CH), 105.51 (CH), 112.80 (C), 124.15 (CH), 124.35 (CH), 125.47 (C, CF_3), 125.99 (2CH), 127.75 (2CH), 128.31 (2CH), 128.39 (3CH), 128.84 (3CH), 129.17 (C), 131.61 (C), 132.49 (C), 133.49 (C), 138.30 (C), 140.48 (C), 150.15 (C), 151.86 (C), 156.06 (C), 165.72 (C). HRMS (FAB^+) calcd. for $C_{30}H_{21}ClF_3N_3OS$ $[M+H]^+$:m/z = 563.1066, found: 563.1046.

1-(4-(4-(Trifluoromethoxyphenyl)thiazole-2-yl)-3-(2-methoxynaphthalen-1-yl)-5-(4-chlorophenyl)-2-pyrazoline (BTP-9): Yield: 88%. ^{1}H NMR (600 MHz, $CDCl_3$): 3.44 (1H, dd, J_{AB} = 17.64 Hz, J_{AX} = 6.30 Hz), 3.96 (3H, s), 3.98 (1H, dd, J_{BA} = 17.88 Hz, J_{BX} = 11.82 Hz), 5.67 (1H, dd, J_{BX} = 11.58 Hz, J_{AX} = 6.54 Hz), 6.80 (1H, s), 7.18 (2H, d, J = 8.04 Hz), 7.30 (1H, d, J = 9.06 Hz), 7.37 (2H, d, J = 8.52 Hz), 7.40 (1H, t, J = 7.56 Hz), 7.51-7.56 (3H, m), 7.70 (2H, d, J = 8.82 Hz), 7.83 (1H, d, J = 7.56 Hz), 7.92 (1H, d, J = 8.82 Hz), 8.25 (1H, d, J = 8.52 Hz). ^{13}C NMR (150 MHz, $CDCl_3$): 47.49 (CH_2), 56.40 (CH_3), 63.84 (CH), 104.00 (CH), 112.76 (C), 120.97 (2CH), 124.11 (CH), 124.38 (CH), 127.21 (2CH), 127.73 (2CH), 128.29 (2CH), 128.36 (2CH), 128.82 (2CH), 129.16 (C), 131.57 (C), 132.53 (C), 133.43 (C), 133.83 (C), 140.56 (C), 148.48 (C), 150.20 (C), 151.69 (C), 156.06 (C), 165.69 (C). HRMS (FAB$^+$) calcd. for $C_{30}H_{21}ClF_3N_3O_2S$ [M+H]$^+$:m/z = 579.1000, found: 579.0995.

1-(4-(4-(Methylsulfonylphenyl)thiazole-2-yl)-3-(2-methoxynaphthalen-1-yl)-5-(4-chlorophenyl)-2-pyrazoline (BTP-10): Yield: 80%. ^{1}H NMR (600 MHz, $CDCl_3$): 3.22 (3H, s), 3.56 (1H, dd, J_{AB} = 17.76 Hz, J_{AX} = 6.72 Hz), 3.99 (3H, s), 4.00 (1H, dd, J_{BA} = 18.00 Hz, J_{BX} = 11.76 Hz), 5.68 (1H, dd, J_{BX} = 11.94 Hz, J_{AX} = 6.78 Hz), 6.99 (1H, s), 7.31 (1H, d, J = 9.06 Hz), 7.38 (1H, d, J = 8.04 Hz), 7.41 (2H, t, J = 7.50 Hz), 7.52-7.56 (3H, m), 7.83 (1H, d, J = 8.52 Hz), 7.86 (2H, d, J = 8.52 Hz), 7.90 (2H, d, J = 8.76 Hz), 7.94 (1H, d, J = 8.82 Hz), 8.25 (1H, d, J = 8.82 Hz). ^{13}C NMR (150 MHz, $CDCl_3$): 44.78 (CH_3), 47.26 (CH_2), 56.71 (CH_3), 63.84 (CH), 106.91 (CH), 112.80 (C), 124.16 (CH), 124.29 (CH), 126.49 (2CH), 127.72 (2CH), 127.76 (CH), 128.32 (CH), 128.37 (2CH), 128.85 (3CH), 129.16 (CH), 131.66 (C), 132.46 (C), 133.54 (C), 138.78 (C), 140.06 (C), 140.38 (C), 149.57 (C), 152.07 (C), 156.09 (C), 165.76 (C). HRMS (FAB$^+$) calcd. for $C_{30}H_{24}ClN_3O_3S_2$ [M+H]$^+$:m/z = 573.0938, found: 573.0948.

Supplementary Figures

Figure S1: ^{1}H NMR Spectrum of **A1**

Figure S2: ^{13}C NMR Spectrum of **A1**

Figure S3: Mass Spectrum of **A1**

Figure S4: ^{1}H NMR Spectrum of **A2**

Figure S5: ^{13}C NMR Spectrum of **A2**

Figure S6: Mass Spectrum of **A2**

Figure S7: ^{1}H NMR Spectrum of **B1**

Figure S8: ^{13}C NMR Spectrum of **B1**

Figure S9: Mass Spectrum of **B1**

Figure S10: ^{1}H NMR Spectrum of **B2**

Figure S11: ^{13}C NMR Spectrum of **B2**

Figure S12: Mass Spectrum of **B2**

Figure S13: ^{1}H NMR Spectrum of **BTT-1**

Figure S14: ^{13}C NMR Spectrum of **BTT-1**

Figure S15: Mass Spectrum of **BTT-1**

Figure S16: ^{1}H NMR Spectrum of **BTT-2**

Figure S17: ^{13}C NMR Spectrum of **BTT-2**

Figure S18: Mass Spectrum of **BTT-2**

Figure S19: ^{1}H NMR Spectrum of **BTT-3**

Figure S20: ^{13}C NMR Spectrum of **BTT-3**

Figure S21: Mass Spectrum of **BTT-3**

Figure S22: ^{1}H NMR Spectrum of **BTT-4**

Figure S23: ^{13}C NMR Spectrum of **BTT-4**

Figure S24: Mass Spectrum of **BTT-4**

Figure S25: ^{1}H NMR Spectrum of **BTT-5**

Figure S26: ^{13}C NMR Spectrum of **BTT-5**

Figure S27: Mass Spectrum of **BTT-5**

Figure S28: ^{1}H NMR Spectrum of **BTT-6**

Figure S29: ^{13}C NMR Spectrum of **BTT-6**

Figure S30: Mass Spectrum of **BTT-6**

Figure S31: ^{1}H NMR Spectrum of **BTT-7**

Figure S32: ^{13}C NMR Spectrum of **BTT-7**

Figure S33: Mass Spectrum of **BTT-7**

Figure S34: ^{1}H NMR Spectrum of **BTT-8**

Figure S35: ^{13}C NMR Spectrum of **BTT-8**

Figure S36: Mass Spectrum of **BTT-8**

Figure S37: ^{1}H NMR Spectrum of **BTT-9**

Figure S38: ^{13}C NMR Spectrum of **BTT-9**

Figure S39: Mass Spectrum of **BTT-9**

Figure S40: ^{1}H NMR Spectrum of **BTT-10**

Figure S41: ^{13}C NMR Spectrum of **BTT-10**

Figure S42: Mass Spectrum of **BTT-10**

Figure S43: ^{1}H NMR Spectrum of **BTP-1**

Figure S44: ^{13}C NMR Spectrum of **BTP-1**

Figure S45: Mass Spectrum of **BTP-1**

Figure S46: ^{1}H NMR Spectrum of **BTP-2**

Figure S47: ^{13}C NMR Spectrum of **BTP-2**

Figure S48: Mass Spectrum of **BTP-2**

Figure S49: ^{1}H NMR Spectrum of **BTP-3**

Figure S50: ^{13}C NMR Spectrum of **BTP-3**

Figure S51: Mass Spectrum of **BTP-3**

Figure S52: ^{1}H NMR Spectrum of **BTP-4**

Figure S53: ^{13}C NMR Spectrum of **BTP-4**

Figure S54: Mass Spectrum of **BTP-4**

Figure S55: ^{1}H NMR Spectrum of **BTP-5**

Figure S56: ^{13}C NMR Spectrum of **BTP-5**

Figure S57: Mass Spectrum of **BTP-5**

Figure S58: ^{1}H NMR Spectrum of **BTP-6**

Figure S59: ^{13}C NMR Spectrum of **BTP-6**

Figure S60: Mass Spectrum of **BTP-6**

Figure S61: ^{1}H NMR Spectrum of **BTP-7**

Figure S62: ^{13}C NMR Spectrum of **BTP-7**

Figure S63: Mass Spectrum of **BTP-7**

Figure S64: ^{1}H NMR Spectrum of **BTP-8**

Figure S65: ^{13}C NMR Spectrum of **BTP-8**

Figure S66: Mass Spectrum of **BTP-8**

Figure S67: ^{1}H NMR Spectrum of **BTP-9**

Figure S68: ^{13}C NMR Spectrum of **BTP-9**

Figure S69: Mass Spectrum of **BTP-9**

Figure S70: ^{1}H NMR Spectrum of **BTP-10**

Figure S71: ^{13}C NMR Spectrum of **BTP-10**

Figure S72: Mass Spectrum of **BTP-10**

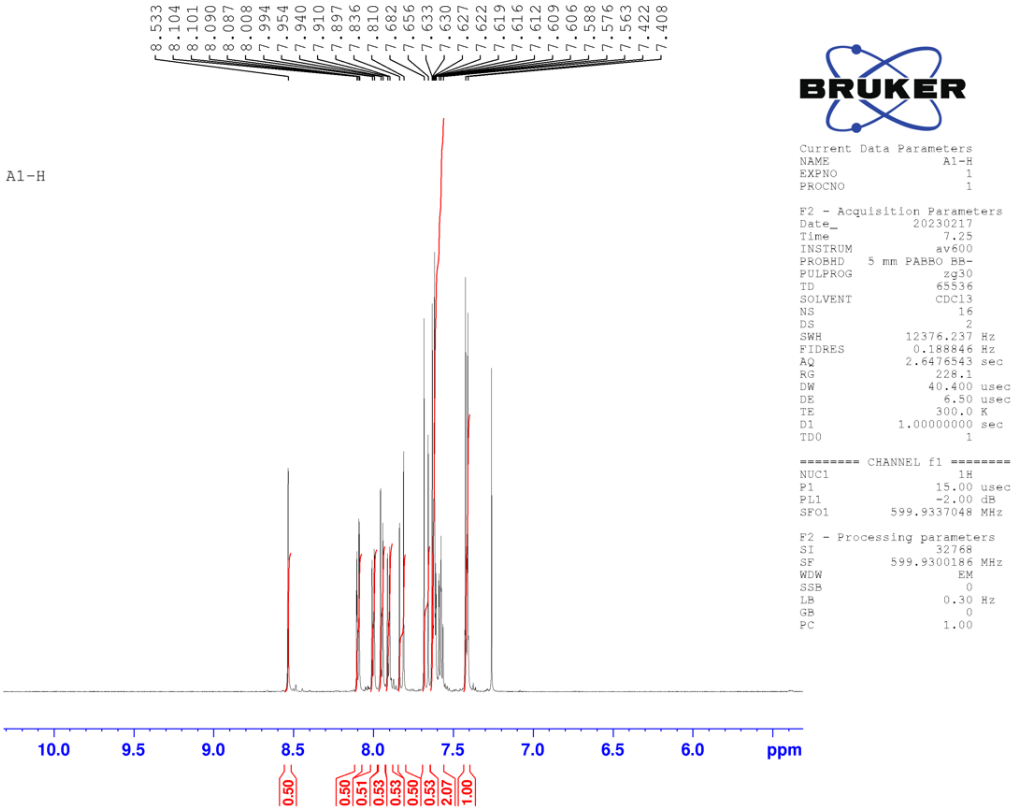

Figure S1. ^{1}H NMR Spectrum of **A1**

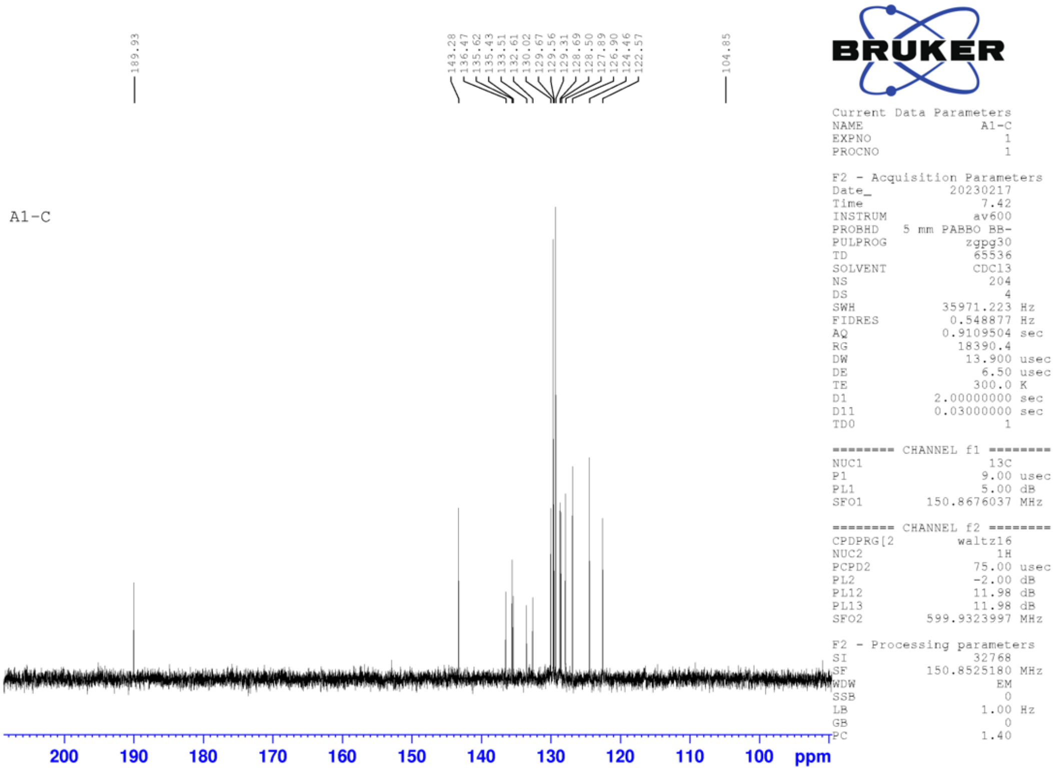

Figure S2. ^{13}C NMR Spectrum of **A1**

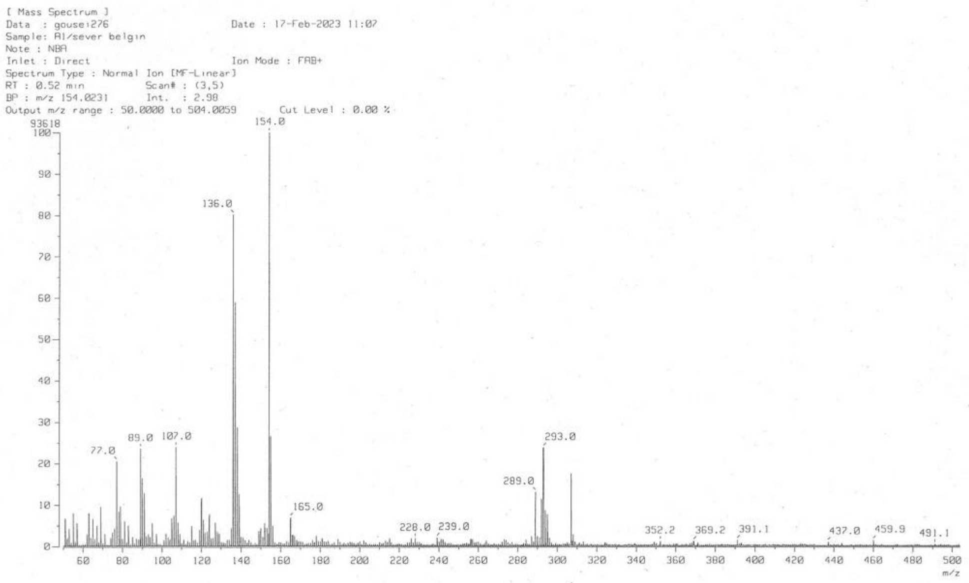

Figure S3: Mass Spectrum of **A1**

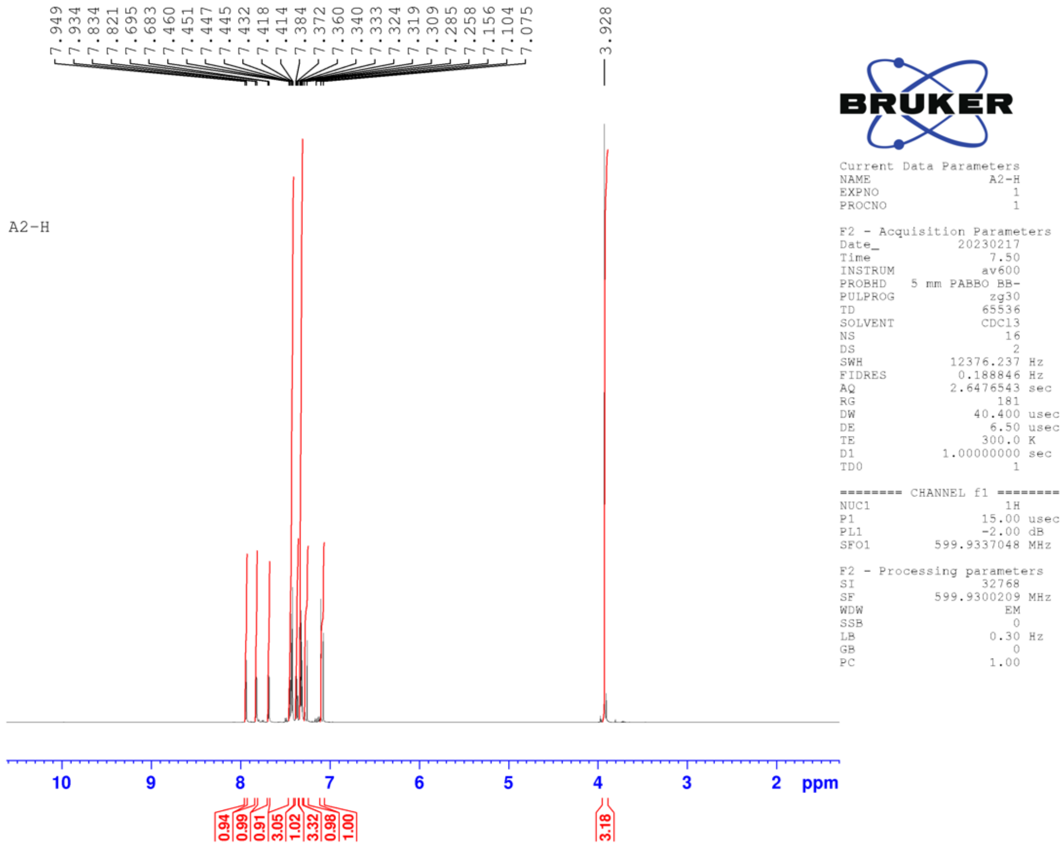

Figure S4: ^{1}H NMR Spectrum of **A2**

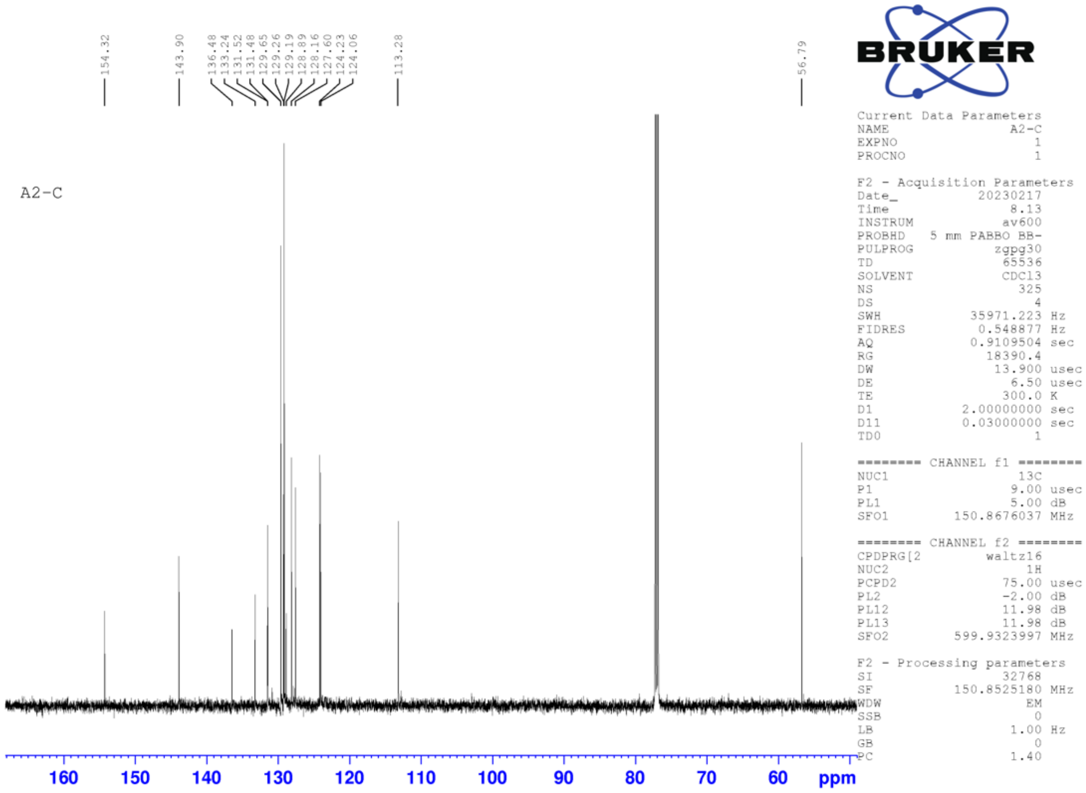

Figure S5: ^{13}C NMR Spectrum of **A2**

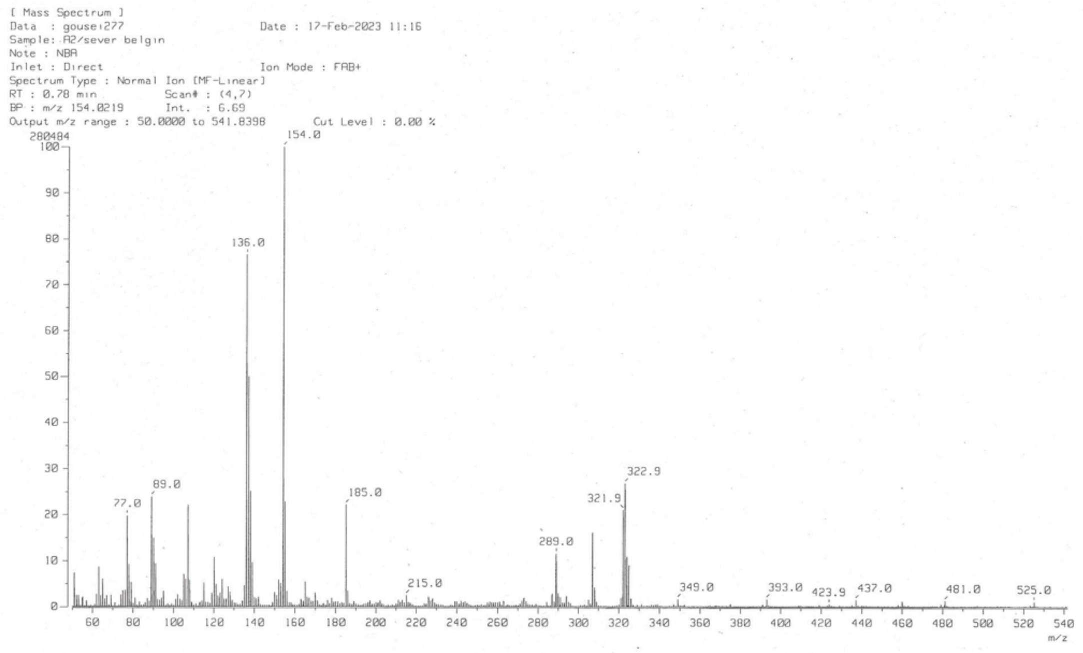

Figure S6: Mass Spectrum of **A2**

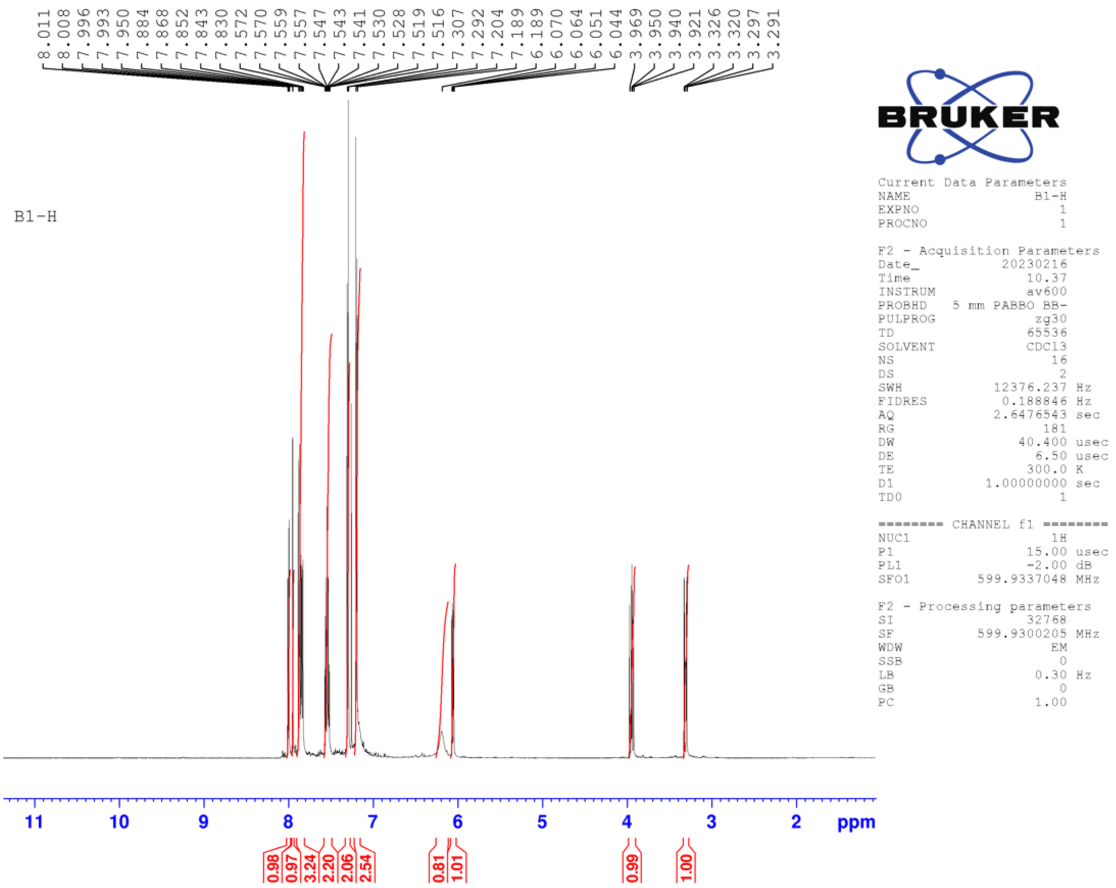

Figure S7: ^{1}H NMR Spectrum of **B1**

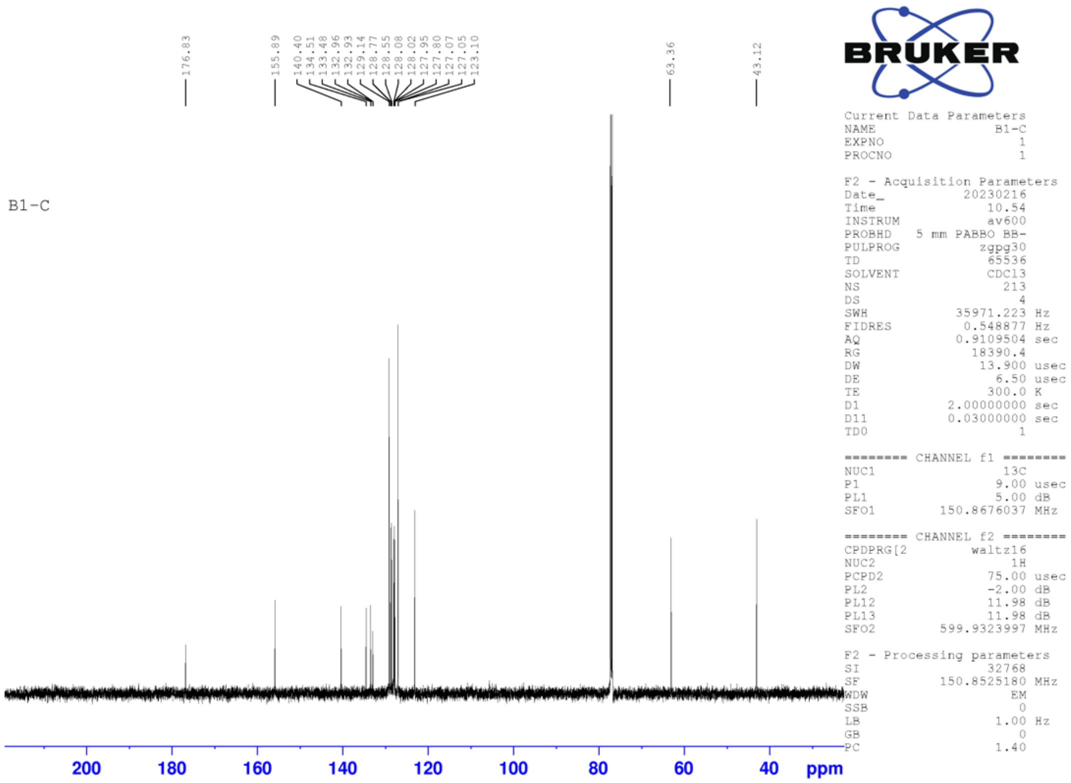

Figure S8: ^{13}C NMR Spectrum of **B1**

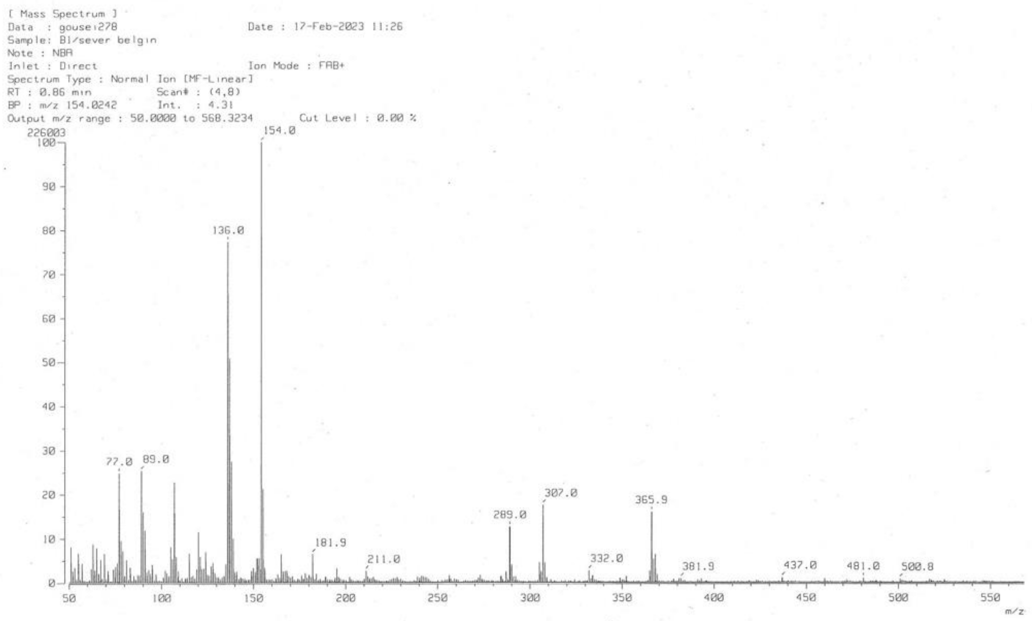

Figure S9: Mass Spectrum of **B1**

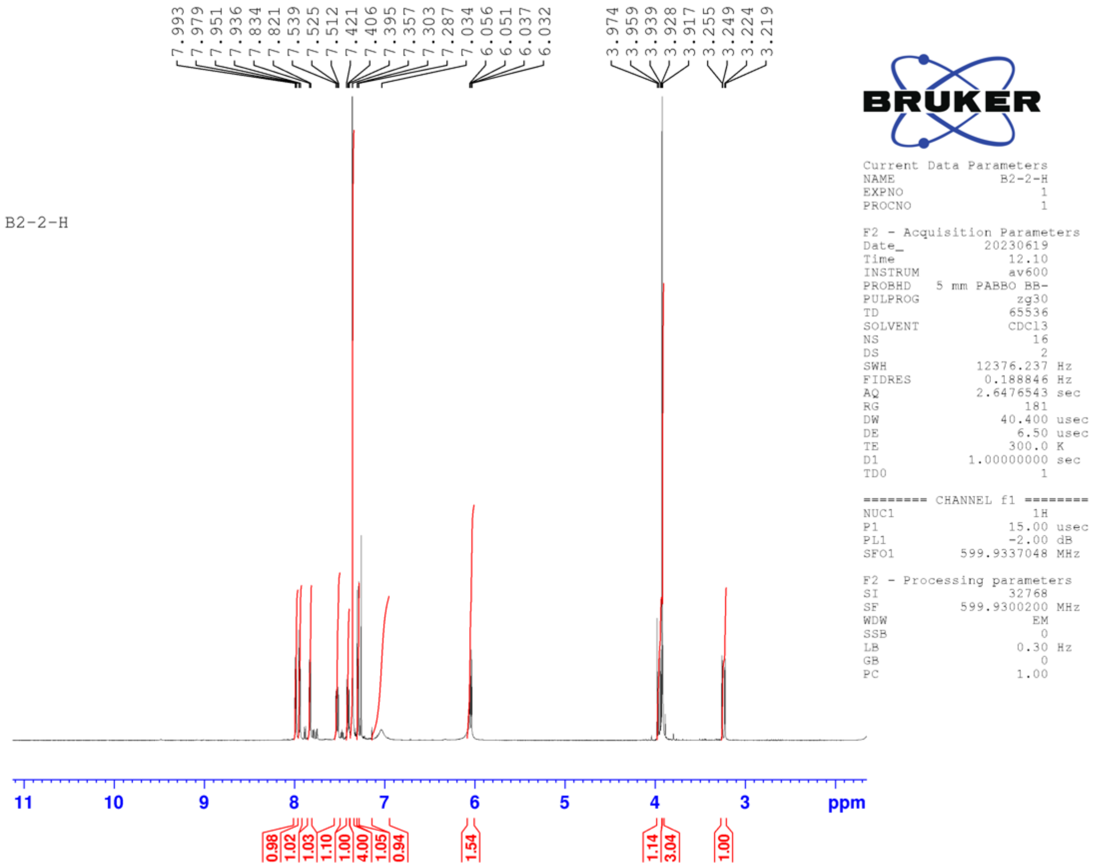

Figure S10: ^{1}H NMR Spectrum of **B2**

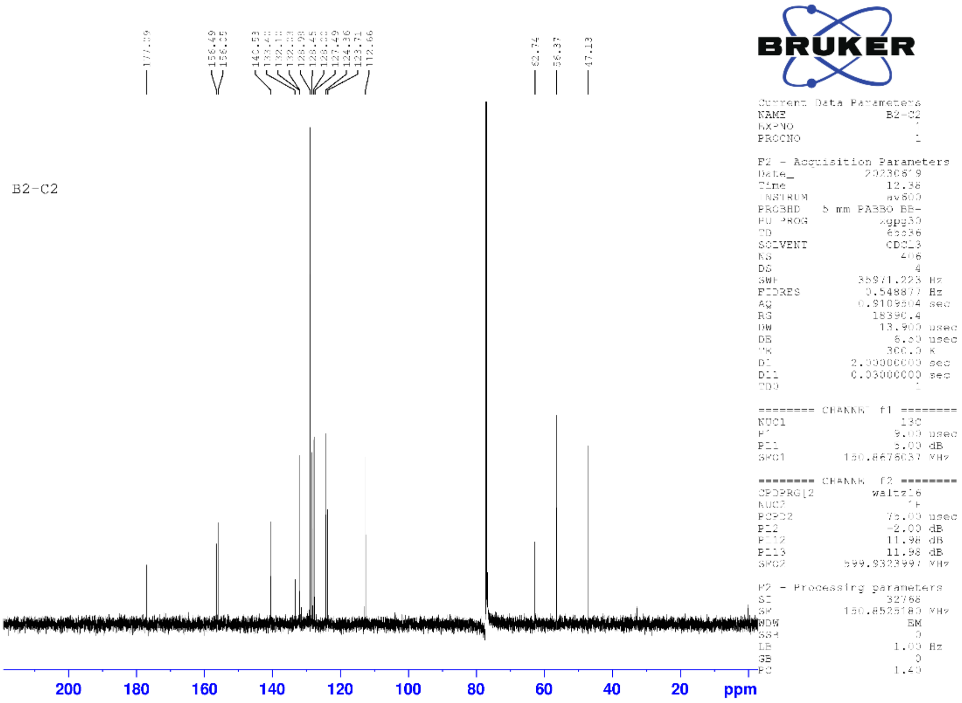

Figure S11: ^{13}C NMR Spectrum of **B2**

[Elemental Composition] Page: 1
Data : gousei279 Date : 17-Feb-2023 11:38
Sample: B2/sever belgin
Note : NBA
Inlet : Direct Ion Mode : FAB+
RT : 0.88 min Scan#: (2,7)
Elements : C 100/0, H 100/0, O 2/0, N 4/2, Cl 2/0, S 2/0
Mass Tolerance : 20ppm, 10mmu if m/z < 500, 20mmu if m/z > 1000
Unsaturation (U.S.) : -0.5 - 150.0

Observed m/z	Int%	Err[ppm / mmu]	U.S.	Composition
396.0926	100.0	+6.8 / +2.7	23.0	C 27 H 12 O 2 N 2
		-21.5 / -8.5	23.0	C 26 H 12 O N 4
		+5.6 / +2.2	18.5	C 24 H 15 O N 3 Cl
		+4.4 / +1.7	14.0	C 21 H 18 N 4 Cl 2
		-1.7 / -0.7	19.0	C 24 H 16 O 2 N 2 S
		-2.9 / -1.1	14.5	C 21 H 19 O N 3 Cl S
		+24.2 / +9.6	10.0	C 19 H 22 O N 2 Cl 2 S
		-4.1 / -1.6	10.0	C 18 H 22 N 4 Cl 2 S
		-10.2 / -4.0	15.0	C 21 H 20 O 2 N 2 S 2
		+21.6 / +8.5	15.5	C 20 H 18 O 2 N 3 S 2
		-11.4 / -4.5	10.5	C 18 H 23 O N 3 Cl S 2
		+20.3 / +8.1	11.0	C 17 H 21 O N 4 Cl S 2
		+15.7 / +6.2	6.0	C 16 H 26 O N 2 Cl 2 S 2
		-12.6 / -5.0	6.0	C 15 H 26 N 4 Cl 2 S 2

Figure S12: Mass Spectrum of **B2**

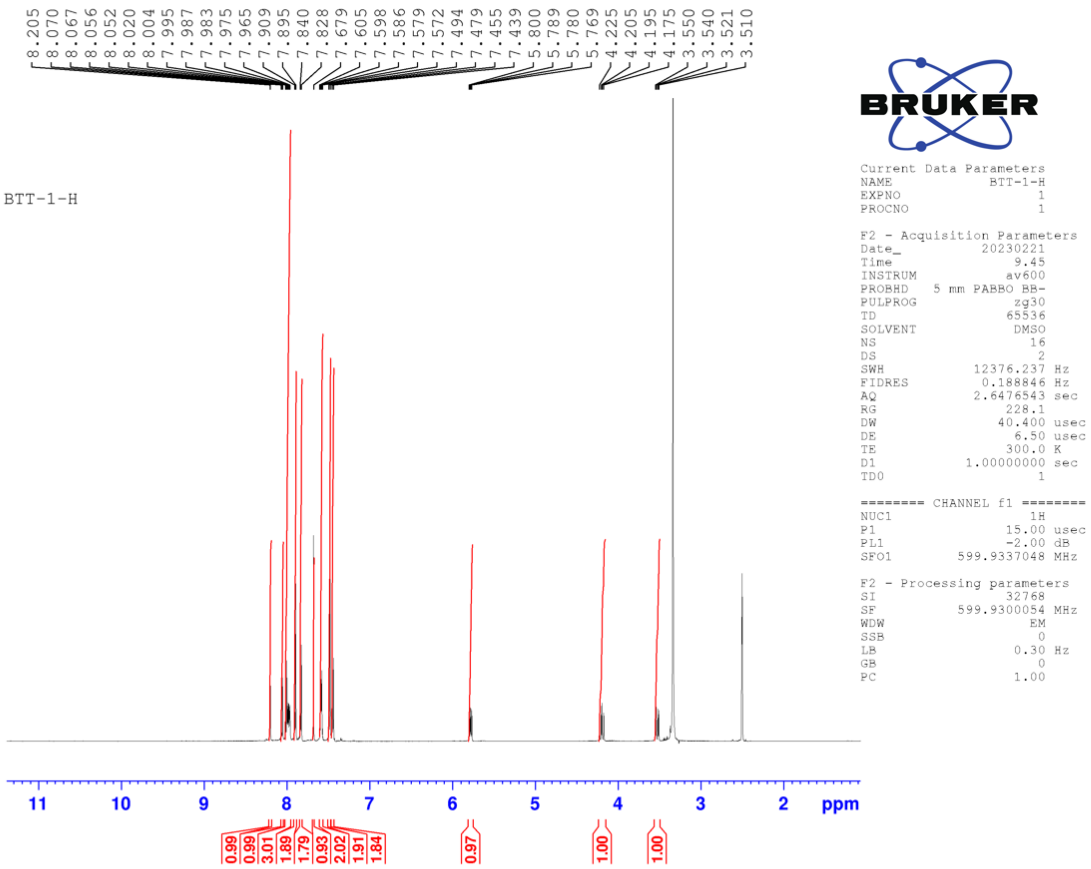

Figure S13: ^{1}H NMR Spectrum of **BTT-1**

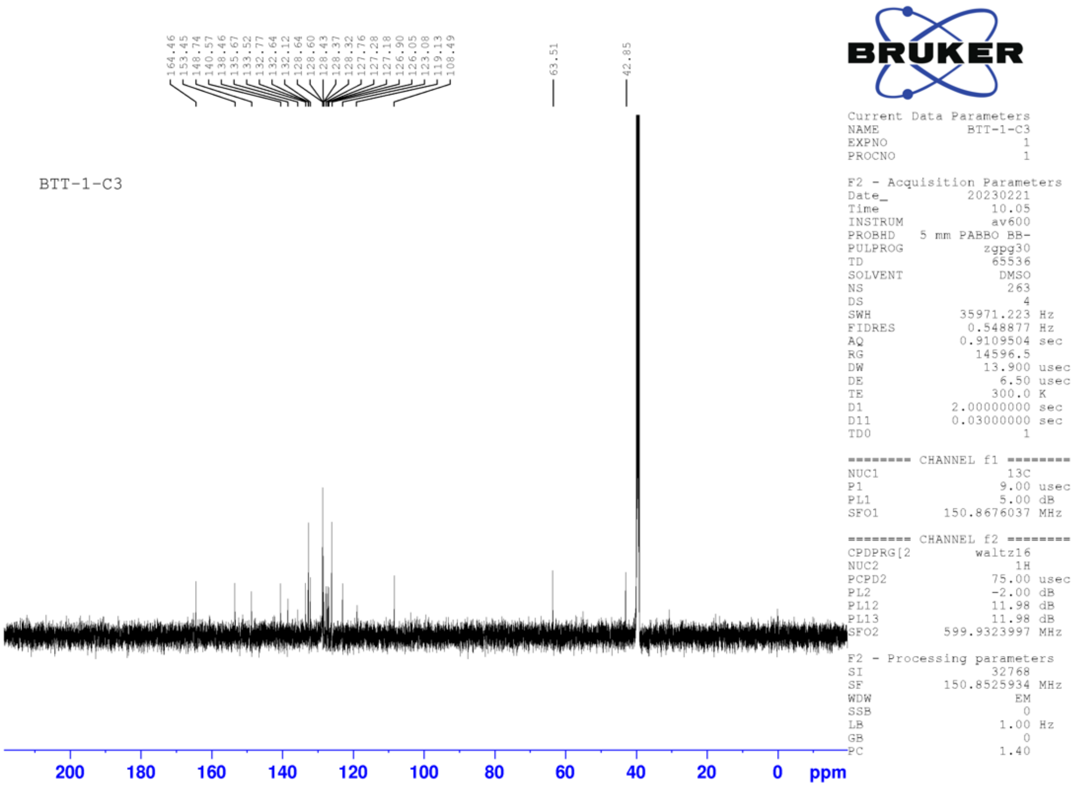

Figure S14: ^{13}C NMR Spectrum of **BTT-1**

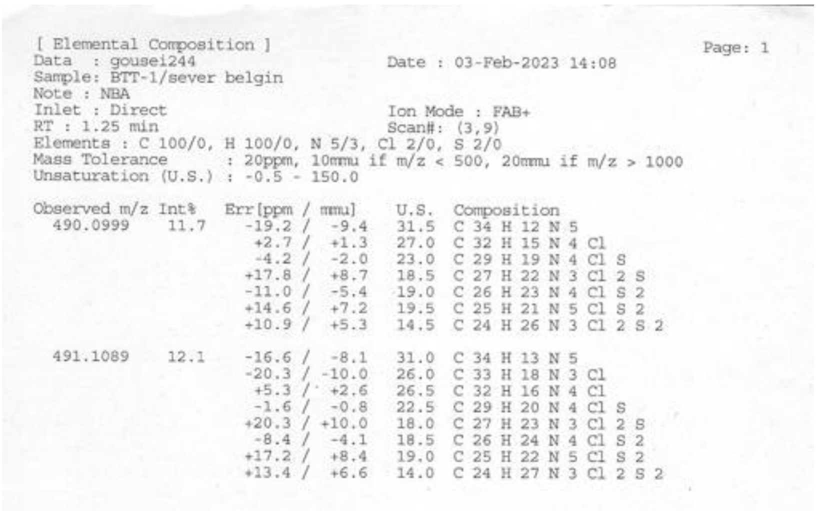

[Elemental Composition] Page: 1
Data : gousei244 Date : 03-Feb-2023 14:08
Sample: BTT-1/sever belgin
Note : NBA
Inlet : Direct Ion Mode : FAB+
RT : 1.25 min Scan#: (3,9)
Elements : C 100/0, H 100/0, N 5/3, Cl 2/0, S 2/0
Mass Tolerance : 20ppm, 10mmu if m/z < 500, 20mmu if m/z > 1000
Unsaturation (U.S.) : -0.5 - 150.0

Observed m/z	Int%	Err[ppm / mmu]	U.S.	Composition
490.0999	11.7	-19.2 / -9.4	31.5	C 34 H 12 N 5
		+2.7 / +1.3	27.0	C 32 H 15 N 4 Cl
		-4.2 / -2.0	23.0	C 29 H 19 N 4 Cl S
		+17.8 / +8.7	18.5	C 27 H 22 N 3 Cl 2 S
		-11.0 / -5.4	19.0	C 26 H 23 N 4 Cl S 2
		+14.6 / +7.2	19.5	C 25 H 21 N 5 Cl S 2
		+10.9 / +5.3	14.5	C 24 H 26 N 3 Cl 2 S 2
491.1089	12.1	-16.6 / -8.1	31.0	C 34 H 13 N 5
		-20.3 / -10.0	26.0	C 33 H 18 N 3 Cl
		+5.3 / +2.6	26.5	C 32 H 16 N 4 Cl
		-1.6 / -0.8	22.5	C 29 H 20 N 4 Cl S
		+20.3 / +10.0	18.0	C 27 H 23 N 3 Cl 2 S
		-8.4 / -4.1	18.5	C 26 H 24 N 4 Cl S 2
		+17.2 / +8.4	19.0	C 25 H 22 N 5 Cl S 2
		+13.4 / +6.6	14.0	C 24 H 27 N 3 Cl 2 S 2

Figure S15: Mass Spectrum of **BTT-1**

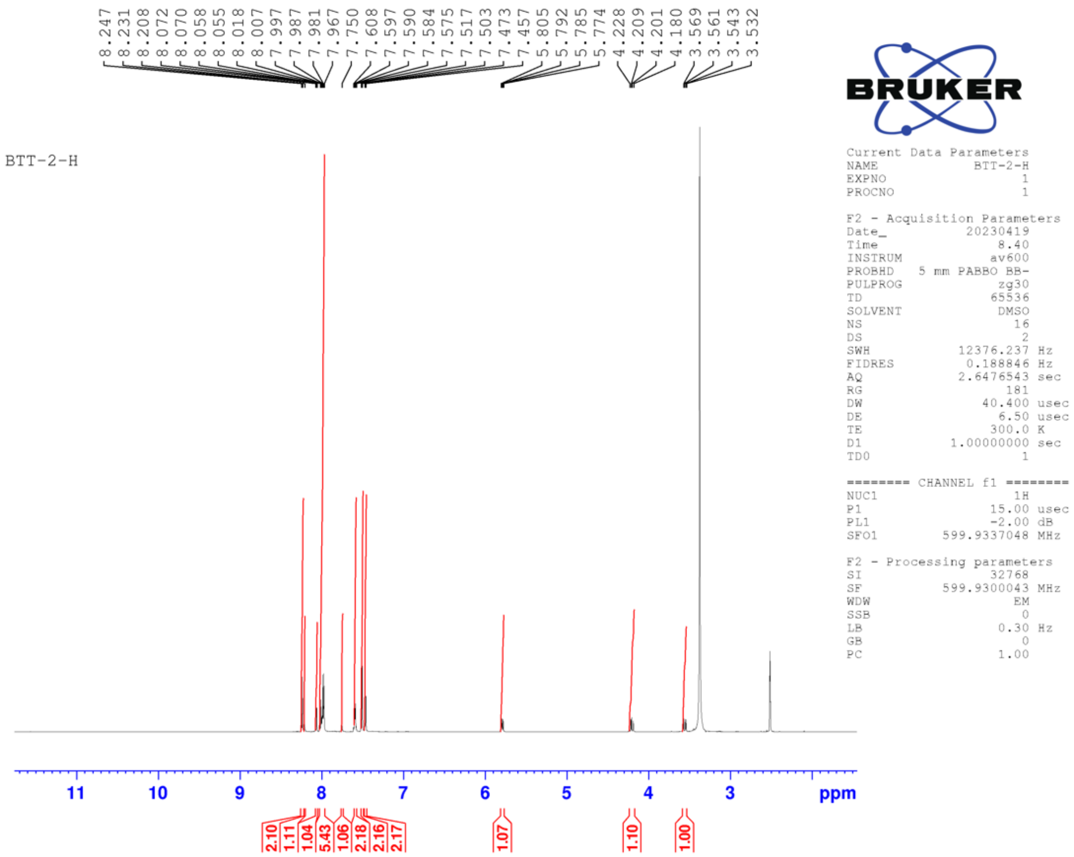

Figure S16: ^{1}H NMR Spectrum of **BTT-2**

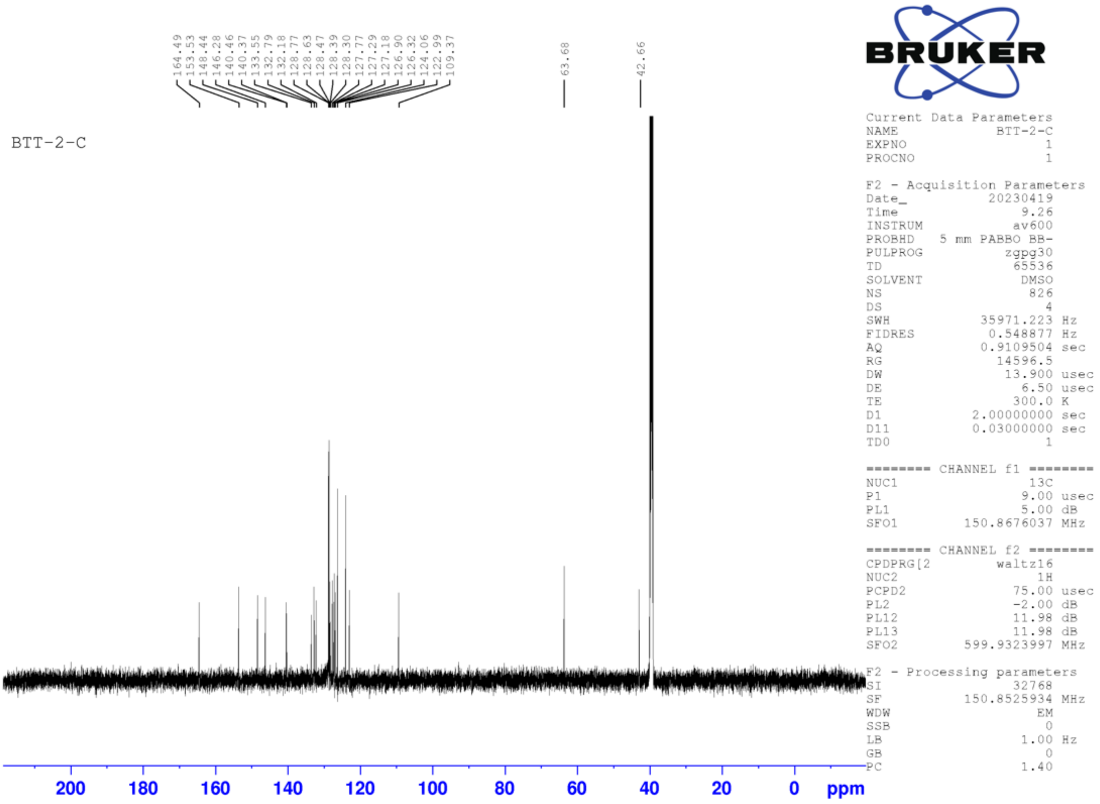

Figure S17: ^{13}C NMR Spectrum of **BTT-2**

[Elemental Composition] Page: 1
Data : gousei245 Date : 03-Feb-2023 14:25
Sample: BTT-2/sever belgin
Note : NBA
Inlet : Direct Ion Mode : FAB+
RT : 1.63 min Scan#: (5,10)
Elements : C 100/0, H 100/0, O 3/1, N 5/3, Cl 2/0, S 2/0
Mass Tolerance : 20ppm, 10mmu if m/z < 500, 20mmu if m/z > 1000
Unsaturation (U.S.) : -0.5 - 150.0

Observed m/z	Int%	Err[ppm / mmu]	U.S.	Composition
510.0926	50.5	+9.3 / +4.7	30.5	C 34 H 12 O 3 N 3
		-12.8 / -6.5	30.5	C 33 H 12 O 2 N 5
		-16.3 / -8.3	25.5	C 32 H 17 O 2 N 3 Cl
		+8.3 / +4.2	26.0	C 31 H 15 O 2 N 4 Cl
		-17.3 / -8.8	21.0	C 29 H 20 O N 4 Cl 2
		+7.4 / +3.7	21.5	C 28 H 18 O N 5 Cl 2
		+2.7 / +1.4	26.5	C 31 H 16 O 3 N 3 S
		-19.4 / -9.9	26.5	C 30 H 16 O 2 N 5 S
		+1.7 / +0.9	22.0	C 28 H 19 O 2 N 4 Cl S
		+0.7 / +0.4	17.5	C 25 H 22 O N 5 Cl 2 S
		-4.0 / -2.0	22.5	C 28 H 20 O 3 N 3 S 2
		-4.9 / -2.5	18.0	C 25 H 23 O 2 N 4 Cl S 2
		+19.7 / +10.1	18.5	C 24 H 21 O 2 N 5 Cl S 2
		+16.2 / +8.2	13.5	C 23 H 26 O 2 N 3 Cl 2 S 2
		-5.9 / -3.0	13.5	C 22 H 26 O N 5 Cl 2 S 2
511.0974	36.4	+3.4 / +1.7	30.0	C 34 H 13 O 3 N 3
		-18.6 / -9.5	30.0	C 33 H 13 O 2 N 5
		+2.4 / +1.2	25.5	C 31 H 16 O 2 N 4 Cl
		+1.5 / +0.7	21.0	C 28 H 19 O N 5 Cl 2
		-3.2 / -1.7	26.0	C 31 H 17 O 3 N 3 S
		-4.2 / -2.1	21.5	C 28 H 20 O 2 N 4 Cl S
		+16.8 / +8.6	17.0	C 26 H 23 O 2 N 3 Cl 2 S
		-5.1 / -2.6	17.0	C 25 H 23 O N 5 Cl 2 S
		-9.8 / -5.0	22.0	C 28 H 21 O 3 N 3 S 2
		+14.8 / +7.6	22.5	C 27 H 19 O 3 N 4 S 2
		-10.8 / -5.5	17.5	C 25 H 24 O 2 N 4 Cl S 2
		+13.8 / +7.1	18.0	C 24 H 22 O 2 N 5 Cl S 2
		+10.2 / +5.2	13.0	C 23 H 27 O 2 N 3 Cl 2 S 2
		-11.7 / -6.0	13.0	C 22 H 27 O N 5 Cl 2 S 2

Figure S18: Mass Spectrum of **BTT-2**

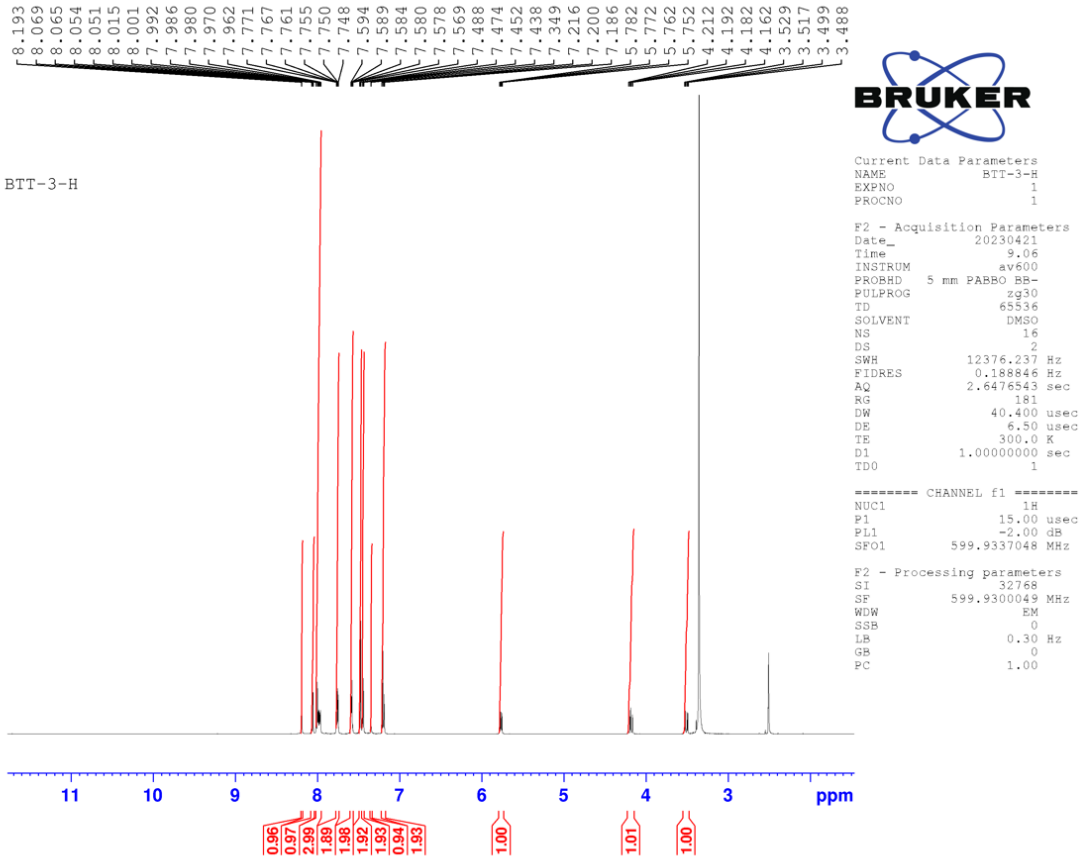

Figure S19: ^{1}H NMR Spectrum of **BTT-3**

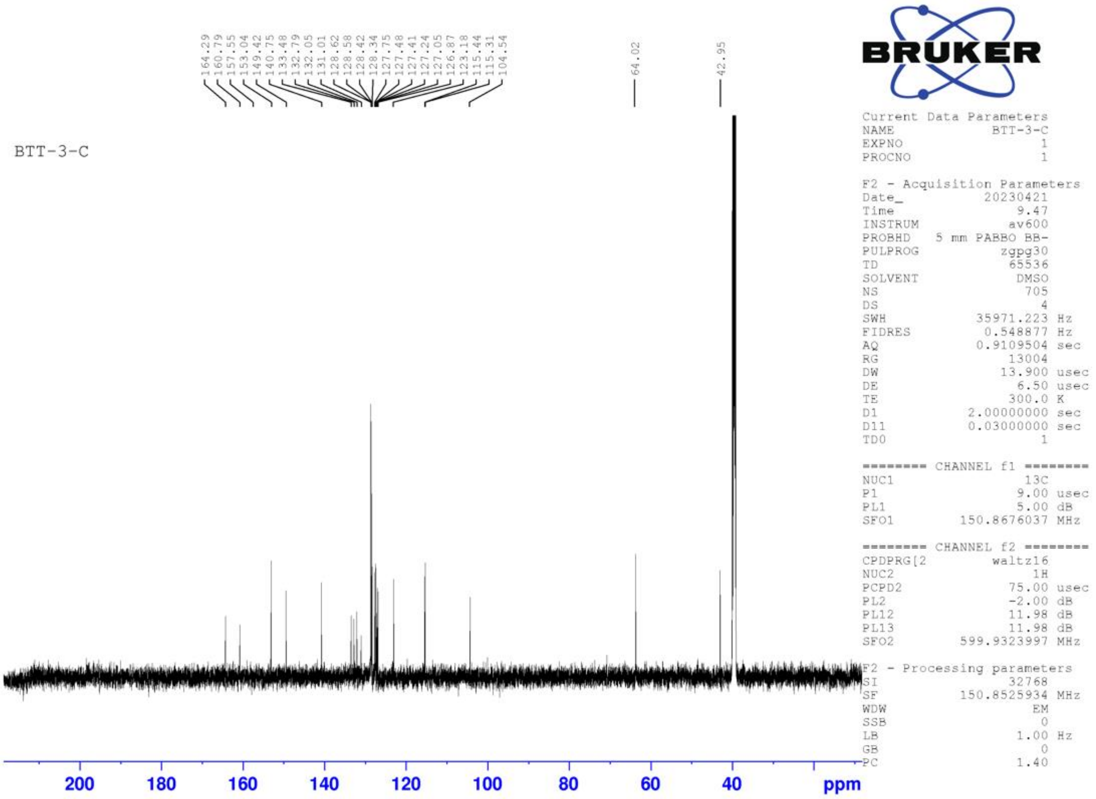

Figure S20: ^{13}C NMR Spectrum of **BTT-3**

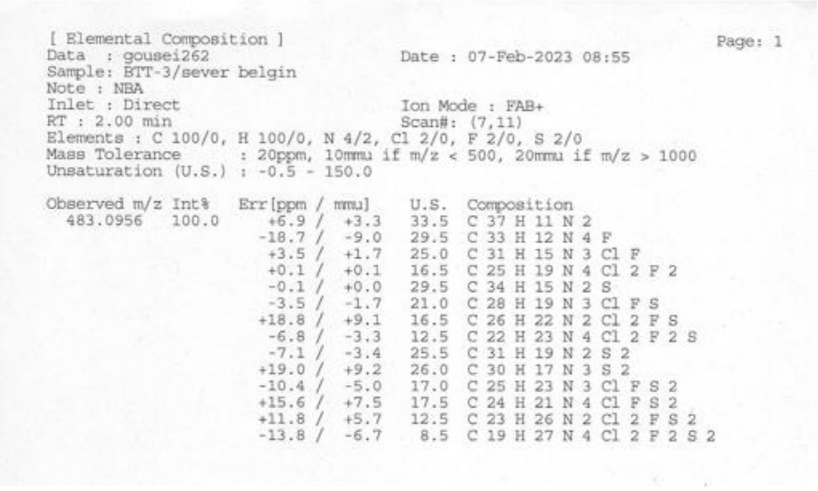

[Elemental Composition] Page: 1
Data : gousei262 Date : 07-Feb-2023 08:55
Sample: BTT-3/sever belgin
Note : NBA
Inlet : Direct Ion Mode : FAB+
RT : 2.00 min Scan#: (7,11)
Elements : C 100/0, H 100/0, N 4/2, Cl 2/0, F 2/0, S 2/0
Mass Tolerance : 20ppm, 10mmu if m/z < 500, 20mmu if m/z > 1000
Unsaturation (U.S.) : -0.5 - 150.0

Observed m/z	Int%	Err[ppm / mmu]	U.S.	Composition
483.0956	100.0	+6.9 / +3.3	33.5	C 37 H 11 N 2
		-18.7 / -9.0	29.5	C 33 H 12 N 4 F
		+3.5 / +1.7	25.0	C 31 H 15 N 3 Cl F
		+0.1 / +0.1	16.5	C 25 H 19 N 4 Cl 2 F 2
		-0.1 / +0.0	29.5	C 34 H 15 N 2 S
		-3.5 / -1.7	21.0	C 28 H 19 N 3 Cl F S
		+18.8 / +9.1	16.5	C 26 H 22 N 2 Cl 2 F S
		-6.8 / -3.3	12.5	C 22 H 23 N 4 Cl 2 F 2 S
		-7.1 / -3.4	25.5	C 31 H 19 N 2 S 2
		+19.0 / +9.2	26.0	C 30 H 17 N 3 S 2
		-10.4 / -5.0	17.0	C 25 H 23 N 3 Cl F S 2
		+15.6 / +7.5	17.5	C 24 H 21 N 4 Cl F S 2
		+11.8 / +5.7	12.5	C 23 H 26 N 2 Cl 2 F S 2
		-13.8 / -6.7	8.5	C 19 H 27 N 4 Cl 2 F 2 S 2

Figure S21: Mass Spectrum of **BTT-3**

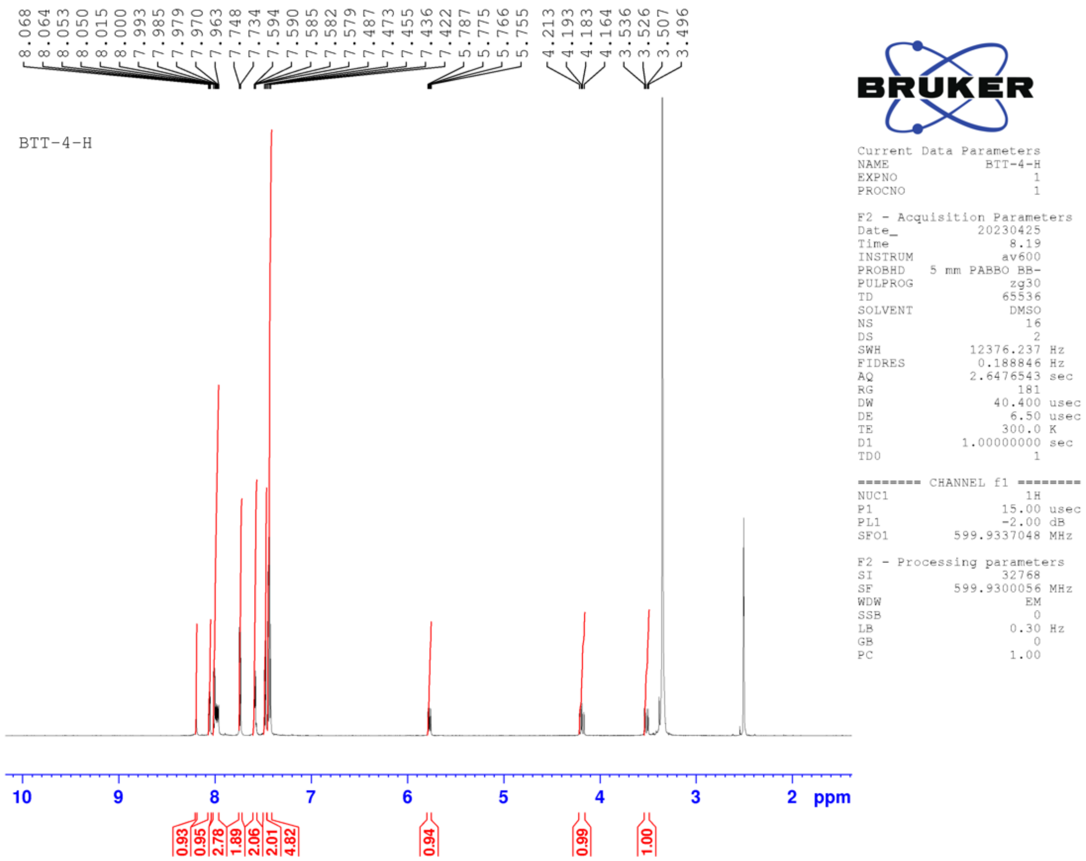

Figure S22: ^{1}H NMR Spectrum of **BTT-4**

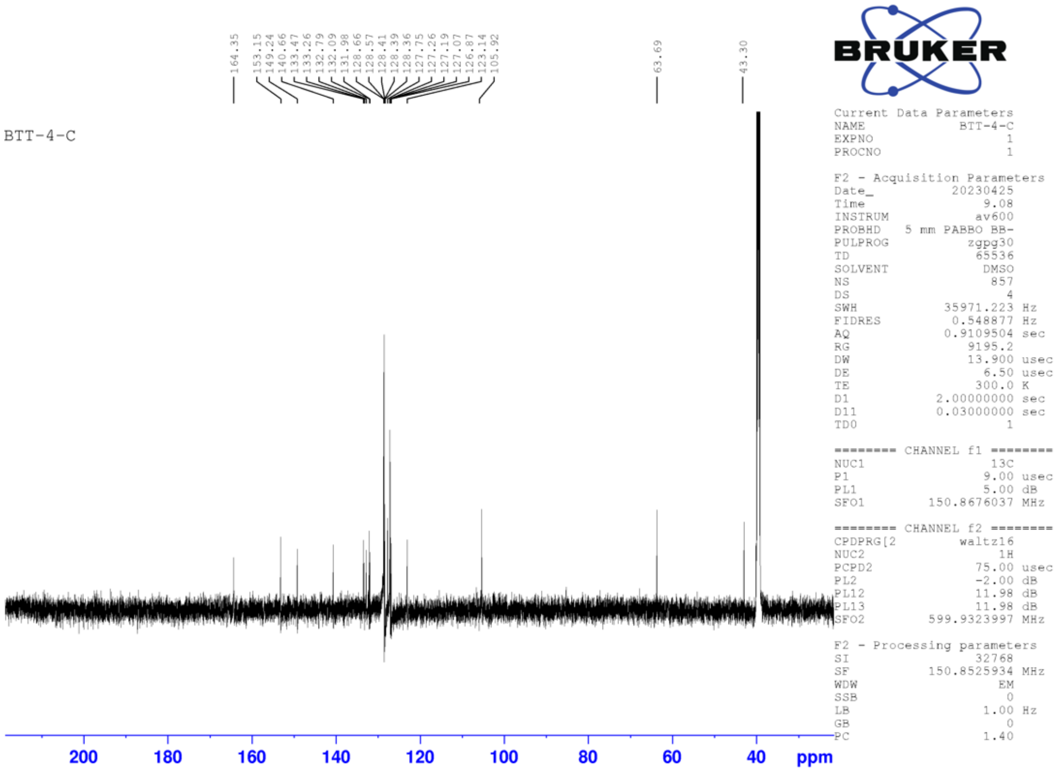

Figure S23: ^{13}C NMR Spectrum of **BTT-4**

[Elemental Composition] Page: 1
Data : gousei263 Date : 07-Feb-2023 09:10
Sample: BTT-4/sever belgin
Note : NBA
Inlet : Direct Ion Mode : FAB+
RT : 1.13 min Scan#: (3,8)
Elements : C 100/0, H 100/0, N 4/2, Cl 3/1, S 2/0
Mass Tolerance : 20ppm, 10mmu if m/z < 500, 20mmu if m/z > 1000
Unsaturation (U.S.) : -0.5 - 150.0

Observed m/z	Int%	Err[ppm / mmu]	U.S.	Composition
499.0660	23.2	-18.2 / -9.1	29.5	C 33 H 12 N 4 Cl
		+3.4 / +1.7	25.0	C 31 H 15 N 3 Cl 2
		-3.4 / -1.7	21.0	C 28 H 19 N 3 Cl 2 S
		+18.1 / +9.1	16.5	C 26 H 22 N 2 Cl 3 S
		-10.2 / -5.1	17.0	C 25 H 23 N 3 Cl 2 S 2
		+15.0 / +7.5	17.5	C 24 H 21 N 4 Cl 2 S 2
		+11.4 / +5.7	12.5	C 23 H 26 N 2 Cl 3 S 2

Figure S24: Mass Spectrum of **BTT-4**

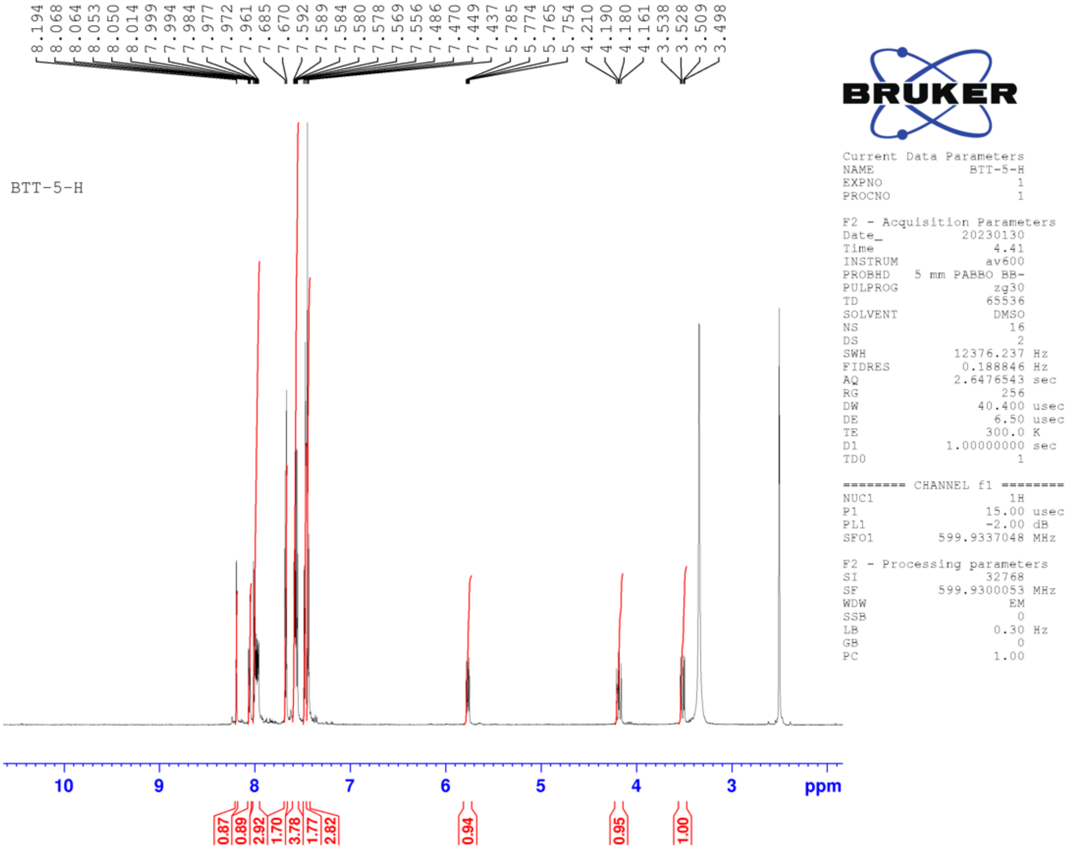

Figure S25: ^{1}H NMR Spectrum of **BTT-5**

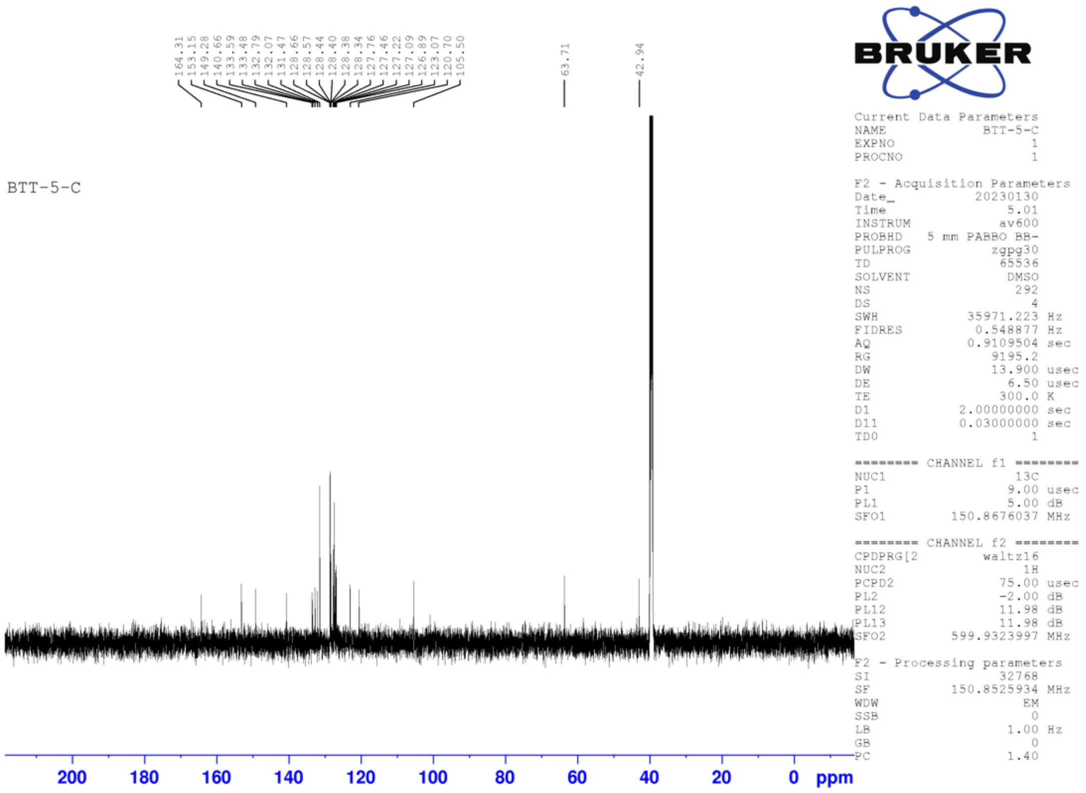

Figure S26: ^{13}C NMR Spectrum of **BTT-5**

[Elemental Composition] Page: 1
Data : gousei240 Date : 30-Jan-2023 16:09
Sample: BTT-5/sever belgin
Note : NBA
Inlet : Direct Ion Mode : FAB+
RT : 1.50 min Scan#: (4,10)
Elements : C 100/0, H 100/0, N 4/2, Cl 2/0, Br 2/0, S 2/0
Mass Tolerance : 20ppm, 10mmu if m/z < 500, 20mmu if m/z > 1000
Unsaturation (U.S.) : -0.5 - 150.0

Observed m/z	Int%	Err[ppm / mmu]	U.S.	Composition
544.0172	8.6	-8.2 / -4.5	24.5	C 31 H 16 N 3 Cl Br
		+14.9 / +8.1	25.0	C 30 H 14 N 4 Cl Br
		+11.6 / +6.3	20.0	C 29 H 19 N 2 Cl 2 Br
		+4.0 / +2.2	18.0	C 28 H 22 N 2 Br 2
		+14.0 / +7.6	41.0	C 40 H 4 N 2 S
		-14.4 / -7.8	20.5	C 28 H 20 N 3 Cl Br S
		+8.7 / +4.7	21.0	C 27 H 18 N 4 Cl Br S
		+5.4 / +2.9	16.0	C 26 H 23 N 2 Cl 2 Br S
		-2.2 / -1.2	14.0	C 25 H 26 N 2 Br 2 S
		+7.8 / +4.3	37.0	C 37 H 8 N 2 S 2
		+2.5 / +1.4	17.0	C 24 H 22 N 4 Cl Br S 2
		-0.8 / -0.5	12.0	C 23 H 27 N 2 Cl 2 Br S 2
		-8.4 / -4.6	10.0	C 22 H 30 N 2 Br 2 S 2
		+14.7 / +8.0	10.5	C 21 H 28 N 3 Br 2 S 2
543.0135	8.3	-12.7 / -6.9	31.5	C 34 H 9 N 4 Cl 2
		-0.4 / -0.2	25.0	C 31 H 15 N 3 Cl Br
		+19.4 / +10.5	20.5	C 29 H 18 N 2 Cl 2 Br
		+11.8 / +6.4	18.5	C 28 H 21 N 2 Br 2
		-18.9 / -10.2	27.5	C 31 H 13 N 4 Cl 2 S
		-6.6 / -3.6	21.0	C 28 H 19 N 3 Cl Br S
		+16.5 / +9.0	21.5	C 27 H 17 N 4 Cl Br S
		+13.1 / +7.1	16.5	C 26 H 22 N 2 Cl 2 Br S
		+5.6 / +3.0	14.5	C 25 H 25 N 2 Br 2 S
		+15.6 / +8.5	37.5	C 37 H 7 N 2 S 2
		-12.9 / -7.0	17.0	C 25 H 23 N 3 Cl Br S 2
		+10.3 / +5.6	17.5	C 24 H 21 N 4 Cl Br S 2
		+6.9 / +3.8	12.5	C 23 H 26 N 2 Cl 2 Br S 2
		-0.6 / -0.3	10.5	C 22 H 29 N 2 Br 2 S 2

Figure S27: Mass Spectrum of **BTT-5**

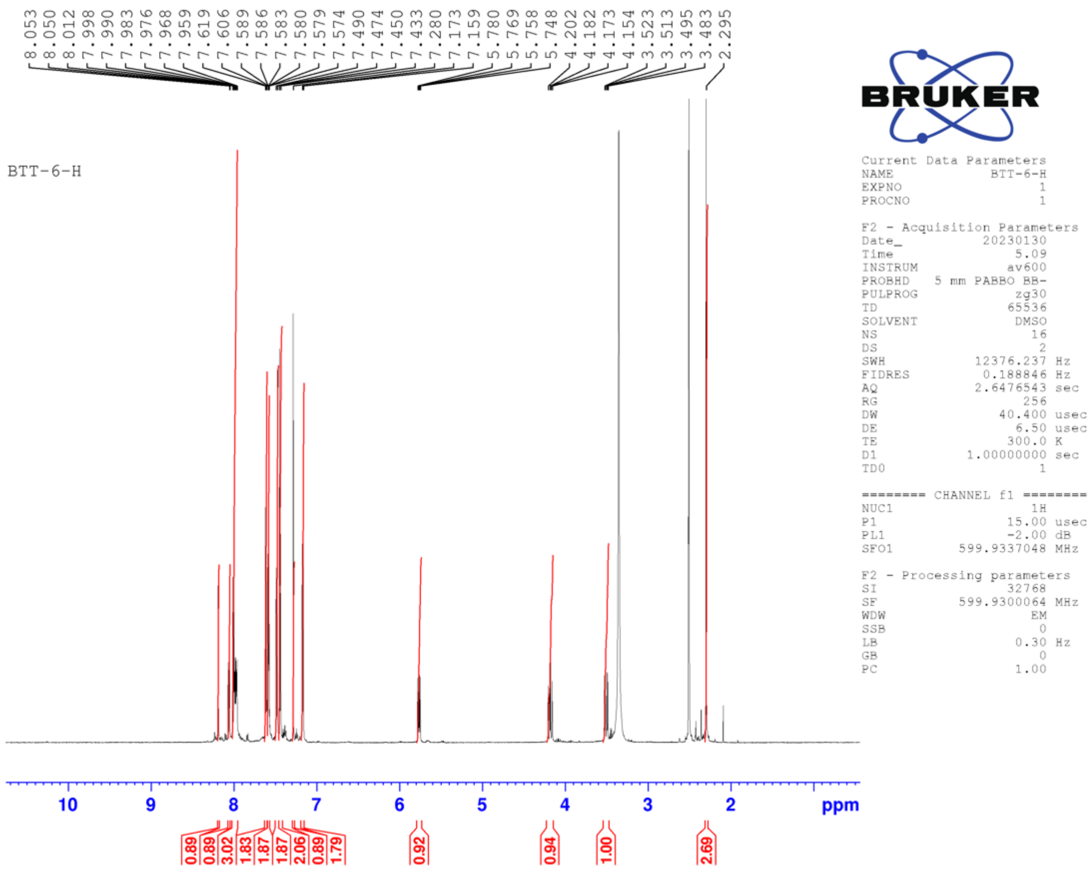

Figure S28: ^{1}H NMR Spectrum of **BTT-6**

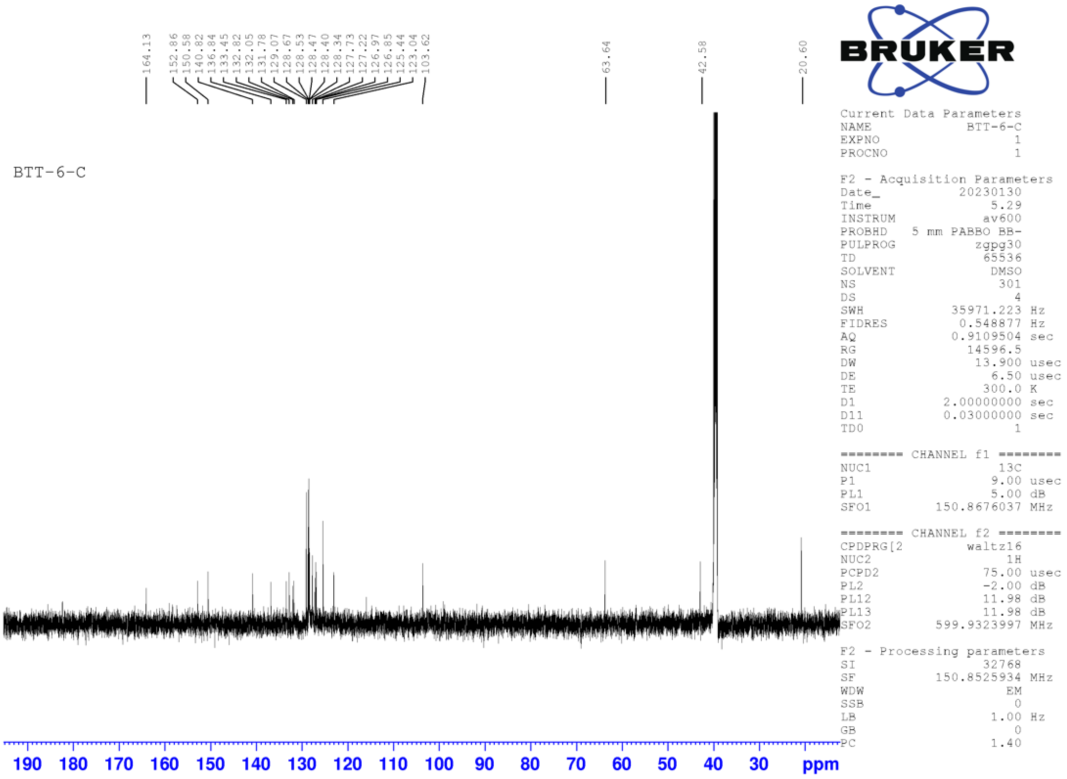

Figure S29: ^{13}C NMR Spectrum of **BTT-6**

[Elemental Composition]   Page: 1
Data : gousei241   Date : 30-Jan-2023 16:32
Sample: BTT-6/sever belgin
Note : NBA
Inlet : Direct   Ion Mode : FAB+
RT : 1.88 min   Scan#: (5,12)
Elements : C 100/0, H 100/0, N 4/2, Cl 2/0, S 2/0
Mass Tolerance : 20ppm, 10mmu if m/z < 500, 20mmu if m/z > 1000
Unsaturation (U.S.) : -0.5 - 150.0

Observed m/z	Int%	Err[ppm / mmu]	U.S.	Composition
480.1268	12.9	+0.1 / +0.1	24.5	C 32 H 19 N 3 Cl
		-6.9 / -3.3	20.5	C 29 H 23 N 3 Cl S
		+19.3 / +9.3	21.0	C 28 H 21 N 4 Cl S
		+15.5 / +7.4	16.0	C 27 H 26 N 2 Cl 2 S
		-13.9 / -6.7	16.5	C 26 H 27 N 3 Cl S 2
		+12.3 / +5.9	17.0	C 25 H 25 N 4 Cl S 2
		+8.4 / +4.1	12.0	C 24 H 30 N 2 Cl 2 S 2
479.1207	13.9	-18.8 / -9.0	29.5	C 34 H 15 N 4
		+3.6 / +1.7	25.0	C 32 H 18 N 3 Cl
		-3.4 / -1.6	21.0	C 29 H 22 N 3 Cl S
		+19.0 / +9.1	16.5	C 27 H 25 N 2 Cl 2 S
		-10.4 / -5.0	17.0	C 26 H 26 N 3 Cl S 2
		+15.8 / +7.6	17.5	C 25 H 24 N 4 Cl S 2
		+12.0 / +5.7	12.5	C 24 H 29 N 2 Cl 2 S 2

Figure S30: Mass Spectrum of **BTT-6**

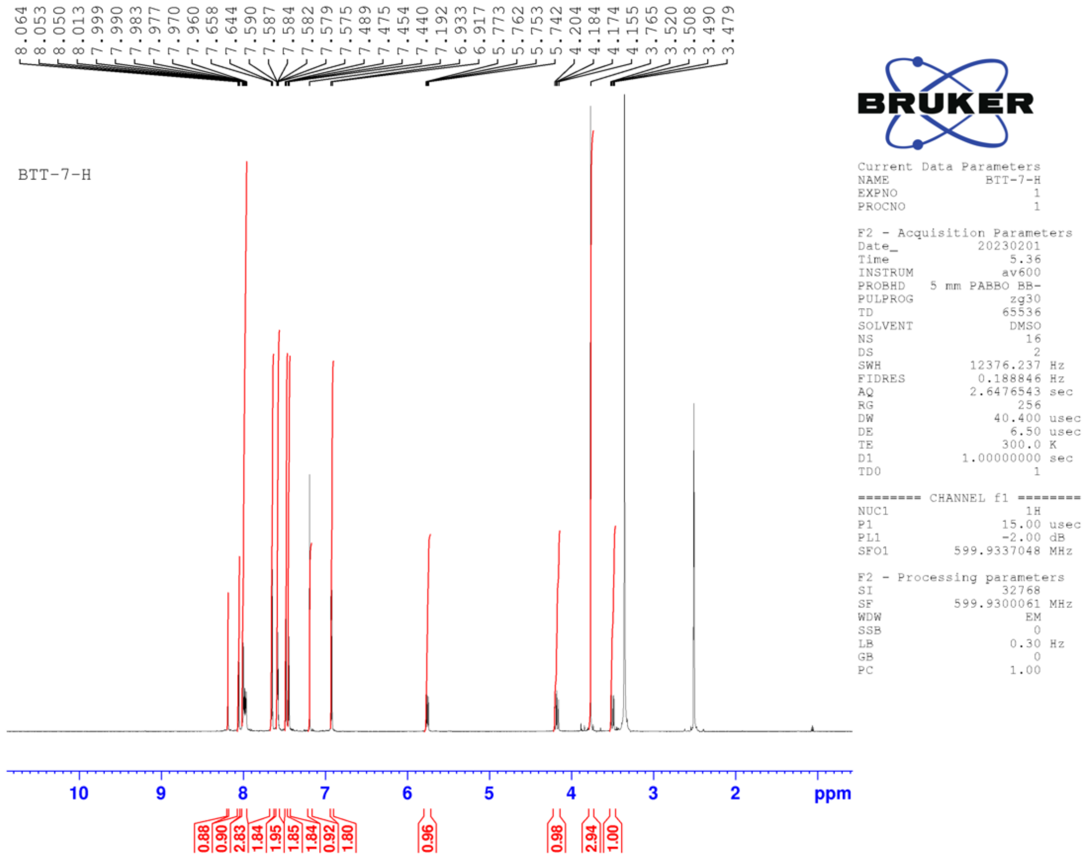

Figure S31: ^{1}H NMR Spectrum of **BTT-7**

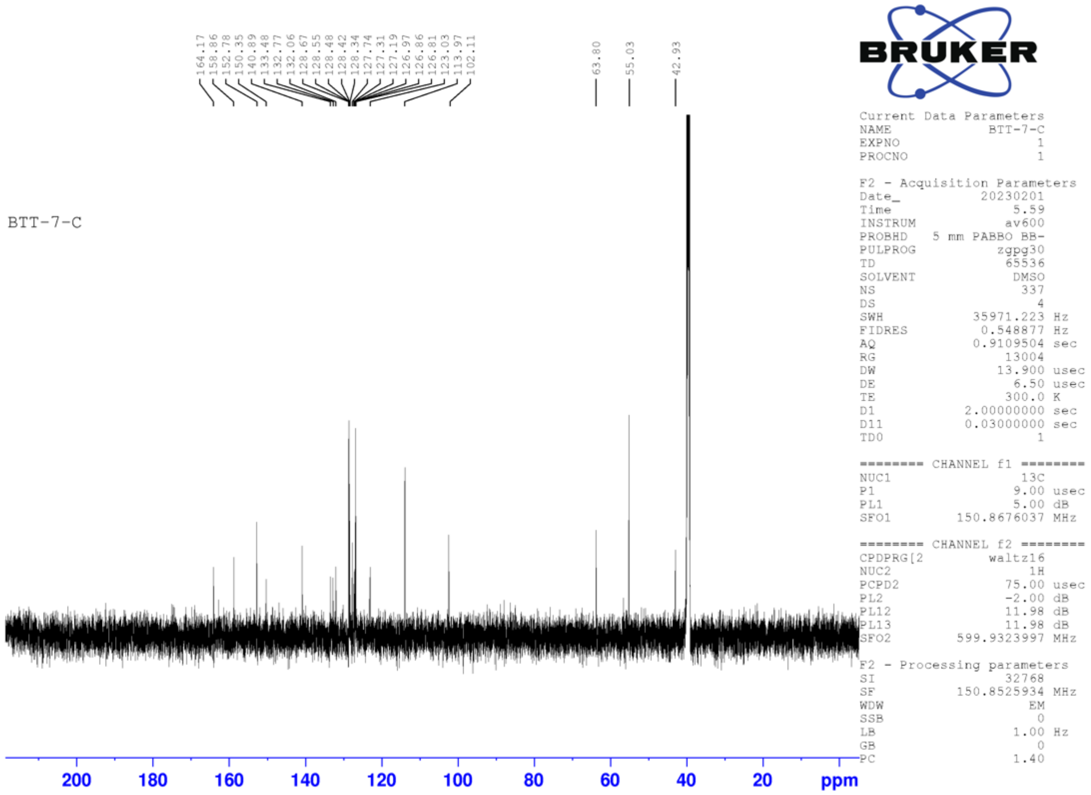

Figure S32: ^{13}C NMR Spectrum of **BTT-7**

[Elemental Composition] Page: 1
Data : gousei242 Date : 01-Feb-2023 15:35
Sample: BTT-7/sever belgin
Note : NBA
Inlet : Direct Ion Mode : FAB+
RT : 1.75 min Scan#: (4,12)
Elements : C 100/0, H 100/0, O 2/0, N 4/2, Cl 2/0, S 2/0
Mass Tolerance : 20ppm, 10mmu if m/z < 500, 20mmu if m/z > 1000
Unsaturation (U.S.) : -0.5 - 150.0

Observed m/z	Int%	Err[ppm / mmu]	U.S.	Composition
495.1172	35.8	+7.7 / +3.8	29.5	C 35 H 15 O 2 N 2
		-14.9 / -7.4	29.5	C 34 H 15 O N 4
		-18.6 / -9.2	24.5	C 33 H 20 O N 2 Cl
		+6.8 / +3.3	25.0	C 32 H 18 O N 3 Cl
		-19.6 / -9.7	20.0	C 30 H 23 N 3 Cl 2
		+5.8 / +2.9	20.5	C 29 H 21 N 4 Cl 2
		+0.9 / +0.5	25.5	C 32 H 19 O 2 N 2 S
		+0.0 / +0.0	21.0	C 29 H 22 O N 3 Cl S
		-1.0 / -0.5	16.5	C 26 H 25 N 4 Cl 2 S
		-5.9 / -2.9	21.5	C 29 H 23 O 2 N 2 S 2
		+19.5 / +9.7	22.0	C 28 H 21 O 2 N 3 S 2
		-6.9 / -3.4	17.0	C 26 H 26 O N 3 Cl S 2
		+18.5 / +9.2	17.5	C 25 H 24 O N 4 Cl S 2
		+14.8 / +7.3	12.5	C 24 H 29 O N 2 Cl 2 S 2
		-7.8 / -3.9	12.5	C 23 H 29 N 4 Cl 2 S 2
496.1177	34.3	-7.0 / -3.5	29.0	C 35 H 16 O 2 N 2
		+18.4 / +9.1	29.5	C 34 H 14 O 2 N 3
		-8.0 / -4.0	24.5	C 32 H 19 O N 3 Cl
		+17.4 / +8.6	25.0	C 31 H 17 O N 4 Cl
		+13.7 / +6.8	20.0	C 30 H 22 O N 2 Cl 2
		-9.0 / -4.4	20.0	C 29 H 22 N 4 Cl 2
		-13.8 / -6.8	25.0	C 32 H 20 O 2 N 2 S
		+11.6 / +5.7	25.5	C 31 H 18 O 2 N 3 S
		-14.8 / -7.3	20.5	C 29 H 23 O N 3 Cl S
		+10.6 / +5.2	21.0	C 28 H 21 O N 4 Cl S
		+6.9 / +3.4	16.0	C 27 H 26 O N 2 Cl 2 S
		-15.8 / -7.8	16.0	C 26 H 26 N 4 Cl 2 S
		+4.8 / +2.4	21.5	C 28 H 22 O 2 N 3 S 2
		+3.8 / +1.9	17.0	C 25 H 25 O N 4 Cl S 2
		+0.1 / +0.0	12.0	C 24 H 30 O N 2 Cl 2 S 2

Figure S33: Mass Spectrum of **BTT-7**

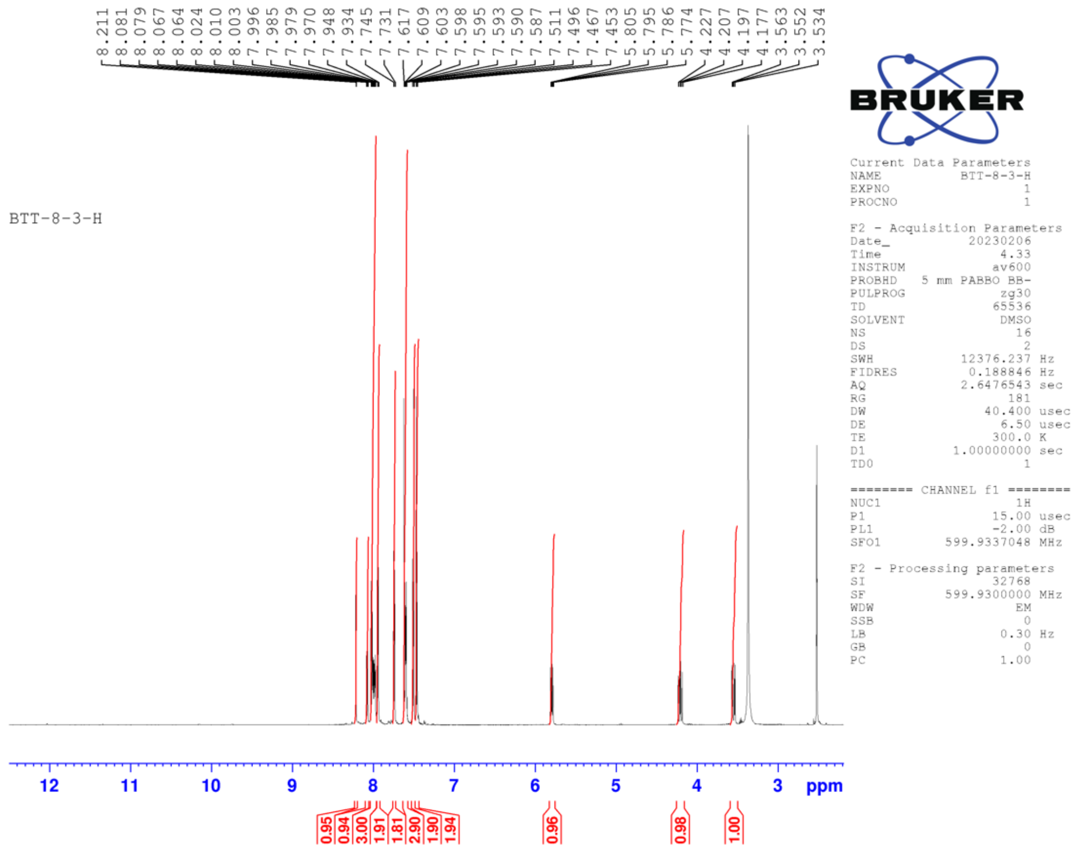

Figure S34: ^{1}H NMR Spectrum of **BTT-8**

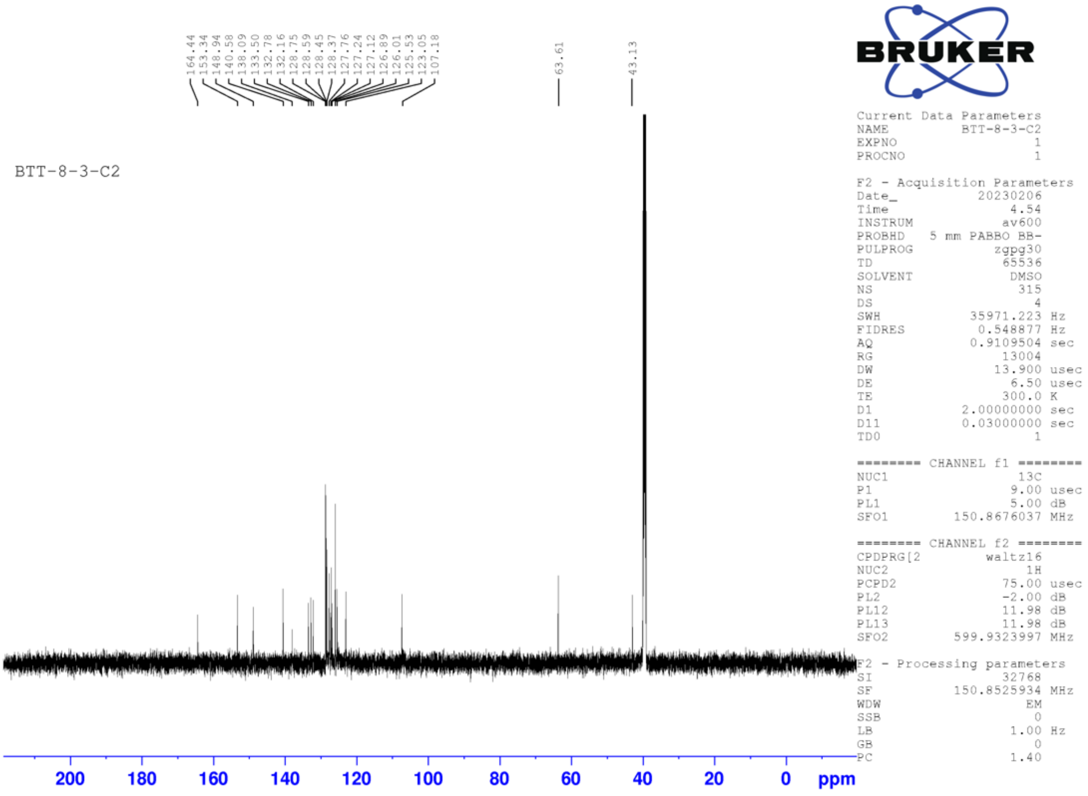

Figure S35: ^{13}C NMR Spectrum of **BTT-8**

[Elemental Composition] Page: 1
Data : gousei243 Date : 01-Feb-2023 15:54
Sample: BTT-8/sever belgin
Note : NBA
Inlet : Direct Ion Mode : FAB+
RT : 1.75 min Scan#: (6,10)
Elements : C 100/0, H 100/0, N 4/2, Cl 2/0, F 4/2, S 2/0
Mass Tolerance : 20ppm, 10mmu if m/z < 500, 20mmu if m/z > 1000
Unsaturation (U.S.) : -0.5 - 150.0

Observed m/z	Int%	Err[ppm / mmu]	U.S.	Composition
533.0923	20.1	+6.1 / +3.3	33.5	C 38 H 11 N 2 F 2
		-17.1 / -9.1	29.5	C 34 H 12 N 4 F 3
		+3.0 / +1.6	25.0	C 32 H 15 N 3 Cl F 3
		+0.0 / +0.0	16.5	C 26 H 19 N 4 Cl 2 F 4
		-0.2 / -0.1	29.5	C 35 H 15 N 2 F 2 S
		-3.3 / -1.7	21.0	C 29 H 19 N 3 Cl F 3 S
		+16.9 / +9.0	16.5	C 27 H 22 N 2 Cl 2 F 3 S
		-6.3 / -3.4	12.5	C 23 H 23 N 4 Cl 2 F 4 S
		-6.5 / -3.5	25.5	C 32 H 19 N 2 F 2 S 2
		+17.0 / +9.1	26.0	C 31 H 17 N 3 F 2 S 2
		-9.6 / -5.1	17.0	C 26 H 23 N 3 Cl F 3 S 2
		+14.0 / +7.5	17.5	C 25 H 21 N 4 Cl F 3 S 2
		+10.6 / +5.6	12.5	C 24 H 26 N 2 Cl 2 F 3 S 2
		-12.7 / -6.7	8.5	C 20 H 27 N 4 Cl 2 F 4 S 2
534.1013	17.6	+8.2 / +4.4	33.0	C 38 H 12 N 2 F 2
		-14.9 / -8.0	29.0	C 34 H 13 N 4 F 3
		-18.4 / -9.8	24.0	C 33 H 18 N 2 Cl F 3
		+5.2 / +2.8	24.5	C 32 H 16 N 3 Cl F 3
		+2.1 / +1.1	16.0	C 26 H 20 N 4 Cl 2 F 4
		+1.9 / +1.0	29.0	C 35 H 16 N 2 F 2 S
		-1.1 / -0.6	20.5	C 29 H 20 N 3 Cl F 3 S
		+19.0 / +10.1	16.0	C 27 H 23 N 2 Cl 2 F 3 S
		-4.2 / -2.2	12.0	C 23 H 24 N 4 Cl 2 F 4 S
		-4.4 / -2.3	25.0	C 32 H 20 N 2 F 2 S 2
		+19.2 / +10.2	25.5	C 31 H 18 N 3 F 2 S 2
		-7.4 / -4.0	16.5	C 26 H 24 N 3 Cl F 3 S 2
		+16.1 / +8.6	17.0	C 25 H 22 N 4 Cl F 3 S 2
		+12.7 / +6.8	12.0	C 24 H 27 N 2 Cl 2 F 3 S 2
		-10.5 / -5.6	8.0	C 20 H 28 N 4 Cl 2 F 4 S 2

Figure S36: Mass Spectrum of **BTT-8**

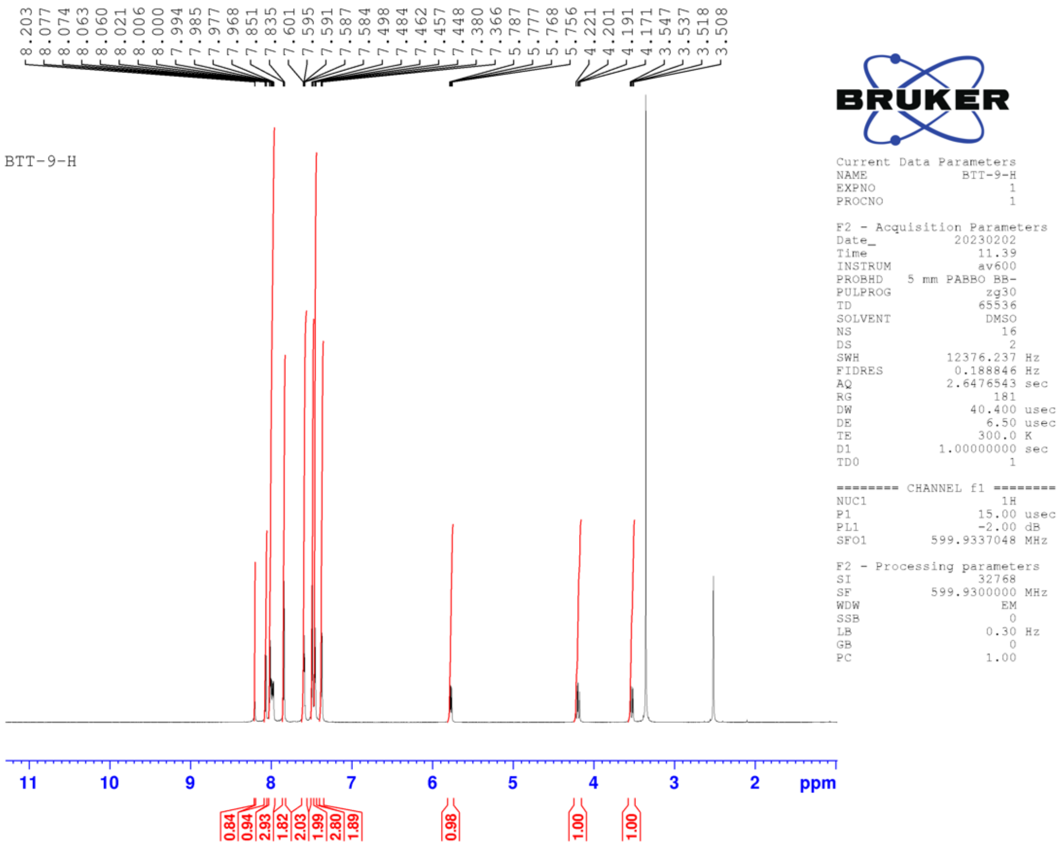

Figure S37: ^{1}H NMR Spectrum of **BTT-9**

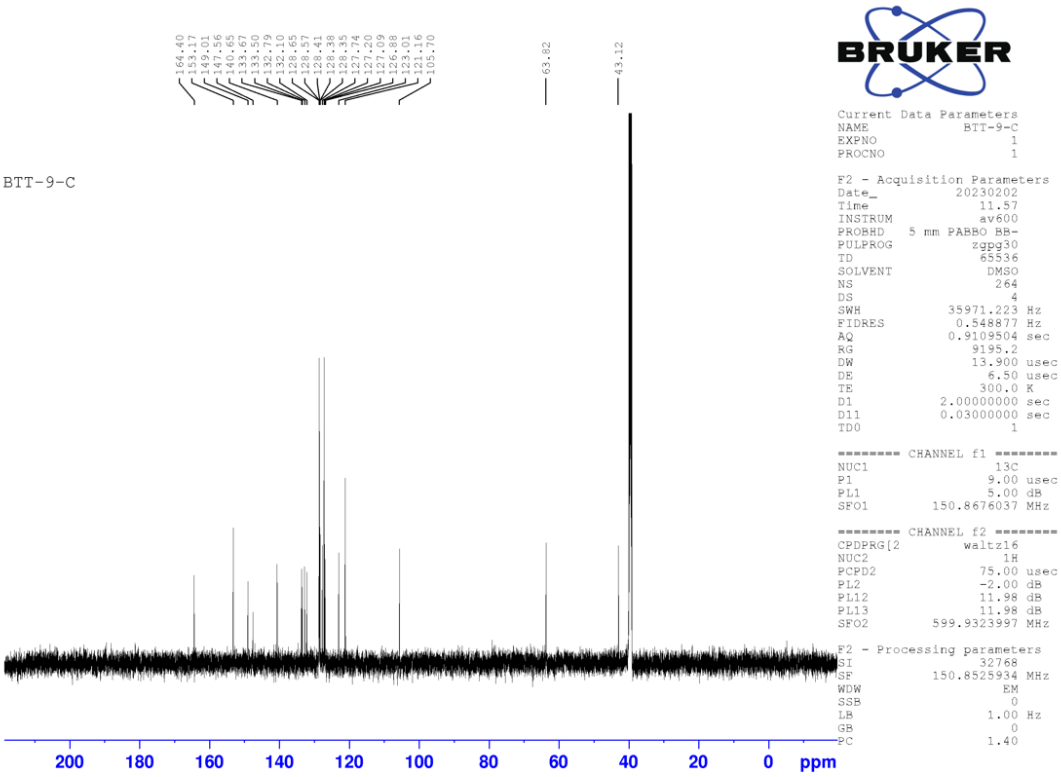

Figure S38: ^{13}C NMR Spectrum of **BTT-9**

[Elemental Composition] Page: 1

Data : gousei246 Date : 03-Feb-2023 14:45

Sample: BTT-9/sever belgin

Note : NBA

Inlet : Direct Ion Mode : FAB+

RT : 0.88 min Scan#: (2,7)

Elements : C 100/0, H 100/0, O 2/0, N 4/2, Cl 2/0, F 4/2, S 2/0

Mass Tolerance : 20ppm, 10mmu if m/z < 500, 20mmu if m/z > 1000

Unsaturation (U.S.) : -0.5 - 150.0

Observed m/z	Int%	Err[ppm / mmu]	U.S.	Composition
549.0903	100.0	+11.6 / +6.4	33.5	C 38 H 11 O N 2 F 2
		-8.8 / -4.8	33.5	C 37 H 11 N 4 F 2
		-12.2 / -6.7	28.5	C 36 H 16 N 2 Cl F 2
		+10.7 / +5.9	29.0	C 35 H 14 N 3 Cl F 2
		-4.8 / -2.7	24.5	C 31 H 16 O 2 N 4 Cl F 2
		-8.2 / -4.5	19.5	C 30 H 21 O 2 N 2 Cl 2 F 2
		+14.7 / +8.1	20.0	C 29 H 19 O 2 N 3 Cl 2 F 2
		+9.6 / +5.2	29.5	C 35 H 12 O 2 N 2 F 3
		-10.9 / -6.0	29.5	C 34 H 12 O N 4 F 3
		-14.2 / -7.8	24.5	C 33 H 17 O N 2 Cl F 3
		+8.7 / +4.8	25.0	C 32 H 15 O N 3 Cl F 3
		-15.1 / -8.3	20.0	C 30 H 20 N 3 Cl 2 F 3
		+7.8 / +4.3	20.5	C 29 H 18 N 4 Cl 2 F 3
		+2.6 / +1.4	30.0	C 35 H 11 N 3 F 4
		-13.0 / -7.1	25.5	C 31 H 13 O 2 N 4 F 4
		-16.3 / -9.0	20.5	C 30 H 18 O 2 N 2 Cl F 4
		+6.6 / +3.6	21.0	C 29 H 16 O 2 N 3 Cl F 4
		-17.2 / -9.4	16.0	C 27 H 21 O N 3 Cl 2 F 4
		+5.7 / +3.1	16.5	C 26 H 19 O N 4 Cl 2 F 4
		+5.5 / +3.0	29.5	C 35 H 15 O N 2 F 2 S
		-15.0 / -8.2	29.5	C 34 H 15 N 4 F 2 S
		-18.3 / -10.0	24.5	C 33 H 20 N 2 Cl F 2 S
		+4.6 / +2.5	25.0	C 32 H 18 N 3 Cl F 2 S
		-11.0 / -6.0	20.5	C 28 H 20 O 2 N 4 Cl F 2 S
		-14.3 / -7.9	15.5	C 27 H 25 O 2 N 2 Cl 2 F 2 S
		+8.6 / +4.7	16.0	C 26 H 23 O 2 N 3 Cl 2 F 2 S
		+3.4 / +1.9	25.5	C 32 H 16 O 2 N 2 F 3 S
		-17.0 / -9.4	25.5	C 31 H 16 O N 4 F 3 S
		+2.5 / +1.4	21.0	C 29 H 19 O N 3 Cl F 3 S
		+1.6 / +0.9	16.5	C 26 H 22 N 4 Cl 2 F 3 S
		-3.6 / -2.0	26.0	C 32 H 15 N 3 F 4 S
		+19.4 / +10.6	26.5	C 31 H 13 N 4 F 4 S
		-19.1 / -10.5	21.5	C 28 H 17 O 2 N 4 F 4 S
		+16.0 / +8.8	21.5	C 30 H 18 N 2 Cl F 4 S
		+0.4 / +0.2	17.0	C 26 H 20 O 2 N 3 Cl F 4 S
		-0.4 / -0.2	12.5	C 23 H 23 O N 4 Cl 2 F 4 S
		-0.6 / -0.4	25.5	C 32 H 19 O N 2 F 2 S 2
		-1.5 / -0.8	21.0	C 29 H 22 N 3 Cl F 2 S 2
		-17.1 / -9.4	16.5	C 25 H 24 O 2 N 4 Cl F 2 S 2
		+18.0 / +9.9	16.5	C 27 H 25 N 2 Cl 2 F 2 S 2
		+2.5 / +1.3	12.0	C 23 H 27 O 2 N 3 Cl 2 F 2 S 2
		-2.7 / -1.5	21.5	C 29 H 20 O 2 N 2 F 3 S 2
		-3.6 / -2.0	17.0	C 26 H 23 O N 3 Cl F 3 S 2
		+19.3 / +10.6	17.5	C 25 H 21 O N 4 Cl F 3 S 2
		+16.0 / +8.8	12.5	C 24 H 26 O N 2 Cl 2 F 3 S 2
		-4.5 / -2.5	12.5	C 23 H 26 N 4 Cl 2 F 3 S 2
		-9.7 / -5.3	22.0	C 29 H 19 N 3 F 4 S 2
		+13.2 / +7.3	22.5	C 28 H 17 N 4 F 4 S 2
		+9.9 / +5.4	17.5	C 27 H 22 N 2 Cl F 4 S 2
		-5.7 / -3.1	13.0	C 23 H 24 O 2 N 3 Cl F 4 S 2
		+17.2 / +9.4	13.5	C 22 H 22 O 2 N 4 Cl F 4 S 2
		+13.9 / +7.6	8.5	C 21 H 27 O 2 N 2 Cl 2 F 4 S 2
		-6.6 / -3.6	8.5	C 20 H 27 O N 4 Cl 2 F 4 S 2

Figure S39: Mass Spectrum of **BTT-9**

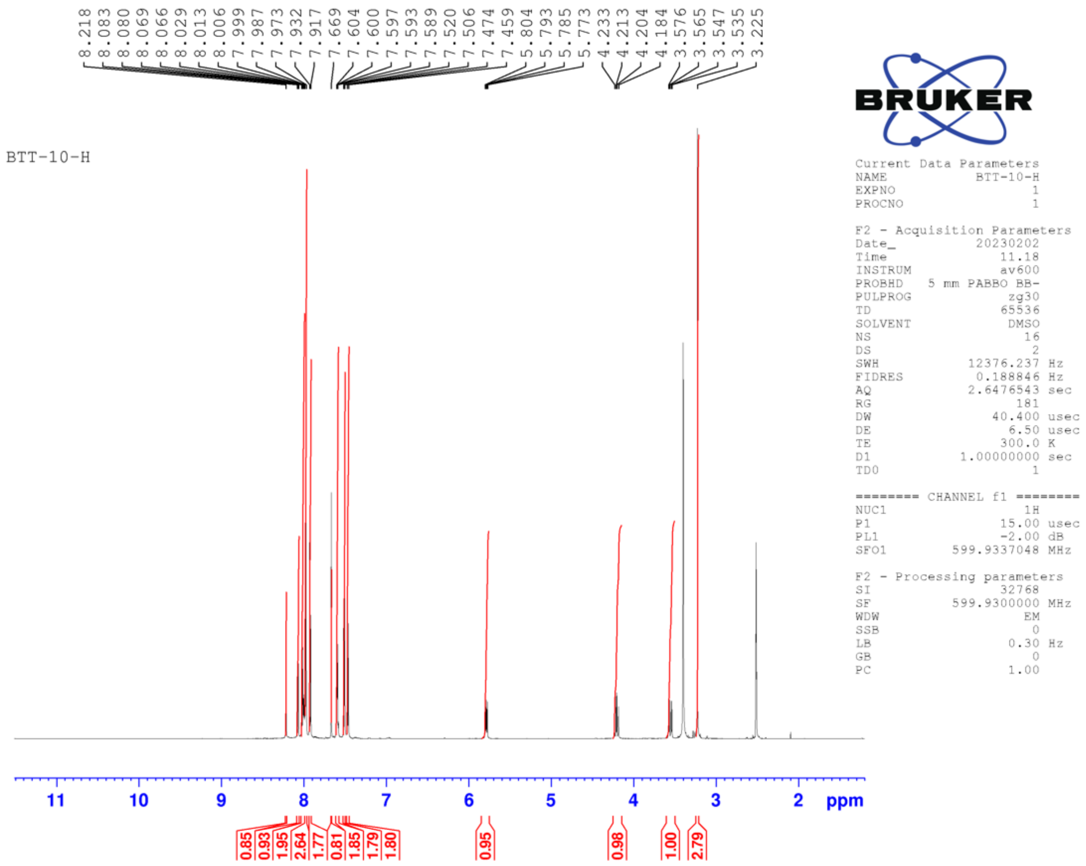

Figure S40: ^{1}H NMR Spectrum of **BTT-10**

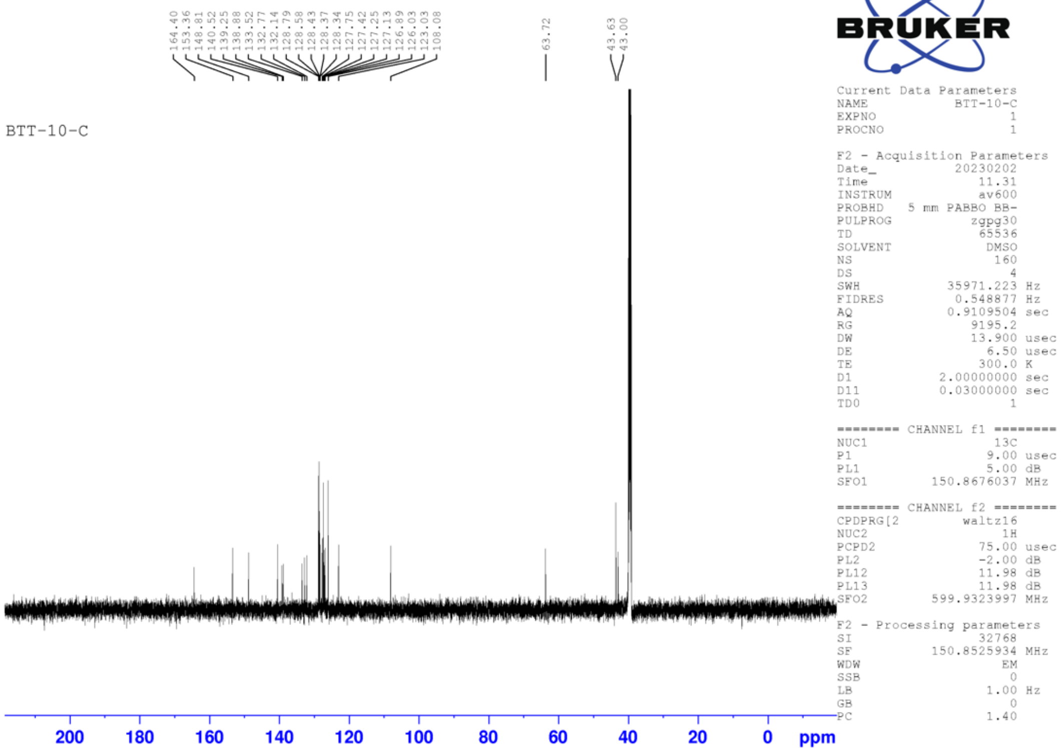

Figure S41: ^{13}C NMR Spectrum of **BTT-10**

[Elemental Composition] Page: 1

Data : gousei247 Date : 03-Feb-2023 15:10

Sample: BTT-10/sever belgin

Note : NBA

Inlet : Direct Ion Mode : FAB+

RT : 1.25 min Scan#: (3,9)

Elements : C 100/0, H 100/0, O 3/1, N 4/2, Cl 2/0, S 3/1

Mass Tolerance : 20ppm, 10mmu if m/z < 500, 20mmu if m/z > 1000

Unsaturation (U.S.) : -0.5 - 150.0

Observed m/z	Int%	Err[ppm / mmu]	U.S.	Composition
543.0842	52.2	+7.0 / +3.8	30.5	C 35 H 15 O 3 N 2 S
		-13.6 / -7.4	30.5	C 34 H 15 O 2 N 4 S
		-17.0 / -9.2	25.5	C 33 H 20 O 2 N 2 Cl S
		+6.2 / +3.3	26.0	C 32 H 18 O 2 N 3 Cl S
		-17.9 / -9.7	21.0	C 30 H 23 O N 3 Cl 2 S
		+5.3 / +2.9	21.5	C 29 H 21 O N 4 Cl 2 S
		+0.8 / +0.5	26.5	C 32 H 19 O 3 N 2 S 2
		-19.8 / -10.8	26.5	C 31 H 19 O 2 N 4 S 2
		-0.1 / +0.0	22.0	C 29 H 22 O 2 N 3 Cl S 2
		+19.7 / +10.7	17.5	C 27 H 25 O 2 N 2 Cl 2 S 2
		-1.0 / -0.5	17.5	C 26 H 25 O N 4 Cl 2 S 2
		-5.4 / -2.9	22.5	C 29 H 23 O 3 N 2 S 3
		+17.8 / +9.7	23.0	C 28 H 21 O 3 N 3 S 3
		-6.3 / -3.4	18.0	C 26 H 26 O 2 N 3 Cl S 3
		+16.9 / +9.2	18.5	C 25 H 24 O 2 N 4 Cl S 3
		+13.5 / +7.3	13.5	C 24 H 29 O 2 N 2 Cl 2 S 3
		-7.2 / -3.9	13.5	C 23 H 29 O N 4 Cl 2 S 3

Figure S42: Mass Spectrum of **BTT-10**

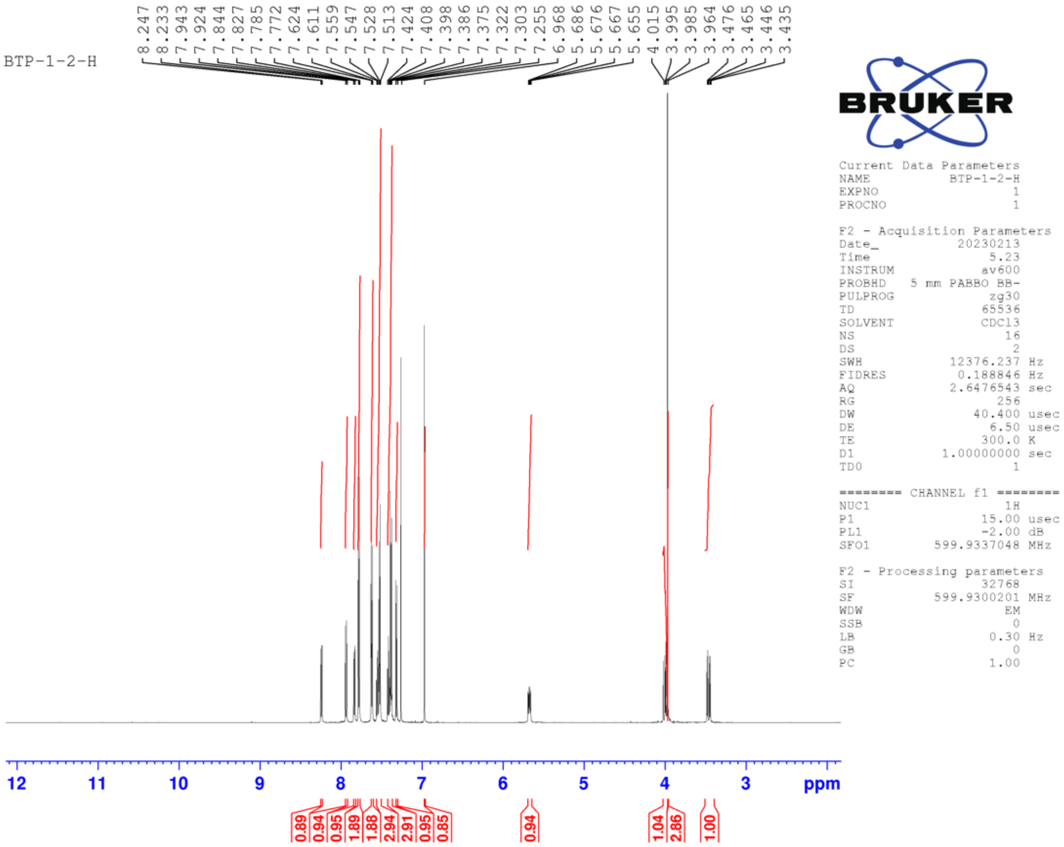

Figure S43: ^{1}H NMR Spectrum of **BTP-1**

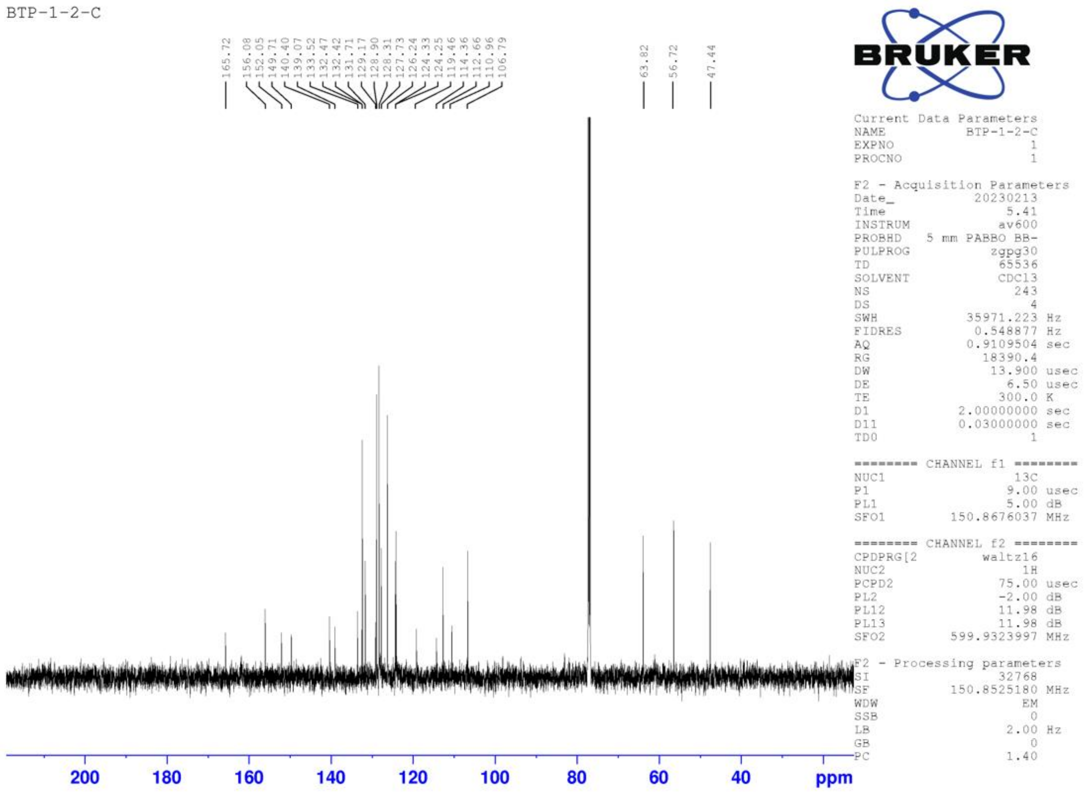

Figure S44: ^{13}C NMR Spectrum of **BTP-1**

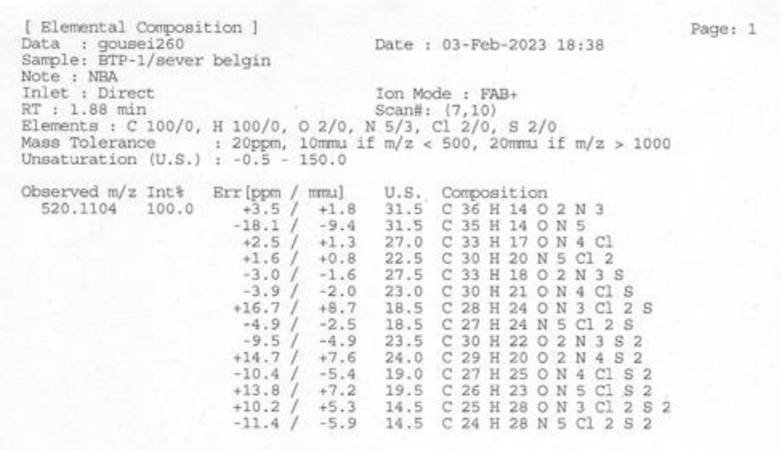

```
[ Elemental Composition ]                                        Page: 1
Data   : gousei260                    Date : 03-Feb-2023 18:38
Sample: BTP-1/sever belgin
Note : NBA
Inlet : Direct                        Ion Mode : FAB+
RT : 1.88 min                         Scan#: (7,10)
Elements : C 100/0, H 100/0, O 2/0, N 5/3, Cl 2/0, S 2/0
Mass Tolerance      : 20ppm, 10mmu if m/z < 500, 20mmu if m/z > 1000
Unsaturation (U.S.) : -0.5 - 150.0

Observed m/z Int%   Err[ppm / mmu]    U.S.  Composition
  520.1104   100.0     +3.5 /  +1.8   31.5  C 36 H 14 O 2 N 3
                      -18.1 /  -9.4   31.5  C 35 H 14 O N 5
                       +2.5 /  +1.3   27.0  C 33 H 17 O N 4 Cl
                       +1.6 /  +0.8   22.5  C 30 H 20 N 5 Cl 2
                       -3.0 /  -1.6   27.5  C 33 H 18 O 2 N 3 S
                       -3.9 /  -2.0   23.0  C 30 H 21 O N 4 Cl S
                      +16.7 /  +8.7   18.5  C 28 H 24 O N 3 Cl 2 S
                       -4.9 /  -2.5   18.5  C 27 H 24 N 5 Cl 2 S
                       -9.5 /  -4.9   23.5  C 30 H 22 O 2 N 3 S 2
                      +14.7 /  +7.6   24.0  C 29 H 20 O 2 N 4 S 2
                      -10.4 /  -5.4   19.0  C 27 H 25 O N 4 Cl S 2
                      +13.8 /  +7.2   19.5  C 26 H 23 O N 5 Cl S 2
                      +10.2 /  +5.3   14.5  C 25 H 28 O N 3 Cl 2 S 2
                      -11.4 /  -5.9   14.5  C 24 H 28 N 5 Cl 2 S 2
```

Figure S45: Mass Spectrum of **BTP-1**

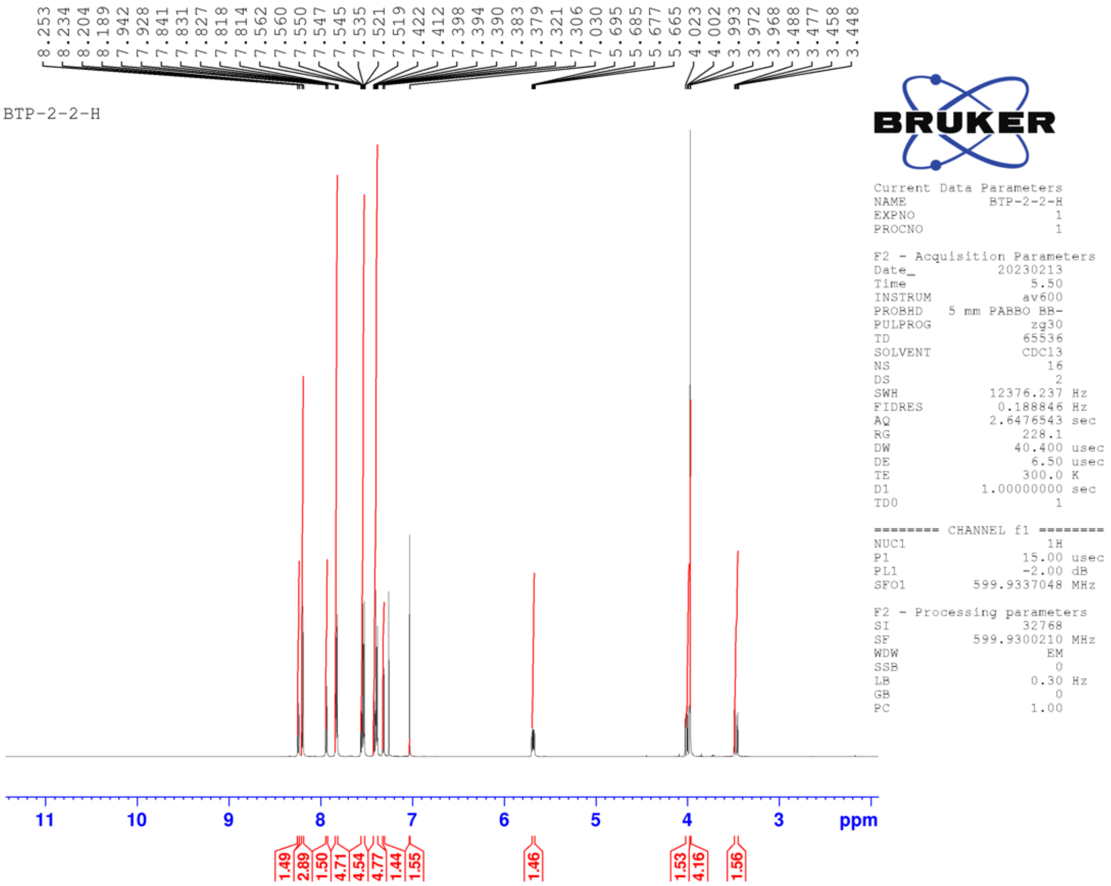

Figure S46: ^{1}H NMR Spectrum of **BTP-2**

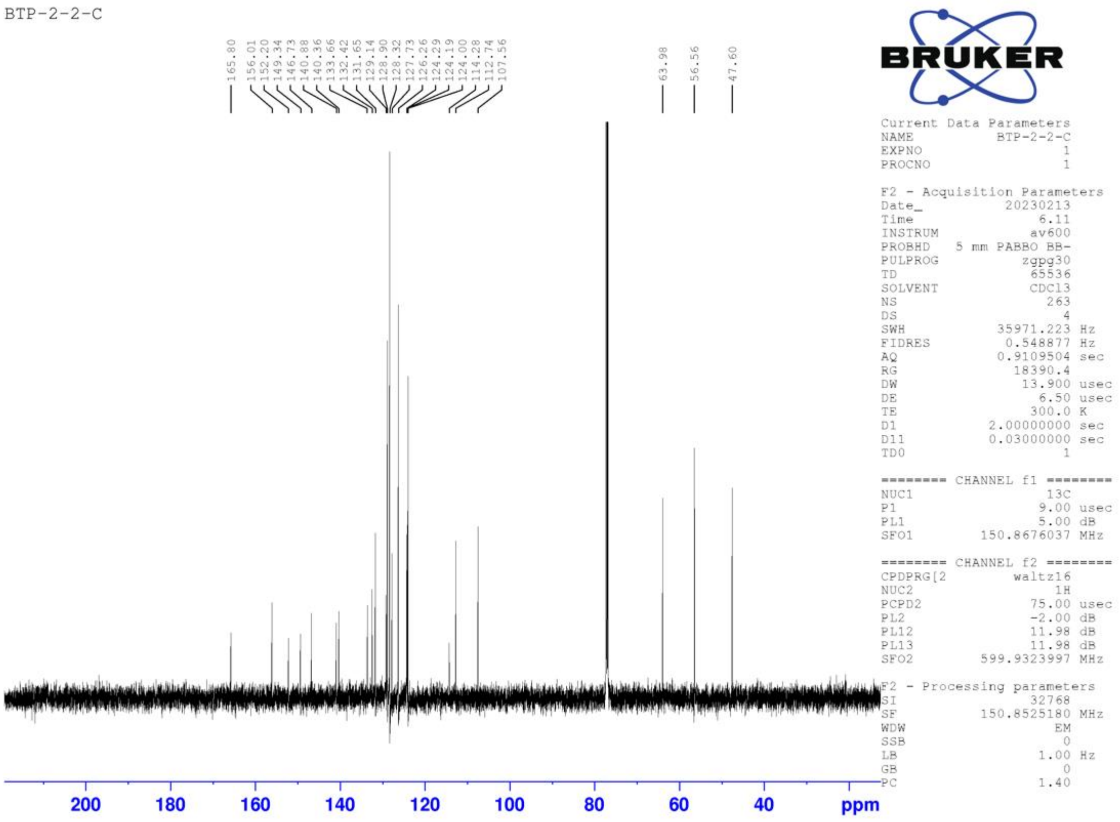

Figure S47: ^{13}C NMR Spectrum of **BTP-2**

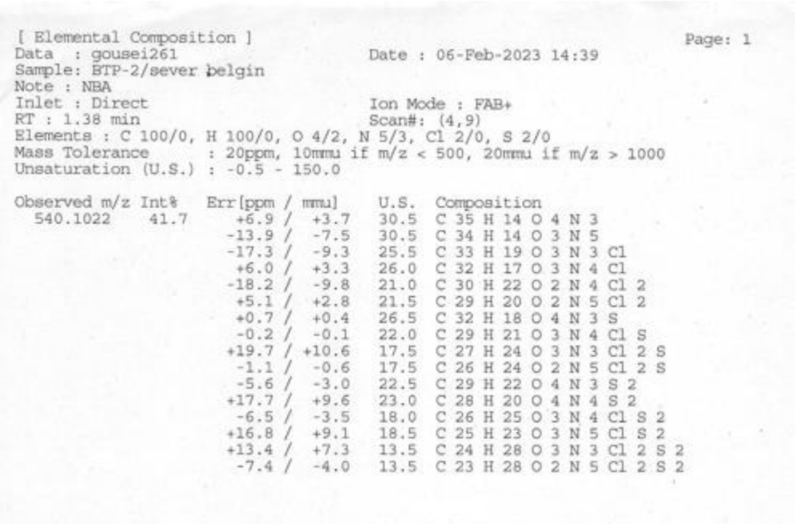

[Elemental Composition] Page: 1
Data : gousei261 Date : 06-Feb-2023 14:39
Sample: BTP-2/sever belgin
Note : NBA
Inlet : Direct Ion Mode : FAB+
RT : 1.38 min Scan#: (4,9)
Elements : C 100/0, H 100/0, O 4/2, N 5/3, Cl 2/0, S 2/0
Mass Tolerance : 20ppm, 10mmu if m/z < 500, 20mmu if m/z > 1000
Unsaturation (U.S.) : -0.5 - 150.0

Observed m/z	Int%	Err[ppm / mmu]	U.S.	Composition
540.1022	41.7	+6.9 / +3.7	30.5	C 35 H 14 O 4 N 3
		-13.9 / -7.5	30.5	C 34 H 14 O 3 N 5
		-17.3 / -9.3	25.5	C 33 H 19 O 3 N 3 Cl
		+6.0 / +3.3	26.0	C 32 H 17 O 3 N 4 Cl
		-18.2 / -9.8	21.0	C 30 H 22 O 2 N 4 Cl 2
		+5.1 / +2.8	21.5	C 29 H 20 O 2 N 5 Cl 2
		+0.7 / +0.4	26.5	C 32 H 18 O 4 N 3 S
		-0.2 / -0.1	22.0	C 29 H 21 O 3 N 4 Cl S
		+19.7 / +10.6	17.5	C 27 H 24 O 3 N 3 Cl 2 S
		-1.1 / -0.6	17.5	C 26 H 24 O 2 N 5 Cl 2 S
		-5.6 / -3.0	22.5	C 29 H 22 O 4 N 3 S 2
		+17.7 / +9.6	23.0	C 28 H 20 O 4 N 4 S 2
		-6.5 / -3.5	18.0	C 26 H 25 O 3 N 4 Cl S 2
		+16.8 / +9.1	18.5	C 25 H 23 O 3 N 5 Cl S 2
		+13.4 / +7.3	13.5	C 24 H 28 O 3 N 3 Cl 2 S 2
		-7.4 / -4.0	13.5	C 23 H 28 O 2 N 5 Cl 2 S 2

Figure S48: Mass Spectrum of **BTP-2**

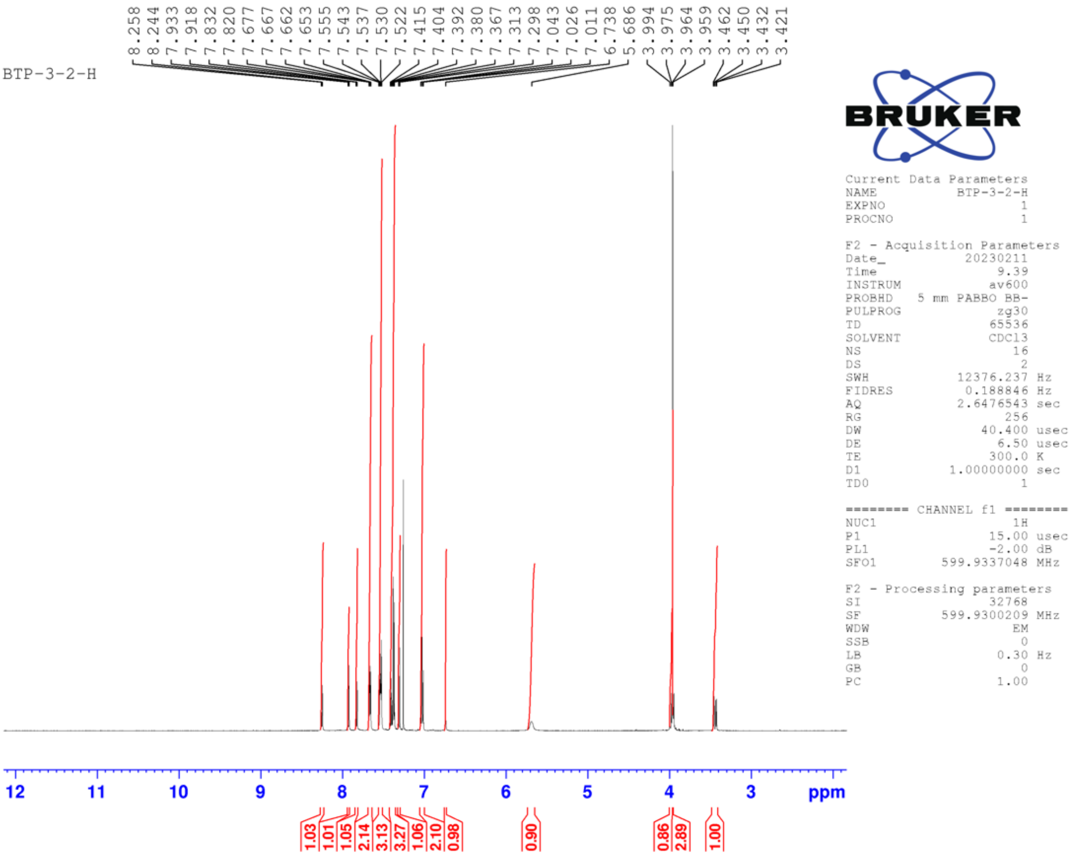

Figure S49: ^{1}H NMR Spectrum of **BTP-3**

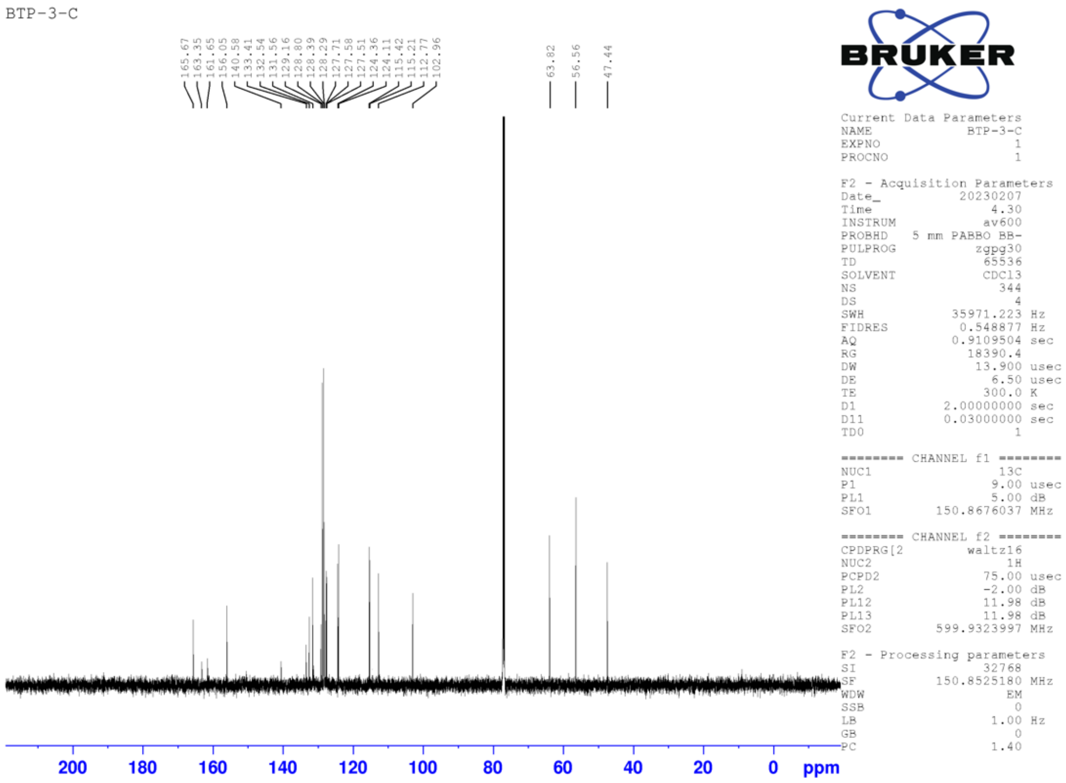

Figure S50: ^{13}C NMR Spectrum of **BTP-3**

[Elemental Composition] Page: 1

Data : gousei264 Date : 07-Feb-2023 15:45

Sample: BTP-3/sever belgin

Note : NBA

Inlet : Direct Ion Mode : FAB+

RT : 1.63 min Scan#: (5,10)

Elements : C 100/0, H 100/0, O 2/0, N 4/2, Cl 2/0, F 2/0, S 2/0

Mass Tolerance : 20ppm, 10mmu if m/z < 500, 20mmu if m/z > 1000

Unsaturation (U.S.) : -0.5 - 150.0

Observed m/z	Int%	Err[ppm / mmu]	U.S.	Composition
513.1071	100.0	+8.3 / +4.3	33.5	C 38 H 13 O N 2
		-13.6 / -7.0	33.5	C 37 H 13 N 4
		-17.1 / -8.8	28.5	C 36 H 18 N 2 Cl
		+7.4 / +3.8	29.0	C 35 H 16 N 3 Cl
		-9.3 / -4.8	24.5	C 31 H 18 O 2 N 4 Cl
		-12.9 / -6.6	19.5	C 30 H 23 O 2 N 2 Cl 2
		+11.6 / +6.0	20.0	C 29 H 21 O 2 N 3 Cl 2
		+6.1 / +3.1	29.5	C 35 H 14 O 2 N 2 F
		-15.8 / -8.1	29.5	C 34 H 14 O N 4 F
		-19.4 / -9.9	24.5	C 33 H 19 O N 2 Cl F
		+5.1 / +2.6	25.0	C 32 H 17 O N 3 Cl F
		+4.2 / +2.2	20.5	C 29 H 20 N 4 Cl 2 F
		-1.4 / -0.7	30.0	C 35 H 13 N 3 F 2
		-18.0 / -9.3	25.5	C 31 H 15 O 2 N 4 F 2
		+19.6 / +10.0	25.5	C 33 H 16 N 2 Cl F 2
		+2.9 / +1.5	21.0	C 29 H 18 O 2 N 3 Cl F 2
		+2.0 / +1.0	16.5	C 26 H 21 O N 4 Cl 2 F 2
		+1.7 / +0.9	29.5	C 35 H 17 O N 2 S
		+0.8 / +0.4	25.0	C 32 H 20 N 3 Cl S
		-15.9 / -8.1	20.5	C 28 H 22 O 2 N 4 Cl S
		-19.4 / -10.0	15.5	C 27 H 27 O 2 N 2 Cl 2 S
		+5.1 / +2.6	16.0	C 26 H 25 O 2 N 3 Cl 2 S
		-0.5 / -0.2	25.5	C 32 H 18 O 2 N 2 F S
		-1.4 / -0.7	21.0	C 29 H 21 O N 3 Cl F S
		+19.5 / +10.0	16.5	C 27 H 24 O N 2 Cl 2 F S
		-2.4 / -1.2	16.5	C 26 H 24 N 4 Cl 2 F S
		-7.9 / -4.1	26.0	C 32 H 17 N 3 F 2 S
		+16.6 / +8.5	26.5	C 31 H 15 N 4 F 2 S
		+13.0 / +6.7	21.5	C 30 H 20 N 2 Cl F 2 S
		-3.7 / -1.9	17.0	C 26 H 22 O 2 N 3 Cl F 2 S
		+17.3 / +8.9	12.5	C 24 H 25 O 2 N 2 Cl 2 F 2 S
		-4.6 / -2.4	12.5	C 23 H 25 O N 4 Cl 2 F 2 S
		-4.8 / -2.5	25.5	C 32 H 21 O N 2 S 2
		+19.7 / +10.1	26.0	C 31 H 19 O N 3 S 2
		-5.8 / -3.0	21.0	C 29 H 24 N 3 Cl S 2
		+18.7 / +9.6	21.5	C 28 H 22 N 4 Cl S 2
		+15.2 / +7.8	16.5	C 27 H 27 N 2 Cl 2 S 2
		-1.5 / -0.8	12.0	C 23 H 29 O 2 N 3 Cl 2 S 2
		-7.1 / -3.6	21.5	C 29 H 22 O 2 N 2 F S 2
		+17.5 / +9.0	22.0	C 28 H 20 O 2 N 3 F S 2
		-8.0 / -4.1	17.0	C 26 H 25 O N 3 Cl F S 2
		+16.5 / +8.5	17.5	C 25 H 23 O N 4 Cl F S 2
		+12.9 / +6.6	12.5	C 24 H 28 O N 2 Cl 2 F S 2
		-9.0 / -4.6	12.5	C 23 H 28 N 4 Cl 2 F S 2
		-14.5 / -7.4	22.0	C 29 H 21 N 3 F 2 S 2
		+10.0 / +5.1	22.5	C 28 H 19 N 4 F 2 S 2
		+6.4 / +3.3	17.5	C 27 H 24 N 2 Cl F 2 S 2
		-10.2 / -5.2	13.0	C 23 H 26 O 2 N 3 Cl F 2 S 2
		+14.3 / +7.3	13.5	C 22 H 24 O 2 N 4 Cl F 2 S 2
		+10.7 / +5.5	8.5	C 21 H 29 O 2 N 2 Cl 2 F 2 S 2
		-11.2 / -5.7	8.5	C 20 H 29 O N 4 Cl 2 F 2 S 2

Figure S51: Mass Spectrum of **BTP-3**

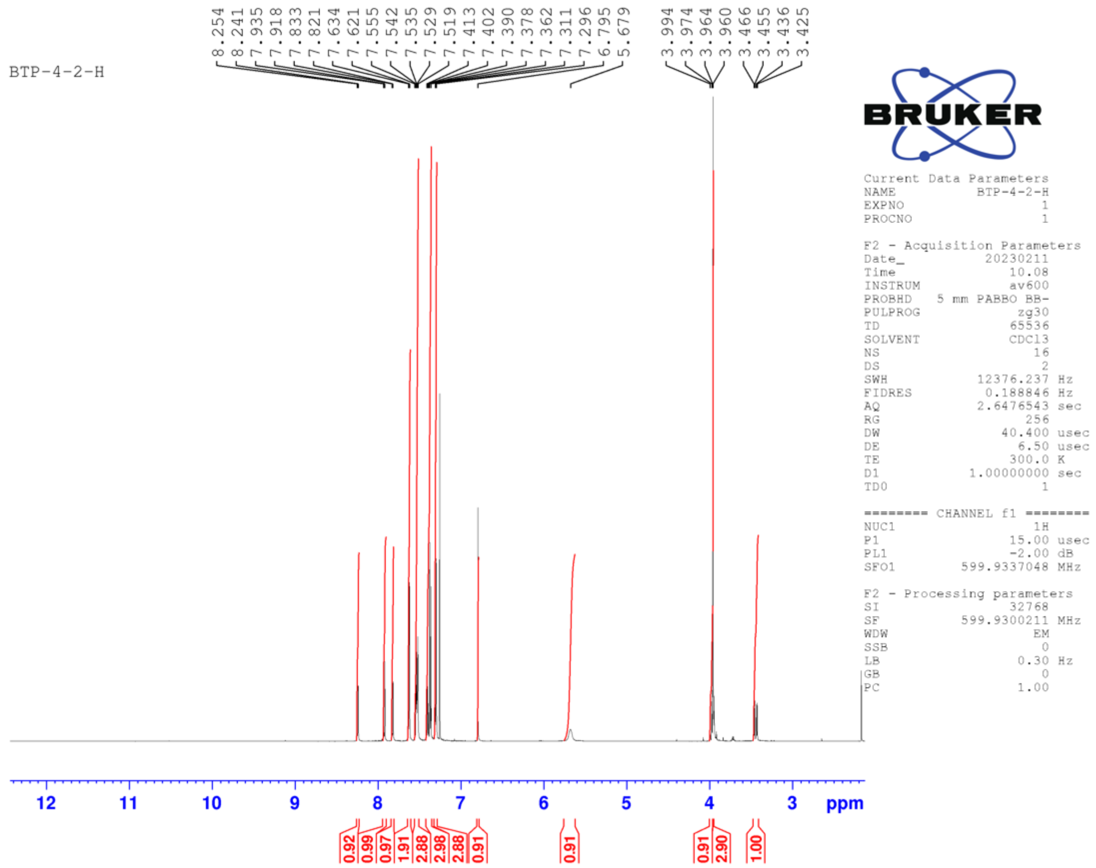

Figure S52: ^{1}H NMR Spectrum of **BTP-4**

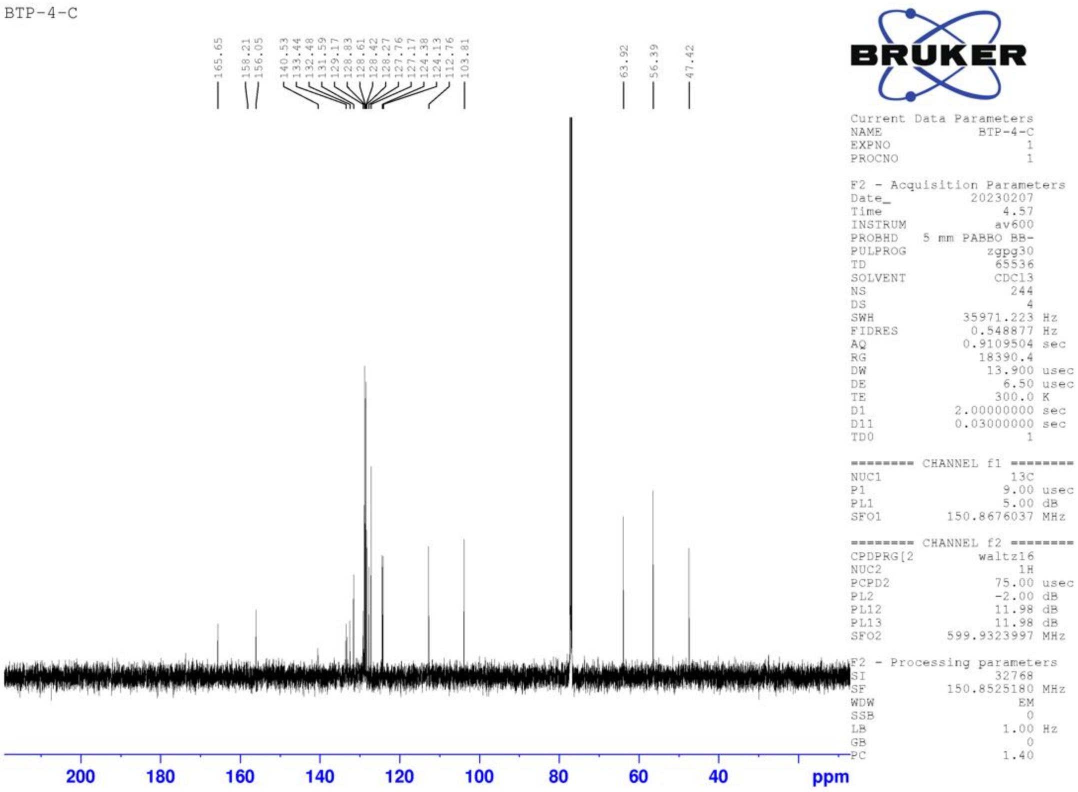

Figure S53: ^{13}C NMR Spectrum of **BTP-4**

[Elemental Composition] Page: 1
Data : gousei265 Date : 07-Feb-2023 16:00
Sample: BTP-4/sever belgin
Note : NBA
Inlet : Direct Ion Mode : FAB+
RT : 0.75 min Scan#: (2,6)
Elements : C 100/0, H 100/0, O 2/0, N 4/2, Cl 3/1, S 2/0
Mass Tolerance : 20ppm, 10mmu if m/z < 500, 20mmu if m/z > 1000
Unsaturation (U.S.) : -0.5 - 150.0

Observed m/z	Int%	Err[ppm / mmu]	U.S.	Composition
529.0782	100.0	+7.3 / +3.9	29.5	C 35 H 14 O 2 N 2 Cl
		-13.9 / -7.4	29.5	C 34 H 14 O N 4 Cl
		-17.4 / -9.2	24.5	C 33 H 19 O N 2 Cl 2
		+6.4 / +3.4	25.0	C 32 H 17 O N 3 Cl 2
		-18.3 / -9.7	20.0	C 30 H 22 N 3 Cl 3
		+5.5 / +2.9	20.5	C 29 H 20 N 4 Cl 3
		+0.9 / +0.5	25.5	C 32 H 18 O 2 N 2 Cl S
		+0.0 / +0.0	21.0	C 29 H 21 O N 3 Cl 2 S
		-0.9 / -0.5	16.5	C 26 H 24 N 4 Cl 3 S
		-5.4 / -2.9	21.5	C 29 H 22 O 2 N 2 Cl S 2
		+18.3 / +9.7	22.0	C 28 H 20 O 2 N 3 Cl S 2
		-6.4 / -3.4	17.0	C 26 H 25 O N 3 Cl 2 S 2
		+17.4 / +9.2	17.5	C 25 H 23 O N 4 Cl 2 S 2
		+14.0 / +7.4	12.5	C 24 H 28 O N 2 Cl 3 S 2
		-7.3 / -3.9	12.5	C 23 H 28 N 4 Cl 3 S 2

Figure S54: Mass Spectrum of **BTP-4**

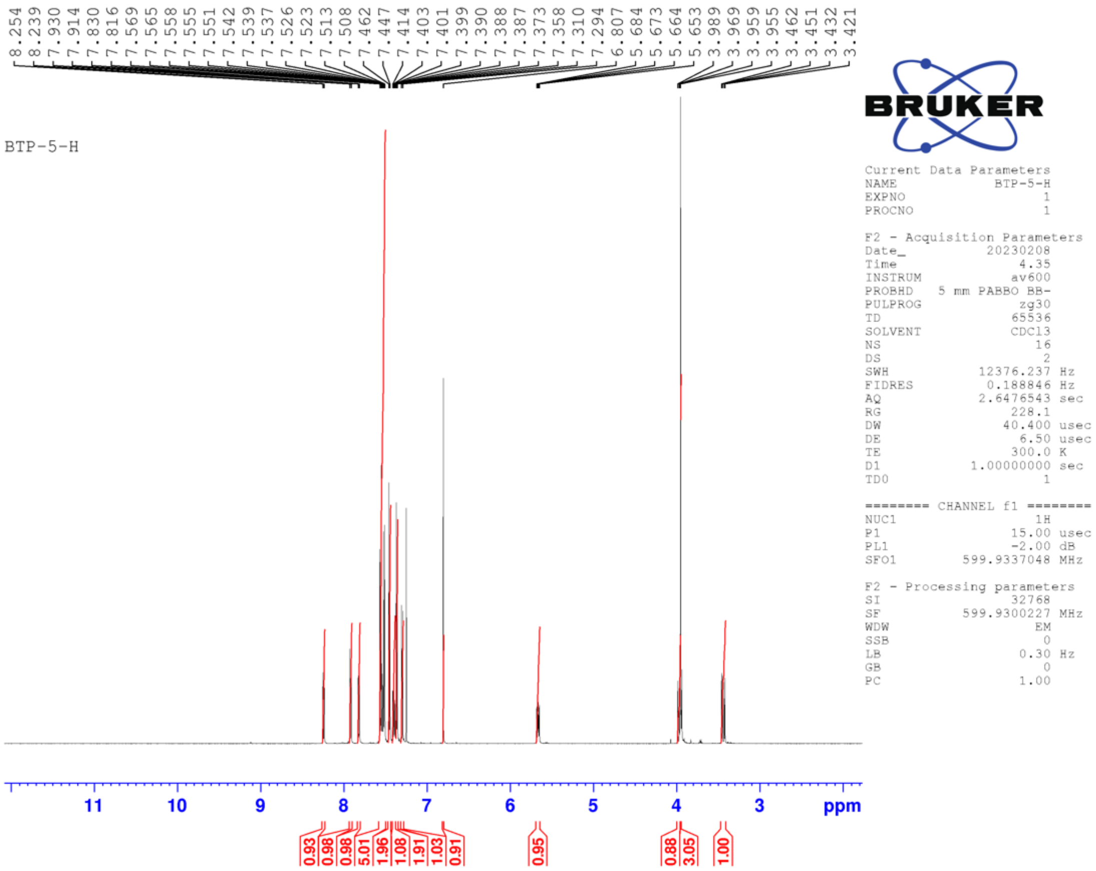

Figure S55: ^{1}H NMR Spectrum of **BTP-5**

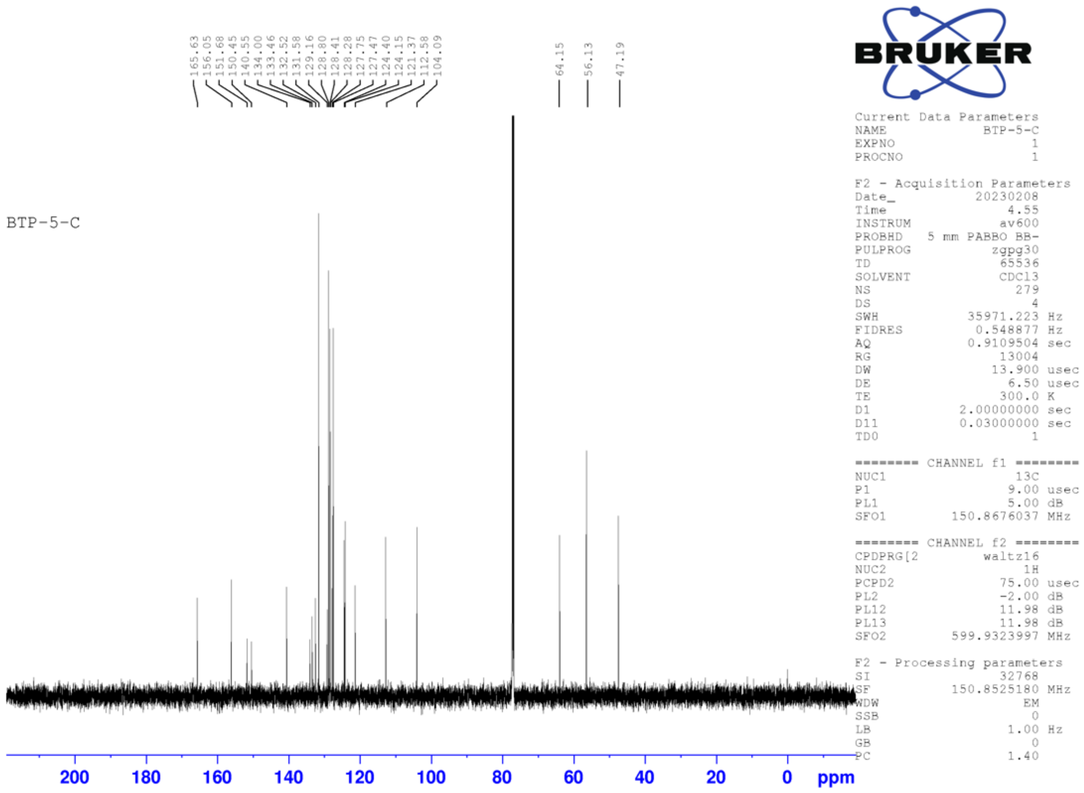

Figure S56: ^{13}C NMR Spectrum of **BTP-5**

[Elemental Composition] Page: 1

Data : gousei266 Date : 08-Feb-2023 16:20

Sample: BTP-5/sever belgin

Note : NBA

Inlet : Direct Ion Mode : FAB+

RT : 1.88 min Scan#: (7,10)

Elements : C 100/0, H 100/0, O 2/0, N 4/2, Cl 2/0, Br 2/0, S 2/0

Mass Tolerance : 20ppm, 10mmu if m/z < 500, 20mmu if m/z > 1000

Unsaturation (U.S.) : -0.5 - 150.0

Observed m/z	Int%	Err[ppm / mmu]	U.S.	Composition
573.0266	62.7	-10.7 / -6.1	45.0	C 44 H 3 N 3
		+11.2 / +6.4	45.5	C 43 H N 4
		+8.0 / +4.6	40.5	C 42 H 6 N 2 Cl
		-6.9 / -4.0	36.0	C 38 H 8 O 2 N 3 Cl
		+15.0 / +8.6	36.5	C 37 H 6 O 2 N 4 Cl
		+11.9 / +6.8	31.5	C 36 H 11 O 2 N 2 Cl 2
		-7.8 / -4.4	31.5	C 35 H 11 O N 4 Cl 2
		+4.7 / +2.7	29.5	C 35 H 14 O 2 N 2 Br
		-14.9 / -8.5	29.5	C 34 H 14 O N 4 Br
		-18.1 / -10.4	24.5	C 33 H 19 O N 2 Cl Br
		+3.8 / +2.2	25.0	C 32 H 17 O N 3 Cl Br
		-19.0 / -10.9	20.0	C 30 H 22 N 3 Cl 2 Br
		+3.0 / +1.7	20.5	C 29 H 20 N 4 Cl 2 Br
		+15.4 / +8.8	18.5	C 29 H 23 O N 2 Br 2
		-4.2 / -2.4	18.5	C 28 H 23 N 4 Br 2
		-7.4 / -4.2	13.5	C 27 H 28 N 2 Cl Br 2
		+14.6 / +8.4	14.0	C 26 H 26 N 3 Cl Br 2
		-0.4 / -0.2	9.5	C 22 H 28 O 2 N 4 Cl Br 2
		-3.5 / -2.0	4.5	C 21 H 33 O 2 N 2 Cl 2 Br 2
		+18.4 / +10.5	5.0	C 20 H 31 O 2 N 3 Cl 2 Br 2
		-16.6 / -9.5	41.0	C 41 H 7 N 3 S
		+5.3 / +3.1	41.5	C 40 H 5 N 4 S
		+2.1 / +1.2	36.5	C 39 H 10 N 2 Cl S
		-12.8 / -7.3	32.0	C 35 H 12 O 2 N 3 Cl S
		+9.2 / +5.3	32.5	C 34 H 10 O 2 N 4 Cl S
		+6.0 / +3.4	27.5	C 33 H 15 O 2 N 2 Cl 2 S
		-13.6 / -7.8	27.5	C 32 H 15 O N 4 Cl 2 S
		-1.2 / -0.7	25.5	C 32 H 18 O 2 N 2 Br S
		-2.0 / -1.2	21.0	C 29 H 21 O N 3 Cl Br S
		+19.9 / +11.4	21.5	C 28 H 19 O N 4 Cl Br S
		+16.7 / +9.6	16.5	C 27 H 24 O N 2 Cl 2 Br S
		-2.9 / -1.7	16.5	C 26 H 24 N 4 Cl 2 Br S
		+9.5 / +5.5	14.5	C 26 H 27 O N 2 Br 2 S
		-10.1 / -5.8	14.5	C 25 H 27 N 4 Br 2 S
		-13.3 / -7.6	9.5	C 24 H 32 N 2 Cl Br 2 S
		+8.7 / +5.0	10.0	C 23 H 30 N 3 Cl Br 2 S
		-6.2 / -3.6	5.5	C 19 H 32 O 2 N 4 Cl Br 2 S
		-9.4 / -5.4	0.5	C 18 H 37 O 2 N 2 Cl 2 Br 2 S
		+12.5 / +7.2	1.0	C 17 H 35 O 2 N 3 Cl 2 Br 2 S
		+19.1 / +10.9	37.5	C 38 H 9 O N 2 S 2
		-0.5 / -0.3	37.5	C 37 H 9 N 4 S 2
		-3.7 / -2.1	32.5	C 36 H 14 N 2 Cl S 2
		+18.2 / +10.4	33.0	C 35 H 12 N 3 Cl S 2
		-18.7 / -10.7	28.0	C 32 H 16 O 2 N 3 Cl S 2
		+3.3 / +1.9	28.5	C 31 H 14 O 2 N 4 Cl S 2
		+0.1 / +0.0	23.5	C 30 H 19 O 2 N 2 Cl 2 S 2
		-19.5 / -11.2	23.5	C 29 H 19 O N 4 Cl 2 S 2
		-7.1 / -4.1	21.5	C 29 H 22 O 2 N 2 Br S 2
		+14.9 / +8.5	22.0	C 28 H 20 O 2 N 3 Br S 2
		-7.9 / -4.5	17.0	C 26 H 25 O N 3 Cl Br S 2
		+14.0 / +8.0	17.5	C 25 H 23 O N 4 Cl Br S 2
		+10.8 / +6.2	12.5	C 24 H 28 O N 2 Cl 2 Br S 2
		-8.8 / -5.0	12.5	C 23 H 28 N 4 Cl 2 Br S 2
		+3.7 / +2.1	10.5	C 23 H 31 O N 2 Br 2 S 2
		-15.9 / -9.1	10.5	C 22 H 31 N 4 Br 2 S 2

Figure S57: Mass Spectrum of **BTP-5**

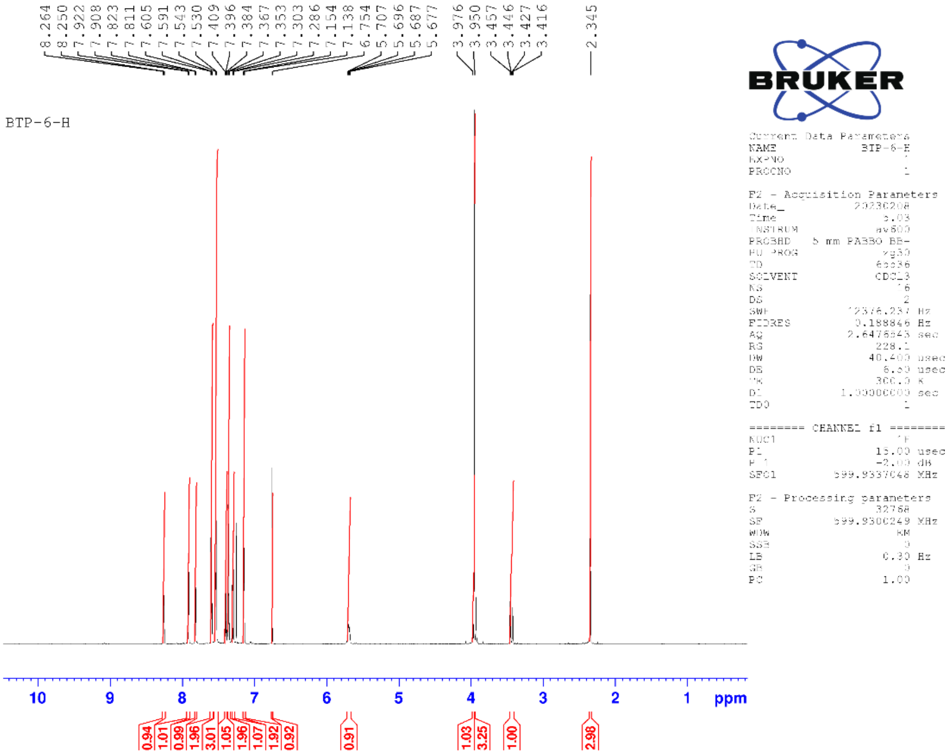

Figure S58: ^{1}H NMR Spectrum of **BTP-6**

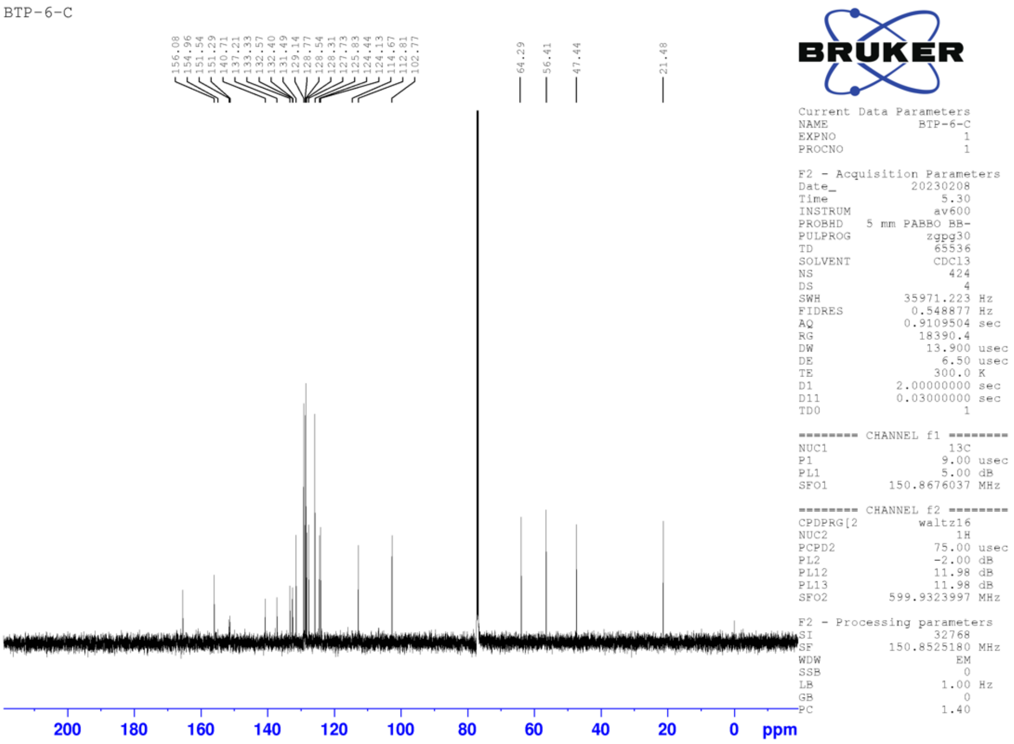

Figure S59: ^{13}C NMR Spectrum of **BTP-6**

[Elemental Composition] Page: 1
Data : gousei267 Date : 08-Feb-2023 16:38
Sample: BTP-6/sever belgin
Note : NBA
Inlet : Direct Ion Mode : FAB+
RT : 1.38 min Scan#: (5,8)
Elements : C 100/0, H 100/0, O 2/0, N 4/2, Cl 2/0, S 2/0
Mass Tolerance : 20ppm, 10mmu if m/z < 500, 20mmu if m/z > 1000
Unsaturation (U.S.) : -0.5 - 150.0

Observed m/z	Int%	Err [ppm / mmu]	U.S.	Composition
509.1328	100.0	+7.4 / +3.7	29.5	C 36 H 17 O 2 N 2
		-14.7 / -7.5	29.5	C 35 H 17 O N 4
		-18.3 / -9.3	24.5	C 34 H 22 O N 2 Cl
		+6.4 / +3.3	25.0	C 33 H 20 O N 3 Cl
		-19.3 / -9.8	20.0	C 31 H 25 N 3 Cl 2
		+5.4 / +2.8	20.5	C 30 H 23 N 4 Cl 2
		+0.7 / +0.4	25.5	C 33 H 21 O 2 N 2 S
		-0.2 / -0.1	21.0	C 30 H 24 O N 3 Cl S
		-1.2 / -0.6	16.5	C 27 H 27 N 4 Cl 2 S
		-5.9 / -3.0	21.5	C 30 H 25 O 2 N 2 S 2
		+18.8 / +9.6	22.0	C 29 H 23 O 2 N 3 S 2
		-6.8 / -3.5	17.0	C 27 H 28 O N 3 Cl S 2
		+17.9 / +9.1	17.5	C 26 H 26 O N 4 Cl S 2
		+14.3 / +7.3	12.5	C 25 H 31 O N 2 Cl 2 S 2
		-7.8 / -4.0	12.5	C 24 H 31 N 4 Cl 2 S 2

Figure S60: Mass Spectrum of **BTP-6**

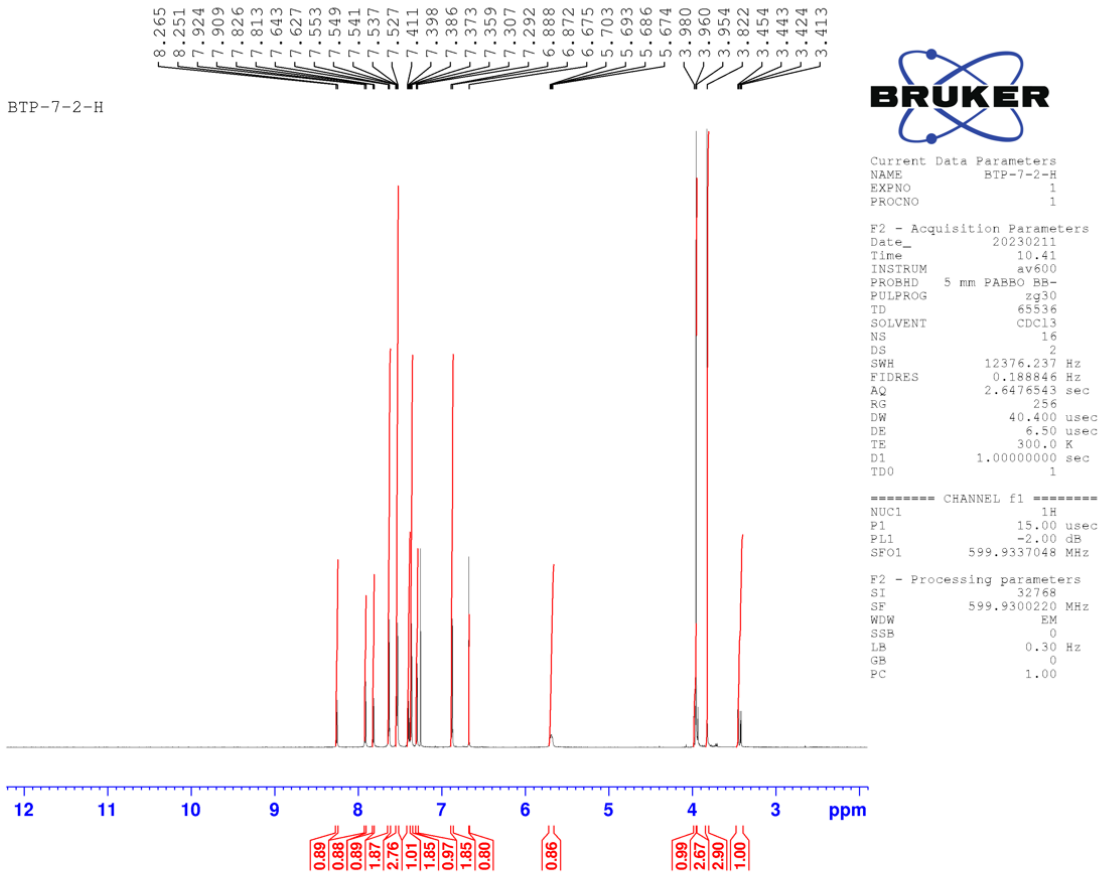

Figure S61: ^{1}H NMR Spectrum of **BTP-7**

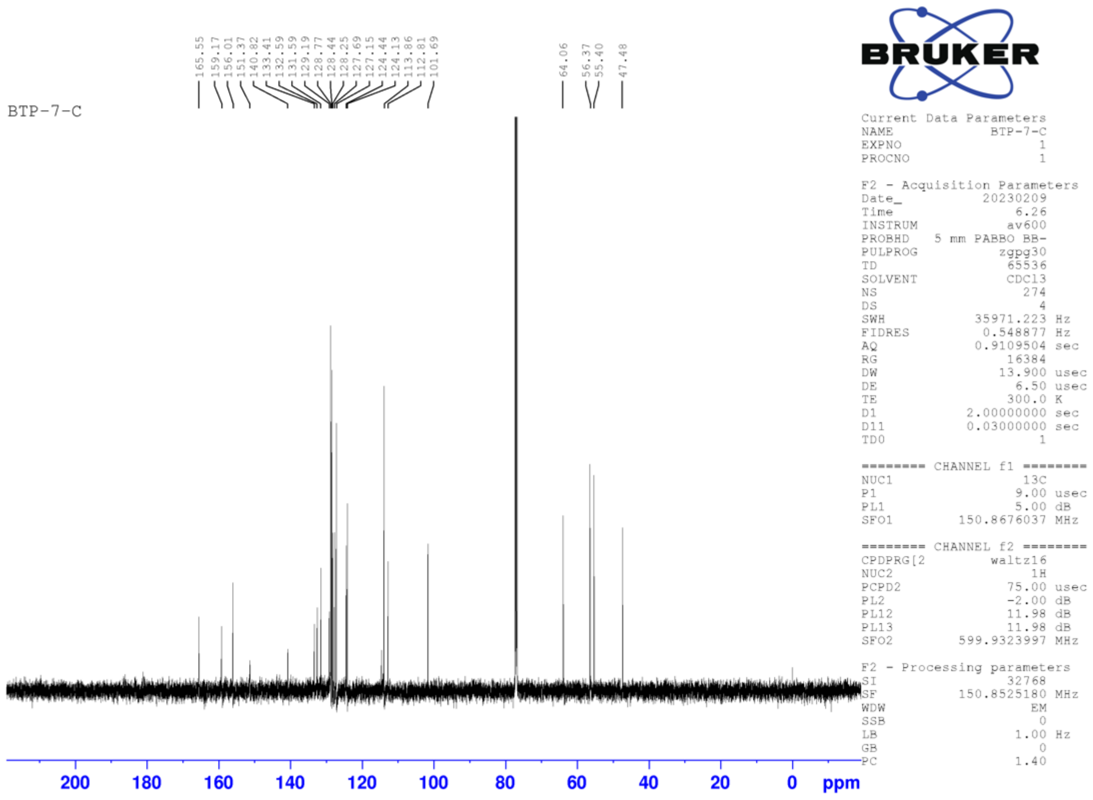

Figure S62: ^{13}C NMR Spectrum of **BTP-7**

[Elemental Composition] Page: 1
Data : gousei270 Date : 09-Feb-2023 16:23
Sample: BTP-7/sever belgin
Note : NBA
Inlet : Direct Ion Mode : FAB+
RT : 1.13 min Scan#: (3,8)
Elements : C 100/0, H 100/0, O 3/1, N 4/2, Cl 2/0, S 2/0
Mass Tolerance : 20ppm, 10mmu if m/z < 500, 20mmu if m/z > 1000
Unsaturation (U.S.) : -0.5 - 150.0

Observed m/z	Int%	Err[ppm / mmu]	U.S.	Composition
525.1281	99.6	+7.9 / +4.1	29.5	C 36 H 17 O 3 N 2
		-13.5 / -7.1	29.5	C 35 H 17 O 2 N 4
		-17.0 / -8.9	24.5	C 34 H 22 O 2 N 2 Cl
		+6.9 / +3.6	25.0	C 33 H 20 O 2 N 3 Cl
		-17.9 / -9.4	20.0	C 31 H 25 O N 3 Cl 2
		+6.0 / +3.2	20.5	C 30 H 23 O N 4 Cl 2
		+1.5 / +0.8	25.5	C 33 H 21 O 3 N 2 S
		-19.9 / -10.5	25.5	C 32 H 21 O 2 N 4 S
		+0.5 / +0.3	21.0	C 30 H 24 O 2 N 3 Cl S
		-0.4 / -0.2	16.5	C 27 H 27 O N 4 Cl 2 S
		-5.0 / -2.6	21.5	C 30 H 25 O 3 N 2 S 2
		+19.0 / +10.0	22.0	C 29 H 23 O 3 N 3 S 2
		-5.9 / -3.1	17.0	C 27 H 28 O 2 N 3 Cl S 2
		+18.1 / +9.5	17.5	C 26 H 26 O 2 N 4 Cl S 2
		+14.6 / +7.6	12.5	C 25 H 31 O 2 N 2 Cl 2 S 2
		-6.8 / -3.6	12.5	C 24 H 31 O N 4 Cl 2 S 2

Figure S63: Mass Spectrum of **BTP-7**

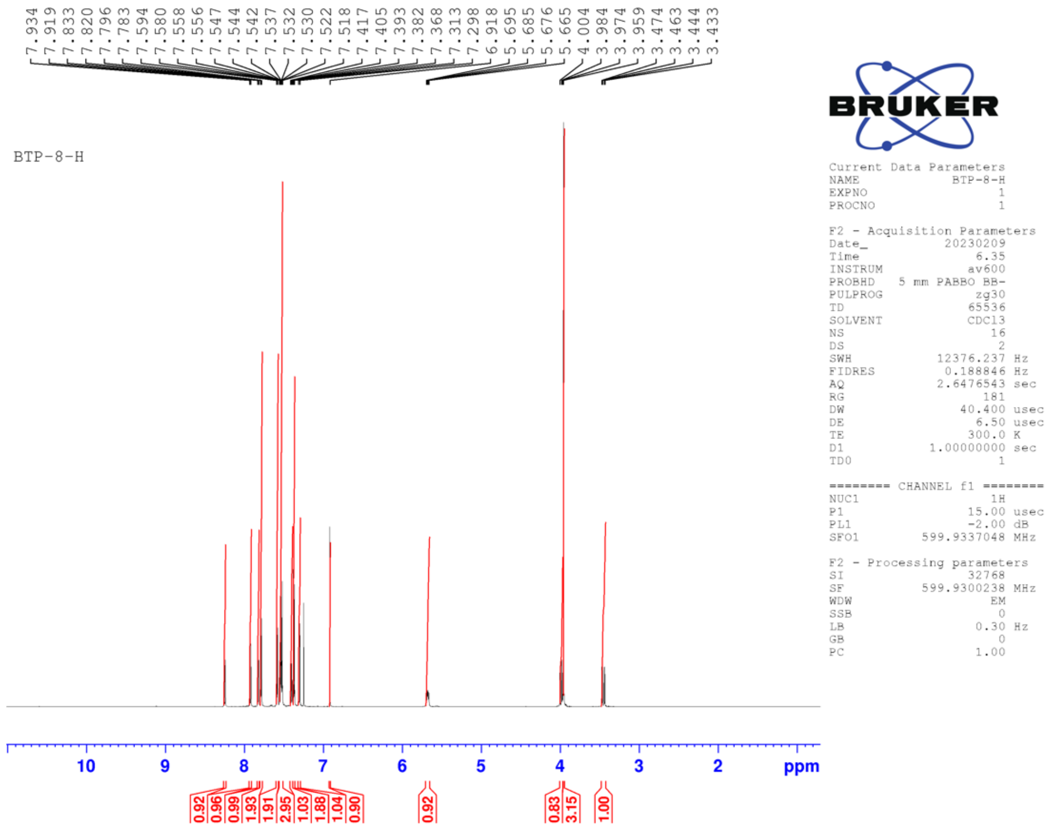

Figure S64: ^{1}H NMR Spectrum of **BTP-8**

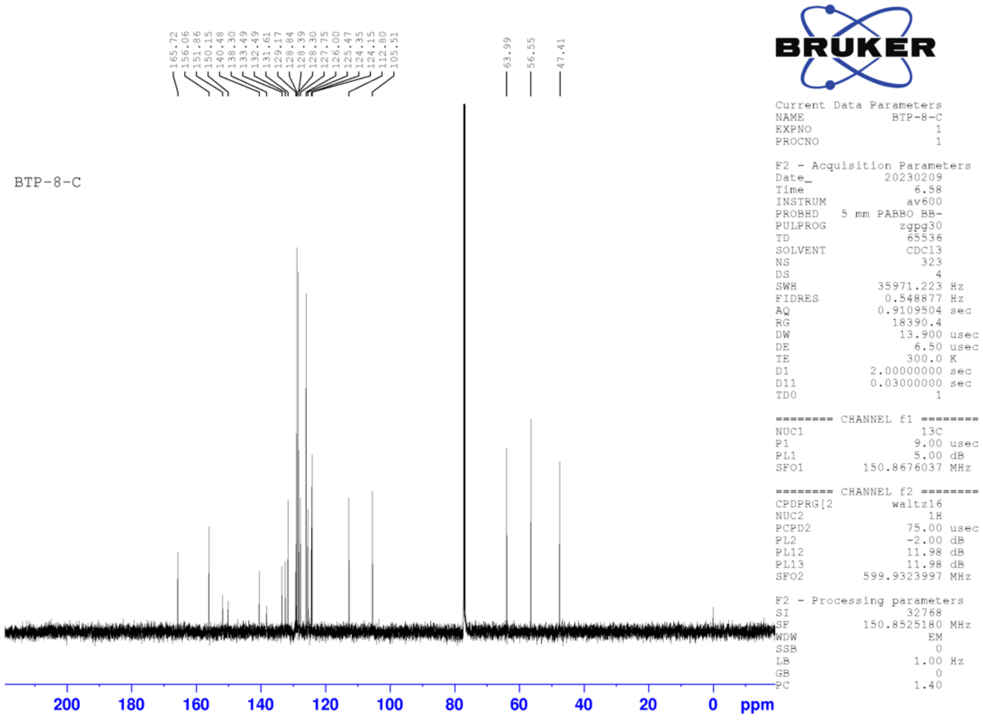

Figure S65: ^{13}C NMR Spectrum of **BTP-8**

[Elemental Composition]   Page: 1

Data : gousei271   Date : 09-Feb-2023 16:39

Sample: BTP-8/sever belgin

Note : NBA

Inlet : Direct   Ion Mode : FAB+

RT : 1.25 min   Scan#: (3,9)

Elements : C 100/0, H 100/0, O 2/0, N 4/2, Cl 2/0, F 4/2, S 2/0

Mass Tolerance : 20ppm, 10mmu if m/z < 500, 20mmu if m/z > 1000

Unsaturation (U.S.) : -0.5 - 150.0

Observed m/z	Int%	Err[ppm / mmu]	U.S.	Composition
563.1066	100.0	+12.4 / +7.0	33.5	C 39 H 13 O N 2 F 2
		-7.6 / -4.3	33.5	C 38 H 13 N 4 F 2
		-10.8 / -6.1	28.5	C 37 H 18 N 2 Cl F 2
		+11.5 / +6.5	29.0	C 36 H 16 N 3 Cl F 2
		-3.7 / -2.1	24.5	C 32 H 18 O 2 N 4 Cl F 2
		-6.9 / -3.9	19.5	C 31 H 23 O 2 N 2 Cl 2 F 2
		+15.4 / +8.7	20.0	C 30 H 21 O 2 N 3 Cl 2 F 2
		+10.4 / +5.8	29.5	C 36 H 14 O 2 N 2 F 3
		-9.6 / -5.4	29.5	C 35 H 14 O N 4 F 3
		-12.8 / -7.2	24.5	C 34 H 19 O N 2 Cl F 3
		+9.5 / +5.3	25.0	C 33 H 17 O N 3 Cl F 3
		-13.7 / -7.7	20.0	C 31 H 22 N 3 Cl 2 F 3
		+8.6 / +4.9	20.5	C 30 H 20 N 4 Cl 2 F 3
		-18.8 / -10.6	29.5	C 37 H 15 N 2 F 4
		+3.6 / +2.0	30.0	C 36 H 13 N 3 F 4
		-11.6 / -6.5	25.5	C 32 H 15 O 2 N 4 F 4
		-14.9 / -8.4	20.5	C 31 H 20 O 2 N 2 Cl F 4
		+7.5 / +4.2	21.0	C 30 H 18 O 2 N 3 Cl F 4
		-15.7 / -8.9	16.0	C 28 H 23 O N 3 Cl 2 F 4
		+6.6 / +3.7	16.5	C 27 H 21 O N 4 Cl 2 F 4
		+6.4 / +3.6	29.5	C 36 H 17 O N 2 F 2 S
		-13.6 / -7.6	29.5	C 35 H 17 N 4 F 2 S
		-16.8 / -9.5	24.5	C 34 H 22 N 2 Cl F 2 S
		+5.5 / +3.1	25.0	C 33 H 20 N 3 Cl F 2 S
		-9.7 / -5.4	20.5	C 29 H 22 O 2 N 4 Cl F 2 S
		-12.9 / -7.3	15.5	C 28 H 27 O 2 N 2 Cl 2 F 2 S
		+9.4 / +5.3	16.0	C 27 H 25 O 2 N 3 Cl 2 F 2 S
		+4.4 / +2.5	25.5	C 33 H 18 O 2 N 2 F 3 S
		-15.6 / -8.8	25.5	C 32 H 18 O N 4 F 3 S
		-18.8 / -10.6	20.5	C 31 H 23 O N 2 Cl F 3 S
		+3.5 / +2.0	21.0	C 30 H 21 O N 3 Cl F 3 S
		-19.7 / -11.1	16.0	C 28 H 26 N 3 Cl 2 F 3 S
		+2.6 / +1.5	16.5	C 27 H 24 N 4 Cl 2 F 3 S
		-2.4 / -1.4	26.0	C 33 H 17 N 3 F 4 S
		+19.9 / +11.2	26.5	C 32 H 15 N 4 F 4 S
		-17.6 / -9.9	21.5	C 29 H 19 O 2 N 4 F 4 S
		+16.7 / +9.4	21.5	C 31 H 20 N 2 Cl F 4 S
		+1.5 / +0.8	17.0	C 27 H 22 O 2 N 3 Cl F 4 S
		+0.6 / +0.3	12.5	C 24 H 25 O N 4 Cl 2 F 4 S
		+0.4 / +0.2	25.5	C 33 H 21 O N 2 F 2 S 2
		-19.5 / -11.0	25.5	C 32 H 21 N 4 F 2 S 2
		-0.5 / -0.3	21.0	C 30 H 24 N 3 Cl F 2 S 2
		-15.6 / -8.8	16.5	C 26 H 26 O 2 N 4 Cl F 2 S 2
		+18.6 / +10.5	16.5	C 28 H 27 N 2 Cl 2 F 2 S 2
		-18.9 / -10.6	11.5	C 25 H 31 O 2 N 2 Cl 2 F 2 S 2
		+3.4 / +1.9	12.0	C 24 H 29 O 2 N 3 Cl 2 F 2 S 2
		-1.6 / -0.9	21.5	C 30 H 22 O 2 N 2 F 3 S 2
		-2.5 / -1.4	17.0	C 27 H 25 O N 3 Cl F 3 S 2
		+19.8 / +11.2	17.5	C 26 H 23 O N 4 Cl F 3 S 2
		+16.6 / +9.3	12.5	C 25 H 28 O N 2 Cl 2 F 3 S 2
		-3.4 / -1.9	12.5	C 24 H 28 N 4 Cl 2 F 3 S 2
		-8.4 / -4.7	22.0	C 30 H 21 N 3 F 4 S 2
		+13.9 / +7.8	22.5	C 29 H 19 N 4 F 4 S 2
		+10.7 / +6.0	17.5	C 28 H 24 N 2 Cl F 4 S 2
		-4.5 / -2.5	13.0	C 24 H 26 O 2 N 3 Cl F 4 S 2

Figure S66: Mass Spectrum of **BTP-8**

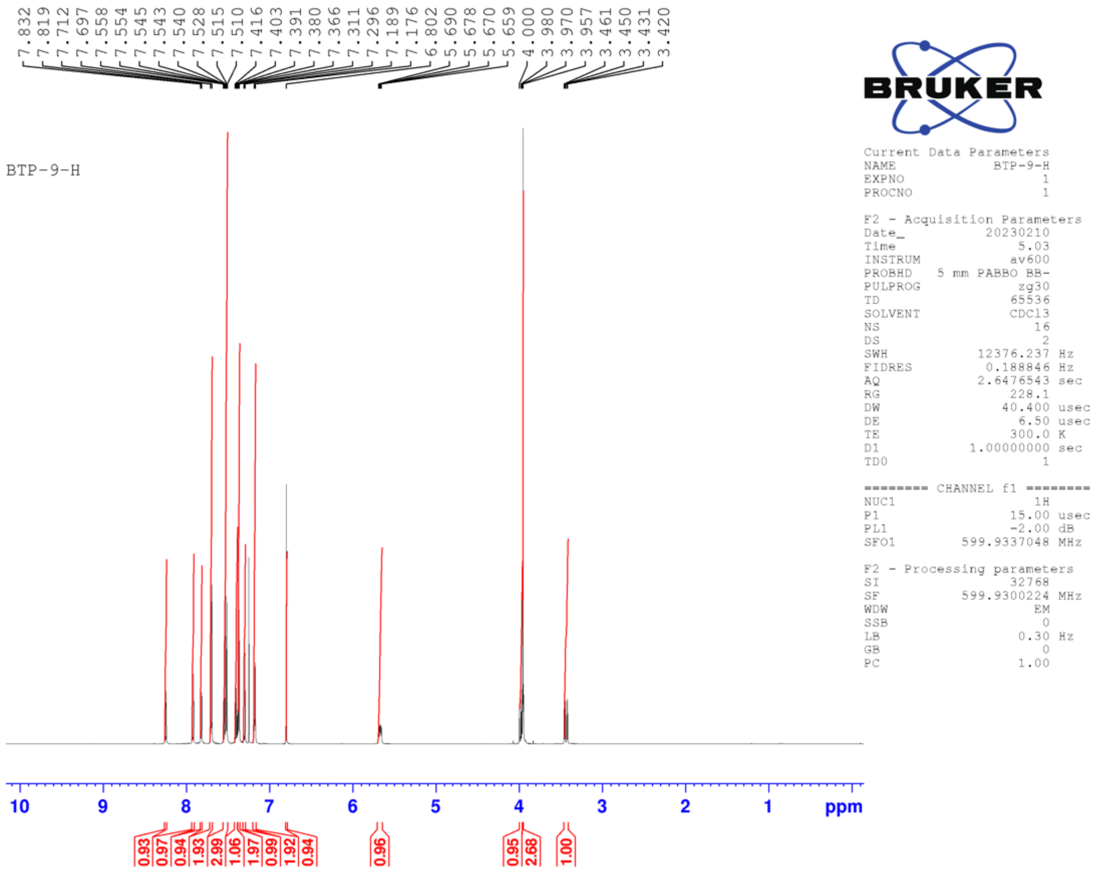

Figure S67: ^{1}H NMR Spectrum of **BTP-9**

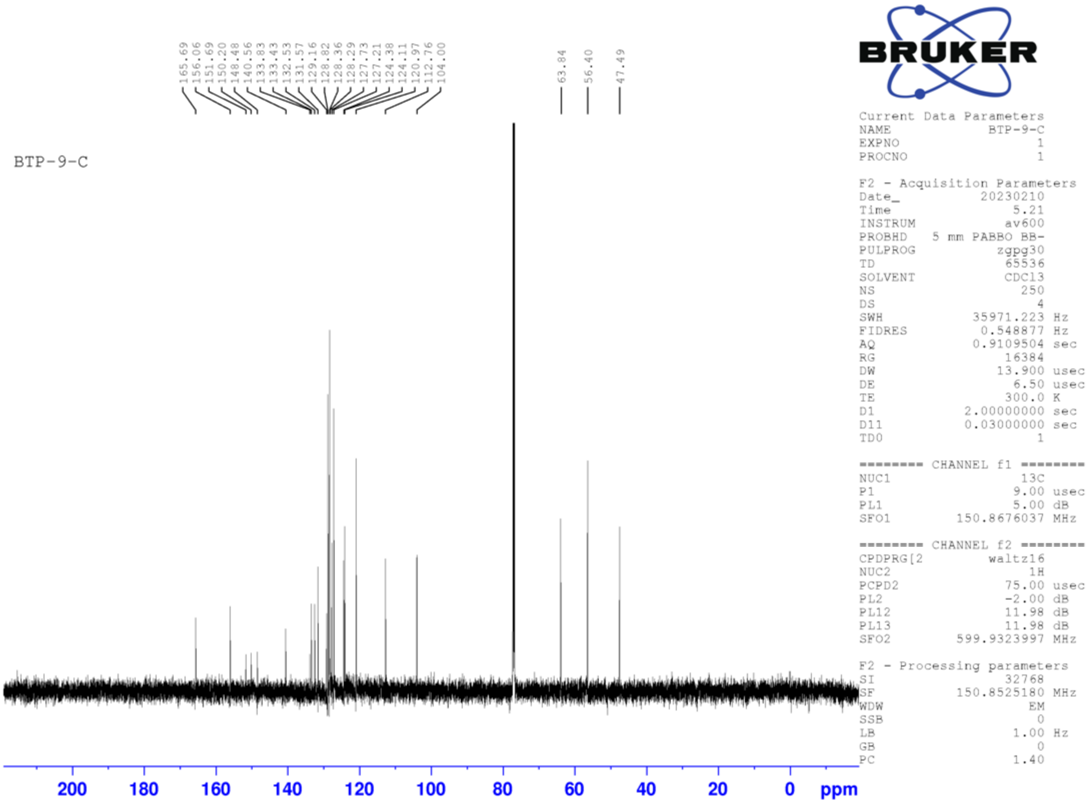

Figure S68: ^{13}C NMR Spectrum of **BTP-9**

[Elemental Composition] Page: 1

Data : gousei272 Date : 10-Feb-2023 14:15

Sample: BTP-9/sever belgin

Note : NBA

Inlet : Direct Ion Mode : FAB+

RT : 1.00 min Scan#: (2,8)

Elements : C 100/0, H 100/0, O 3/1, N 4/2, Cl 2/0, F 4/2, S 2/0

Mass Tolerance : 20ppm, 10mmu if m/z < 500, 20mmu if m/z > 1000

Unsaturation (U.S.) : -0.5 - 150.0

Observed m/z	Int%	Err[ppm / mmu]	U.S.	Composition
579.1000	100.0	+9.4 / +5.5	33.5	C 39 H 13 O 2 N 2 F 2
		-10.0 / -5.8	33.5	C 38 H 13 O N 4 F 2
		-13.1 / -7.6	28.5	C 37 H 18 O N 2 Cl F 2
		+8.6 / +5.0	29.0	C 36 H 16 O N 3 Cl F 2
		-6.2 / -3.6	24.5	C 32 H 18 O 3 N 4 Cl F 2
		-9.3 / -5.4	19.5	C 31 H 23 O 3 N 2 Cl 2 F 2
		+12.4 / +7.2	20.0	C 30 H 21 O 3 N 3 Cl 2 F 2
		+7.5 / +4.3	29.5	C 36 H 14 O 3 N 2 F 3
		-11.9 / -6.9	29.5	C 35 H 14 O 2 N 4 F 3
		-15.1 / -8.7	24.5	C 34 H 19 O 2 N 2 Cl F 3
		+6.6 / +3.8	25.0	C 33 H 17 O 2 N 3 Cl F 3
		-15.9 / -9.2	20.0	C 31 H 22 O N 3 Cl 2 F 3
		+5.8 / +3.3	20.5	C 30 H 20 O N 4 Cl 2 F 3
		+0.9 / +0.5	30.0	C 36 H 13 O N 3 F 4
		-13.9 / -8.1	25.5	C 32 H 15 O 3 N 4 F 4
		+19.4 / +11.2	25.5	C 34 H 16 O N 2 Cl F 4
		-17.1 / -9.9	20.5	C 31 H 20 O 3 N 2 Cl F 4
		+4.7 / +2.7	21.0	C 30 H 18 O 3 N 3 Cl F 4
		-17.9 / -10.4	16.0	C 28 H 23 O 2 N 3 Cl 2 F 4
		+3.8 / +2.2	16.5	C 27 H 21 O 2 N 4 Cl 2 F 4
		+3.6 / +2.1	29.5	C 36 H 17 O 2 N 2 F 2 S
		-15.8 / -9.1	29.5	C 35 H 17 O N 4 F 2 S
		-18.9 / -11.0	24.5	C 34 H 22 O N 2 Cl F 2 S
		+2.8 / +1.6	25.0	C 33 H 20 O N 3 Cl F 2 S
		-12.0 / -6.9	20.5	C 29 H 22 O 3 N 4 Cl F 2 S
		-15.2 / -8.8	15.5	C 28 H 27 O 3 N 2 Cl 2 F 2 S
		+6.6 / +3.8	16.0	C 27 H 25 O 3 N 3 Cl 2 F 2 S
		+1.6 / +1.0	25.5	C 33 H 18 O 3 N 2 F 3 S
		-17.8 / -10.3	25.5	C 32 H 18 O 2 N 4 F 3 S
		+0.8 / +0.5	21.0	C 30 H 21 O 2 N 3 Cl F 3 S
		+19.4 / +11.2	16.5	C 28 H 24 O 2 N 2 Cl 2 F 3 S
		+0.0 / +0.0	16.5	C 27 H 24 O N 4 Cl 2 F 3 S
		-5.0 / -2.9	26.0	C 33 H 17 O N 3 F 4 S
		+16.8 / +9.7	26.5	C 32 H 15 O N 4 F 4 S
		-19.7 / -11.4	21.5	C 29 H 19 O 3 N 4 F 4 S
		+13.6 / +7.9	21.5	C 31 H 20 O N 2 Cl F 4 S
		-1.2 / -0.7	17.0	C 27 H 22 O 3 N 3 Cl F 4 S
		+17.4 / +10.1	12.5	C 25 H 25 O 3 N 2 Cl 2 F 4 S
		-2.0 / -1.2	12.5	C 24 H 25 O 2 N 4 Cl 2 F 4 S
		-2.2 / -1.3	25.5	C 33 H 21 O 2 N 2 F 2 S 2
		+19.5 / +11.3	26.0	C 32 H 19 O 2 N 3 F 2 S 2
		-3.0 / -1.8	21.0	C 30 H 24 O N 3 Cl F 2 S 2
		+18.7 / +10.8	21.5	C 29 H 22 O N 4 Cl F 2 S 2
		-17.8 / -10.3	16.5	C 26 H 26 O 3 N 4 Cl F 2 S 2
		+15.5 / +9.0	16.5	C 28 H 27 O N 2 Cl 2 F 2 S 2
		+0.7 / +0.4	12.0	C 24 H 29 O 3 N 3 Cl 2 F 2 S 2
		-4.2 / -2.4	21.5	C 30 H 22 O 3 N 2 F 3 S 2
		+17.5 / +10.2	22.0	C 29 H 20 O 3 N 3 F 3 S 2
		-5.0 / -2.9	17.0	C 27 H 25 O 2 N 3 Cl F 3 S 2
		+16.7 / +9.7	17.5	C 26 H 23 O 2 N 4 Cl F 3 S 2
		+13.5 / +7.8	12.5	C 25 H 28 O 2 N 2 Cl 2 F 3 S 2
		-5.9 / -3.4	12.5	C 24 H 28 O N 4 Cl 2 F 3 S 2
		-10.8 / -6.2	22.0	C 30 H 21 O N 3 F 4 S 2
		+10.9 / +6.3	22.5	C 29 H 19 O N 4 F 4 S 2
		+7.8 / +4.5	17.5	C 28 H 24 O N 2 Cl F 4 S 2

Figure S69: Mass Spectrum of **BTP-9**

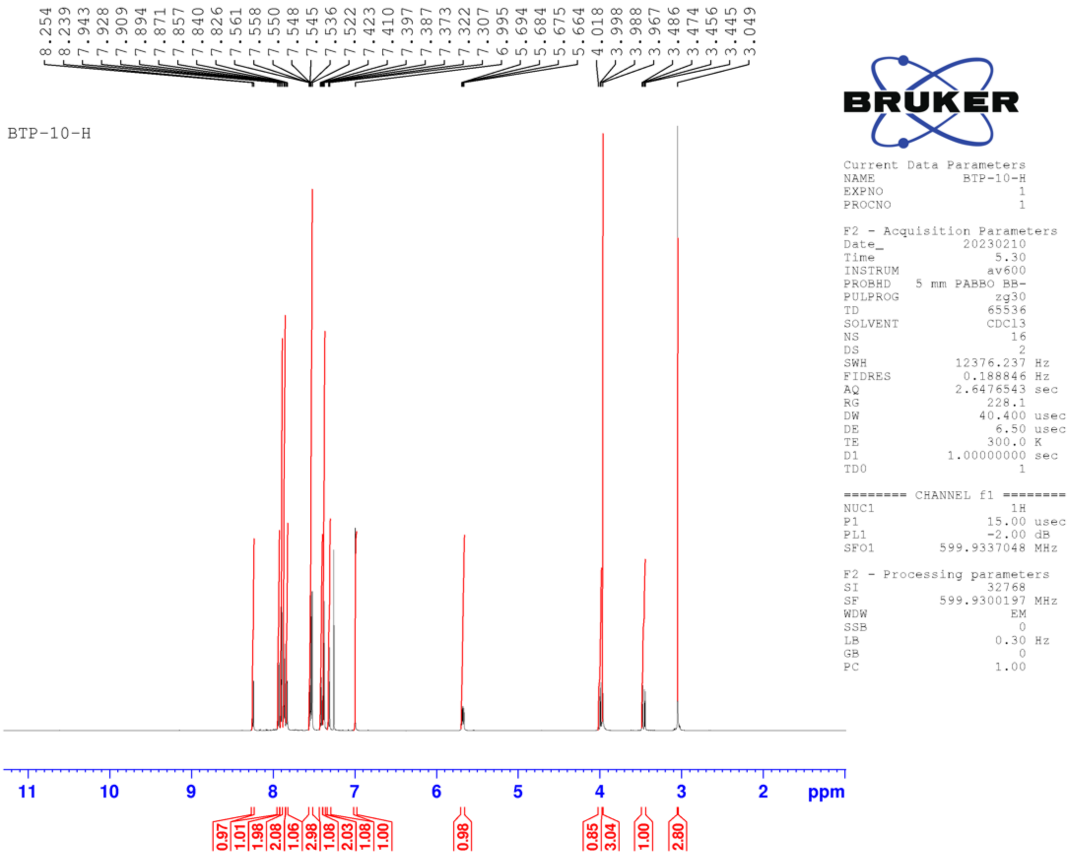

Figure S70: ^{1}H NMR Spectrum of **BTP-10**

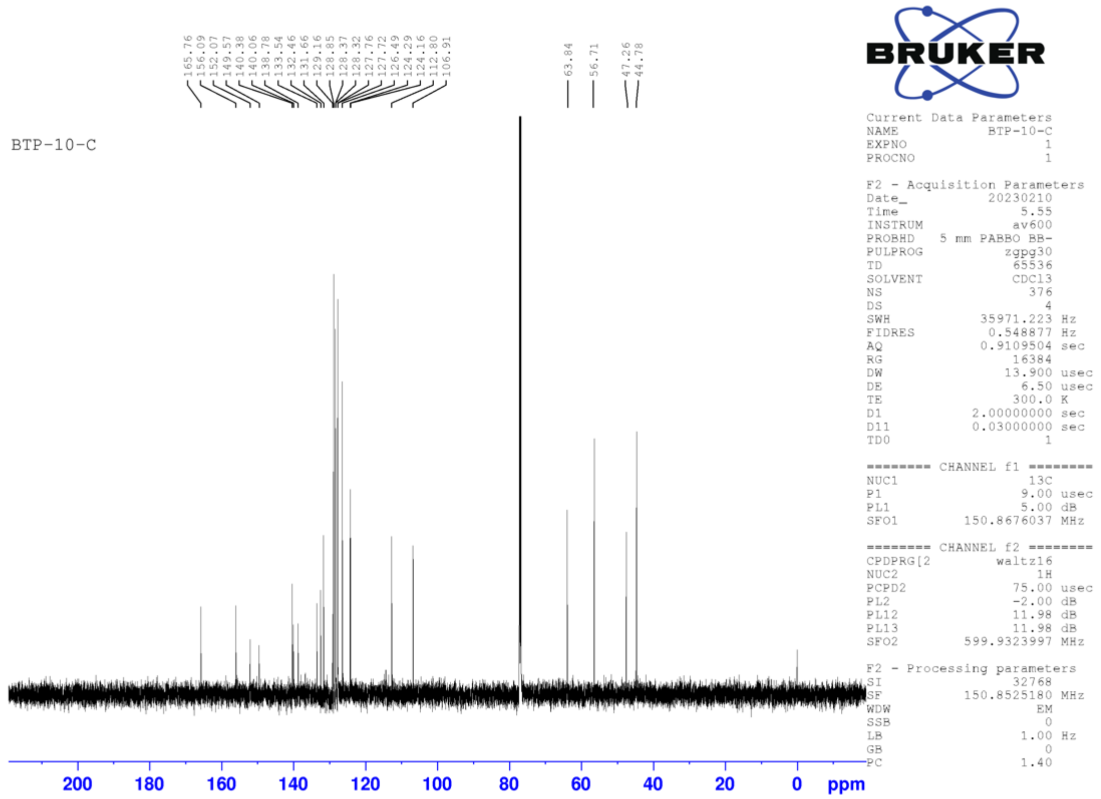

Figure S71: ^{13}C NMR Spectrum of **BTP-10**

[Elemental Composition] Page: 1
Data : gousei273 Date : 10-Feb-2023 14:32
Sample: BTP-10/sever belgin
Note : NBA
Inlet : Direct Ion Mode : FAB+
RT : 1.00 min Scan#: (2,8)
Elements : C 100/0, H 100/0, O 4/2, N 4/2, Cl 2/0, S 3/1
Mass Tolerance : 20ppm, 10mmu if m/z < 500, 20mmu if m/z > 1000
Unsaturation (U.S.) : -0.5 - 150.0

Observed m/z	Int%	Err[ppm / mmu]	U.S.	Composition
573.0938	97.8	+5.1 / +2.9	30.5	C 36 H 17 O 4 N 2 S
		-14.5 / -8.3	30.5	C 35 H 17 O 3 N 4 S
		-17.7 / -10.2	25.5	C 34 H 22 O 3 N 2 Cl S
		+4.2 / +2.4	26.0	C 33 H 20 O 3 N 3 Cl S
		-18.6 / -10.6	21.0	C 31 H 25 O 2 N 3 Cl 2 S
		+3.4 / +1.9	21.5	C 30 H 23 O 2 N 4 Cl 2 S
		-0.8 / -0.5	26.5	C 33 H 21 O 4 N 2 S 2
		-1.7 / -1.0	22.0	C 30 H 24 O 3 N 3 Cl S 2
		+17.1 / +9.8	17.5	C 28 H 27 O 3 N 2 Cl 2 S 2
		-2.5 / -1.4	17.5	C 27 H 27 O 2 N 4 Cl 2 S 2
		-6.7 / -3.8	22.5	C 30 H 25 O 4 N 2 S 3
		+15.2 / +8.7	23.0	C 29 H 23 O 4 N 3 S 3
		-7.5 / -4.3	18.0	C 27 H 28 O 3 N 3 Cl S 3
		+14.4 / +8.3	18.5	C 26 H 26 O 3 N 4 Cl S 3
		+11.2 / +6.4	13.5	C 25 H 31 O 3 N 2 Cl 2 S 3
		-8.4 / -4.8	13.5	C 24 H 31 O 2 N 4 Cl 2 S 3

Figure S72: Mass Spectrum of **BTP-10**
